# Similarity based enzymatic retrosynthesis†

**DOI:** 10.1039/d2sc01588a

**Published:** 2022-04-26

**Authors:** Karthik Sankaranarayanan, Esther Heid, Connor W. Coley, Deeptak Verma, William H. Green, Klavs F. Jensen

**Affiliations:** Department of Chemical Engineering, Massachusetts Institute of Technology 77 Massachusetts Avenue Cambridge Massachusetts 02139 USA kfjensen@mit.edu; Institute of Materials Chemistry, TU Wien 1060 Vienna Austria; Computational and Structural Chemistry, Discovery Chemistry, Merck & Co., Inc. Kenilworth NJ 07033 USA

## Abstract

Enzymes synthesize complex natural products effortlessly by catalyzing chemo-, regio-, and enantio-selective transformations. Further, biocatalytic processes are increasingly replacing conventional organic synthesis steps because they use mild solvents, avoid the use of metals, and reduce overall non-biodegradable waste. Here, we present a single-step retrosynthesis search algorithm to facilitate enzymatic synthesis of natural product analogs. First, we develop a tool, RDEnzyme, capable of extracting and applying stereochemically consistent enzymatic reaction templates, *i.e.*, subgraph patterns that describe the changes in connectivity between a product molecule and its corresponding reactant(s). Using RDEnzyme, we demonstrate that molecular similarity is an effective metric to propose retrosynthetic disconnections based on analogy to precedent enzymatic reactions in UniProt/RHEA. Using ∼5500 reactions from RHEA as a knowledge base, the recorded reactants to the product are among the top 10 proposed suggestions in 71% of ∼700 test reactions. Second, we trained a statistical model capable of discriminating between reaction pairs belonging to homologous enzymes and evolutionarily distant enzymes using ∼30 000 reaction pairs from SwissProt as a knowledge base. This model is capable of understanding patterns in enzyme promiscuity to evaluate the likelihood of experimental evolution success. By recursively applying the similarity-based single-step retrosynthesis and evolution prediction workflow, we successfully plan the enzymatic synthesis routes for both active pharmaceutical ingredients (*e.g.* Islatravir, Molnupiravir) and commodity chemicals (*e.g.* 1,4-butanediol, branched-chain higher alcohols/biofuels), in a retrospective fashion. Through the development and demonstration of the single-step enzymatic retrosynthesis strategy using natural transformations, our approach provides a first step towards solving the challenging problem of incorporating both enzyme- and organic-chemistry based transformations into a computer aided synthesis planning workflow.

## Abstract 2

Using a large database of enzymatic transformations, similarity based enzymatic retrosynthesis search algorithm takes a holistic approach to enzymatic synthesis planning to yield a diverse set of single-step suggestions.

## Graphical abstract

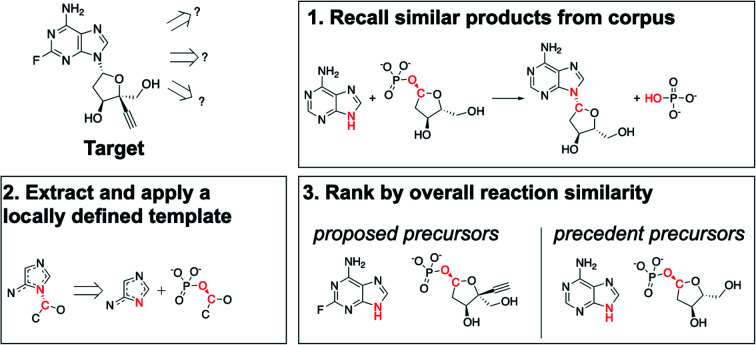

## 1 Introduction

Biocatalysis,^1–4^ metabolic engineering,^5–10^ and *in vitro* reconstitution of metabolic pathways^11,12^ use enzymes to catalyze a series of transformations to yield a desired small molecule or natural product, *e.g.* commodity chemicals and pharmaceutical agents. Enzymes are an important tool in a process chemist's toolkit as they catalyze selective transformations under mild conditions in a safe and sustainable fashion. Because many enzymes function in aqueous conditions, it is often feasible to carry out several reactions in a single pot to avoid purifying intermediates and/or overcome equilibrium constraints.^12^ Further, enzymes present an economic alternative to precious metal catalysts, *e.g.* rhodium, for asymmetric catalysis.^1,13^ Precious metals are expensive, and scarce; removal of metals from the final product is expensive, and there is a significant environmental cost associated with mining. On the other hand, the price of enzymes is stable, predictable, and more amenable to economic modeling. Finally, enzymatic synthesis routes can use renewable chemicals, *e.g.* glucose, *in lieu* of fossil fuels as starting materials to manufacture commodity chemicals, *e.g.* 1,4-butanediol^5^ and branched chain higher alcohols.^6^ This promotes sustainable production while avoiding cost fluctuations associated with fossil fuels.

Enzymatic syntheses^2,4,7–12^ of complex natural product analogs are greener and more efficient compared to their chemo-catalytic counterparts. For instance, the investigational HIV treatment Islatravir is manufactured using nine enzymes from simple achiral building blocks^2^ (Fig. S1†). The entire reaction sequence occurs under mild conditions, without requiring the purification of intermediates. As a consequence of the stereo- and chemo-selectivity associated with the enzymes, protecting groups are not necessary, and the overall number of steps is less than half compared to previous syntheses of this target. In other cases, the structural complexity of natural products hinders the development of practical, organic synthetic routes, leaving enzymatic routes as the only source of their commercial production.^7^

In a series of landmark studies, directed evolution has effectively been used to customize enzymes for small molecule synthesis by optimizing for the desired properties of interest-including activity in non-native environments,^14^ activity on non-natural substrates,^1^ and non-native catalytic activity^15^ including enantioselectivity.^16^ In parallel, *de novo* enzyme design has been used to develop catalysts for transformations not previously seen in nature.^17^ Taken together, both methods demonstrate promise for the notion that enzyme chemistry is generalizable beyond previously observed and cataloged reactions in databases.

To harness the value offered by enzymes, tools that are capable of generalizing known enzyme chemistry to propose retrosynthetic routes to a given target are valuable. The increasing availability of reaction corpora and algorithms for efficient search have enabled development of such computer aided synthesis planning (CASP) tools in enzymatic synthesis. First, RetroBioCat enables biocatalytic reaction planning using a set of manually curated reaction templates applicable to biocatalysis.^18^ Further, RetroBioCat enables enzyme selection by measuring chemical similarity against a manually curated enzymatic reaction database. Manual encoding of reaction rules and examples relies on intuition and experience of a small number of chemists, which complicates scaling of the approach. It is difficult to define the full substrate scope of every class of enzymatic reactions through the manual curation of an enzymatic reaction database. In a second study, RetroPath RL enables enzymatic synthesis planning in the context of metabolic engineering.^19,20^ In RetroPath RL, reaction templates are extracted algorithmically to a fixed diameter, and chemical and biological scores are utilized to evaluate substrate promiscuity of enzymes. Despite this success, algorithmic extraction and application of templates did not provide consistent handling of stereochemistry in reactions, despite the importance of stereochemistry in enzyme catalyzed transformations. Further, the substrate promiscuity of enzymes was treated as a hyperparameter; the promiscuity thresholds were set and tested using small validation (O(10^1^) compounds)- and test (O(10^2^) compounds)-sets, respectively. The generalizability of RetroPath RL to the significantly larger chemical space of enzymatically accessible compounds was not tested and is, therefore, unknown.

Both RetroBioCat and RetroPath RL use retrosynthetic templates that are locally defined pattern matching rules, lacking an understanding of what is present in the rest of the molecule. Therefore, a proposed retrosynthetic suggestion can be unviable in the forward direction (*e.g.* due to unfavorable steric or electronic effects). Once a synthetic route has been proposed, it is important to evaluate each step in the forward direction to identify these challenges. To facilitate such evaluation, Kreutter *et al.*^21^ developed an enzymatic transformer model to predict enzyme-catalyzed reaction products using input information about both reactants and enzymes. This model can be used to predict which substrates might be converted by a given enzyme. However, model performance was limited by database size and was lower with enzymes for which only few examples were available in the knowledge base. Currently, many enzymatic reaction databases (*e.g.* Reaxys, Rhea) catalog a limited number of substrates for every enzyme, often the known natural substrates. Data associated with a large library of substrates screened against a single enzyme are rarely available in a format suitable for model training, except in few well-represented, popular cases (*e.g. Candida antarctica* lipase B). Therefore, this transformer model needs to be complemented by alternative approaches that are capable of generalizing well using only currently available limited and poorly represented datasets.

The earliest CASP tools in organic synthesis were presented over 50 years ago.^22,23^ Since then, CASP tools in organic chemistry have been meaningfully explored by a number of studies,^24–27^ and they can serve as important case studies for the development of enzymatic analogues. First, advances in template extraction and application in organic synthesis applications facilitate consistent handling of stereochemistry for retrosynthesis. For example, RDChiral is designed to enforce the introduction, destruction, retention, and inversion of chiral tetrahedral centers as well as *cis*/*trans* configuration of double bonds.^28^*In lieu* of extracting reaction templates to a fixed diameter, RDChiral incorporates specific substructural motifs that are likely to contribute to overall chemical reactivity. Second, overall molecular similarity has been used to propose and rank one-step retrosynthetic disconnections based on analogy to precedent reactions.^29^

A core task in computer aided enzymatic synthesis planning is the ability to perform one-step retrosynthesis. The goal of single step retrosynthesis is to detect experimentally tractable disconnection sites in a single target compound, suggest the correct chemical reactions, candidate enzymes, and precursors needed to recreate those sites, and finally rank them by the probability of success. Experienced biochemists could then utilize these results for idea generation while planning enzymatic synthesis routes. A single step retrosynthetic search can also be applied in a recursive fashion to yield a multi-step synthesis plan. Here, a high-level strategy helps guide the retrosynthetic search towards the desired starting materials. For biocatalysis, the desired starting materials are simple, achiral, commercially available building blocks. On the other hand, for metabolic engineering, the desired starting materials are intermediates present in the host organism's metabolic pathways (*e.g.* glycolysis, the citric acid cycle). Herein, we describe a single step retrosynthesis strategy to addresses these challenges.

In this work, we make three specific contributions towards computer aided enzymatic synthesis planning. First, RDEnzyme, expands further on RDChiral, to facilitate algorithmic extraction and application of stereochemically consistent enzymatic reaction templates. Second, RDEnzyme and overall molecular similarity are utilized to propose one-step retrosynthetic disconnections based on analogy to precedent reactions in an enzymatic reaction database, that is curated for this study. Due to the algorithmic nature of our one-step retrosynthesis module, it will continue to propose reactions for product molecules that are obviously out-of-scope of the entire reaction database. Further, poorly ranked suggestions, with low overall molecular similarity scores, propose reactions that are chemically dissimilar to the precedent reactions in the database without any consideration for experimental feasibility. Therefore, a quality control check to filter such suggestions is necessary. Consequently, we train and evaluate a statistical model that is capable of discriminating between reaction pairs belonging to homologous enzymes and evolutionarily distant enzymes. This model is capable of understanding patterns in enzyme promiscuity to evaluate the likelihood of experimental evolution success. Further, it generalizes reactions in smaller databases (*e.g.* Rhea), in a fashion complementary to the transformer model developed by Kreutter *et al.*^21^ that requires larger databases.

This tool is exhibited using the public, publishable dataset RHEA.^30,31^ This reaction database is used for primary amino acid sequence annotation in UniProt. Therefore, it was also selected because it captures enzyme reaction diversity in natural enzymes: *ca.* 24 500 reactions describe ∼21.6 million enzymes in UniProt and ∼220k enzymes in SwissProt. Because our approach is designed to propose retrosynthesis suggestions within the chemical scope of the database, our demonstrations will primarily use naturally occurring molecules and their analogs. However, the algorithm(s) can also applied to private, commercial databases like Reaxys,^32^ SciFinder,^33^ or proprietary electronic lab notebooks, all containing enzymatic reactions previously used in organic chemistry.

## 2 Results

### 2.1 Overview

**Fig. 1:**
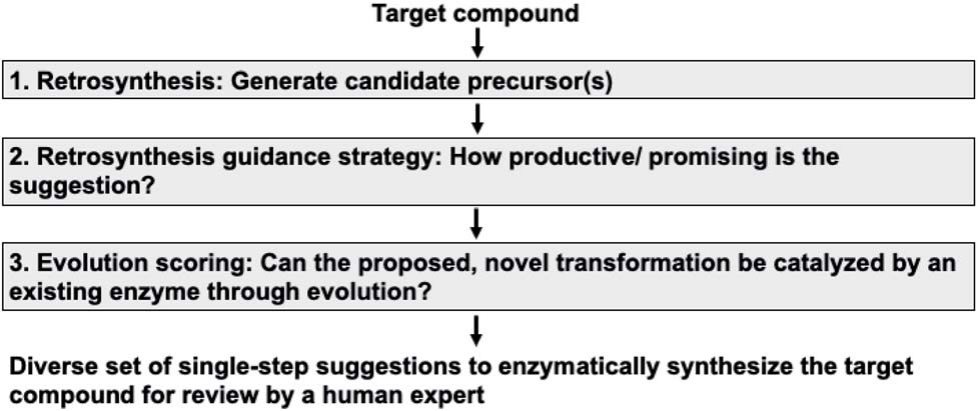
The anatomy of the retrosynthesis tool. In this work, we make two major contributions. First, components (1) (‘Retrosynthesis’) and (2) (‘Retrosynthesis guidance strategy’) take a holistic approach to enzymatic synthesis planning by using a large database of enzymatic transformations. They yield a diverse set of single-step suggestions to enzymatically synthesize a target compound. Then, component (3) (‘Evolution scoring’) uses a statistical model to avoid candidate reactions that are unlikely to work experimentally.

Our enzymatic retrosynthesis tool comprises three major components (Fig. 1, tasks 1–3):

1. A method to facilitate single-step retrosynthesis.

2. A tool to guide the retrosynthetic search towards the desired starting materials.

3. A method for evolution scoring, *i.e.* a preference for transformations likely to be experimentally evolvable.

#### 2.1.1. Single-step retrosynthesis

Our retrosynthesis strategy asks the question: *how have chemically similar molecules been synthesized previously by enzymatic reactions?* By ensuring chemical similarity between proposed and precedent reaction molecules, this approach intends to propose enzymes with binding pockets likely to accommodate the novel substrates, either naturally or through directed evolution.

The reactions from RHEA were atom mapped computationally.^34^ Table S1† in the ESI lists difficulties encountered and resulting number reactions not atom mapped. All enzymatic transformations were considered to be reversible because enzymes can potentially be combined using multistep biocatalytic cascades to overcome an unfavorable equilibrium.^2^ Further, reactions containing wildcard atoms, which represented an unknown chemical R-groups or biological entities (*e.g.* protein, tRNA, histones), were removed from the dataset. Such reactions are not conducive to retrosynthesis by chemical similarity due to missing chemical information. Similarly, when wildcards represent biological entities (*e.g.* tRNA), the reactions are likely to be outside the desired scope of small molecule retrosynthesis. As a consequence of computational atom mapping, ∼7% of the dataset had multiple atom mapping solutions (Table S2†), which were enumerated to ensure that every solution was considered during the retrosynthetic analysis (additional detail in ESI†).

**Fig. 2:**
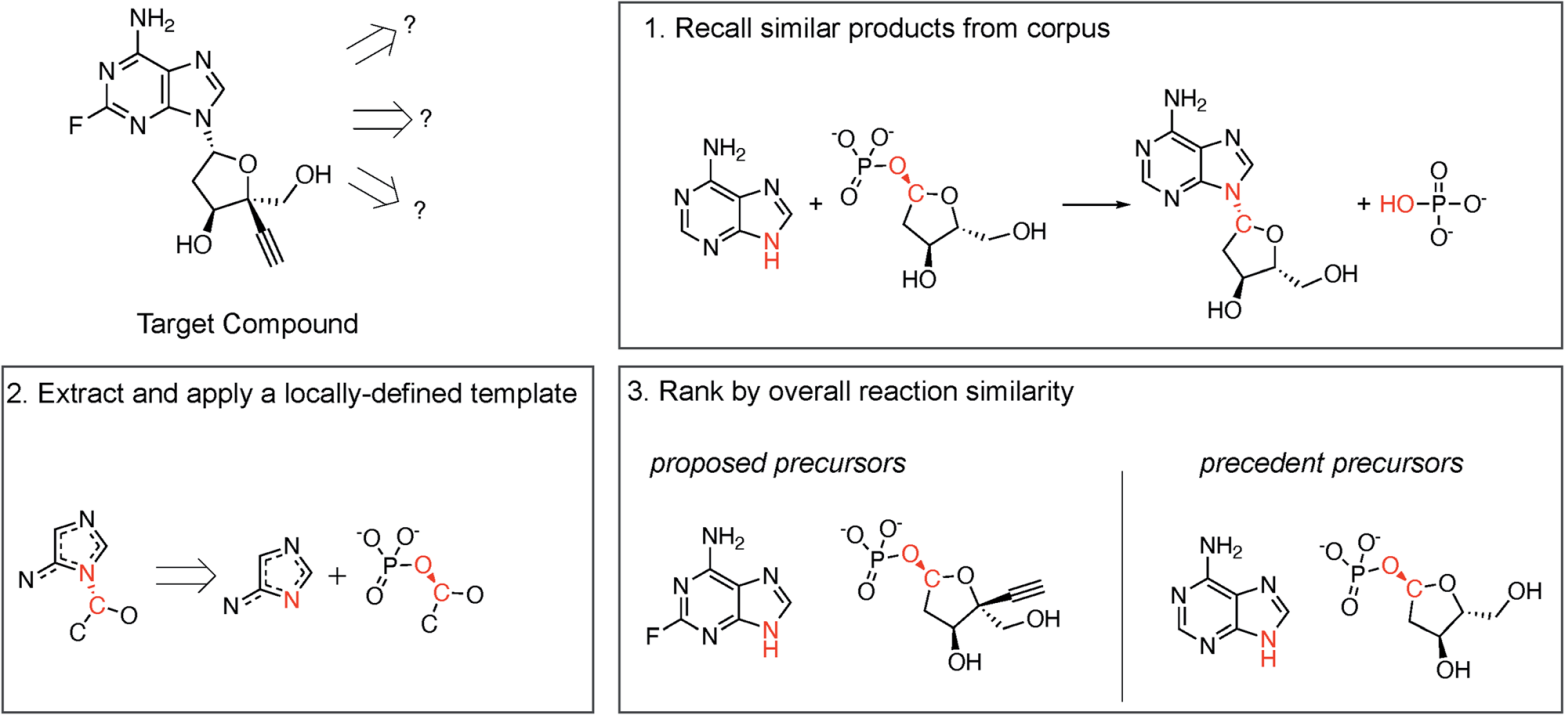
Retrosynthesis by chemical similarity. (1) Molecular similarity is utilized to propose one-step retrosynthetic disconnections based on analogy to precedent products in an enzymatic reaction database (in this example, similarity_product_ = 0.77). (2) A generalized retrosynthetic template is extracted from the precedent reactions and applied to the desired product. (3) The proposed *vs.* precedent precursor similarity is calculated (in this example, similarity_reactant_ = 0.81). Proposed reactions are scored and ranked by overall molecular similarity, defined as similarity_reactant_ × similarity_product_ (in this example, similarity_overall_ = 0.62), to the precedent reaction.

Following procedures adapted from Coley *et al.*,^29^ molecular similarity is utilized to propose one-step retrosynthetic disconnections based on analogy to precedent products in an enzymatic reaction database (Fig. 2, step 1). Then, a generalized retrosynthetic template is extracted from the precedent reactions and applied to the desired product using RDEnzyme (Fig. 2, step 2). By holding the reaction template constant across the precedent and proposed reactions, we identify enzymes capable of catalyzing the desired transformation. Finally, proposed reactions are scored and ranked by overall molecular Dice^35^ similarity, defined as (similarity_reactants_ × similarity_products_), to the precedent reaction (Fig. 2, step 3). This approach is designed to capture the stereo-, regio-, and chemo-selectivity commonly associated with enzymes. As control, we randomly selected precedent products (step 1) and randomly ranked proposed reactions (step 3). We describe the method in greater detail in the ESI.†

Following procedures adapted from Segler *et al.*,^26^ top-k accuracy analysis is utilized to evaluate the performance of the retrosynthesis algorithm to generalize existing reactions to propose new ones. Transformation rules from all enzymatic reactions (14 013 total) were extracted using RDEnzyme. These transformation rules contain atoms and bonds that changed in the course of the reaction, and a varying number of neighbors determined using a fixed distance and/or heuristics that decide which neighboring atoms are relevant. Reactions with rules that occurred at least three times were kept (6973 total). Then, the dataset was split randomly into train : validation : test splits as 5578 (80%) : 697(10%) : 698(10%). Given the product of reactions in RHEA/UniProt in the test split, we measured the program's ability to recover and rank highly the recorded reactants without having seen the reaction previously (top-k accuracy). The ESI† describes the evaluation procedure in greater detail.

Different combinations of fingerprint settings and similarity metrics were evaluated using the validation dataset (Fig. S2†). The top-k accuracy is not a strong function of the settings tested, as previously observed in organic retrosynthesis.^29^ Therefore, Morgan fingerprint (radius = 2, with features) and Dice similarity were used for enzymatic retrosynthesis.

**Fig. 3:**
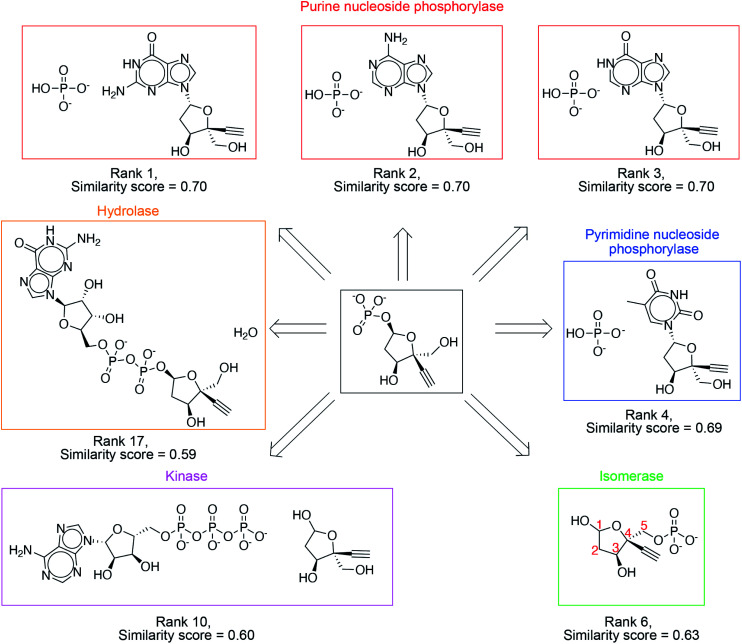
High ranking suggestions for an intermediate in Islatravir enzymatic synthesis pathway. Diverse enzymatic reaction classes are suggested including purine/pyrimidine nucleoside phosphorylase, isomerase, kinase, and hydrolase.

The success criterion is met within the top 3, top 10 and top 20 suggestions 39%, 71% and 86% of the time, respectively (Table 1). High ranking suggestions for an intermediate in Islatravir enzymatic synthesis pathway (Fig. 3) demonstrate the use of the one-step retrosynthesis tool for idea generation. This intermediate compound and proposed suggestions are not part of the reaction database, and therefore, this is intended to highlight the generalization capability of the platform. Ranks 1–3 suggest the stereoselective displacement of purine nucleobase with phosphate. Rank 4 involves the stereoselective displacement of pyrimidine nucleobase with phosphate. Rank 6 involves the use of an isomerase to transfer the phosphate group from the 5-position to 1-postion on the sugar with the desired stereospecificity. This suggestion was used in the development of the enzymatic synthesis route to Islatravir.^2^ Rank 10 suggests the use of a kinase enzyme to stereo- and regio-selectively transfer the phosphate group from ATP to the sugar. Rank 17 suggests the use of a hydrolase enzyme to yield the desired sugar 1-phosphate target. Our platform takes a holistic approach to enzymatic synthesis planning by using a large database of enzymatic transformations and primary amino acid sequences. Therefore, it yields a diverse set of suggestions that can potentially be implemented using the amino acid sequence information available. Several other examples from the test set are show in Fig. S3–S10.†

**Table 1:** Similarity based model performance on test set. As control, templates were randomly selected and ranked. Mean and standard deviation of three independent, random runs are shown

Top-*n*	Similarity	Random
	Average (%)	Average ± SD (%)
1	17	4 ± 1
3	39	9 ± 2
5	51	13 ± 2
10	71	17 ± 2
20	86	21 ± 2

**Fig. 4:**
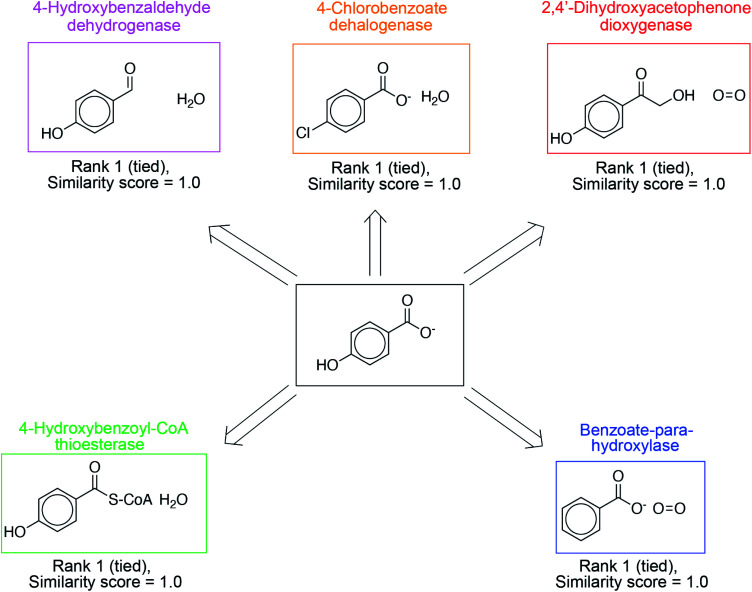
The algorithm highly ranks suggestions known to synthesize the target compound. An example search for the target compound 4-hydroxybenzoate, already present as a product in the reaction database, is shown. Multiple enzymes with recorded transformations producing the desired target are suggested including 4-hydroxybenzaldehyde dehydrogenase, 4-chlorobenzoate dehalogenase, 2,4′-dihydroxyacetophenone dioxygenase, benzoate-*para*-hydroxylase, and 4-hydroxybenzoyl-CoA thioesterase. These suggestions have high similarity scores, and therefore, they are ranked highly.

In addition to proposing new reactions, the ability to prioritize relevant, existing reactions in the database is equally important. These suggestions are more easily implementable, without the need for directed evolution. The overall reaction similarity-based ranking naturally lends itself to this prioritization; suggestions with exact precedents in the database are ranked highly. In Fig. 4, we present such suggestions for 4-hydroxybenzoate, a product molecule in the database, and a diverse set of synthesis strategies are identified to yield this target. 4-Hydroxybenzaldehyde dehydrogenase catalyzes the oxidation of the 4-hydroxybenzaldehyde to 4-hydroxybenzoate. 4-Chlorobenzoate dehalogenase catalyzes the dehalogenation of 4-chlorobenzoate to 4-hydoxybenzoate. 2,4′-Dihydroxyacetophenone dioxygenase catalyzes the cleavage of 2-hydroxy-1-(4-hydroxyphenyl)ethenone to 4-hydroxybenzoate and formate. Benzoate-*para*-hydroxylase catalyzes the hydroxylation of benzoic acid into 4-hydroxybenzoate. Lastly, 4-hydroxybenzoyl-CoA thioesterase catalyze the hydrolysis of 4-hydroxybenzoyl-CoA to 4-hydroxybenzoate and CoA. These suggestions have a similarity score of 1.0 on the scale [0–1] because of the presence of an exact literature precedent. As a result, they are ranked highly. Other examples are shown in Fig. S11–S14.†

#### 2.1.2. Retrosynthesis guidance strategy for synthetic and biosynthetic applications

For biocatalysis applications, chemical similarity based ranking is supplemented by an evaluation of the overall productivity of the suggestion (*i.e. is the retrosynthetic suggestion leading the synthesis plan towards simple, commercially available building blocks?*). Synthetic Complexity Score (SCScore) of a molecule (ranging from 1–5) is a machine learnt quantity that correlates with the difficulty of producing a target molecule.^36^ In other words, molecules that are easy to synthesize have a low SCScore, while molecules that are harder to synthesize have a higher SCScore. When trained on the premise that reactants of published chemical reactions are on average synthetically less complex than their products, a neural network model can evaluate the SCScore of a molecule based on its chemical structure. The retrosynthesis guidance of the method in synthetic applications was evaluated in the original reference^36^ for organic transformations and recently by Finnigan *et al.*^18^ for enzymatic reactions.

In order to assess the productivity of the retrosynthetic suggestions, we used the difference in SCScores of the reactants and products (ΔSCScore), defined as SCScore_product_ − max(SCScore_reactants_). First, a retrosynthetic analysis was performed and proposed reactions ranked by overall molecular similarity scores (similarity_reactant_ × similarity_product_). Next, the reactants were evaluated to determine whether they were a commonly occurring biochemical molecules (see ESI† for database curation information). Such molecules were not included in the SCScore based analysis because they were likely to be commercially available/readily accessible through non-synthetic means (*e.g.* ATP, NADPH *etc.*) (Fig. S15†). Finally, ΔSCScore was computed and the resulting scores used for re-ranking suggestions for biocatalysis. The ESI† describes this procedure in greater detail.

For applications in metabolic engineering, our chemical similarity-based retrosynthesis approach guides the retrosynthesis strategy. Our database consists of examples of how enzymes synthesize small molecules through metabolism. Further, our approach asks the question: *how have similar molecules been synthesized in that database?* If a pathway to the molecule has previously been discovered and cataloged, the program will likely suggest that route without modification, among other possibilities. If it is a novel compound, then the program looks for routes to other chemically similar compounds and proposes applicable biosynthetic strategies.

#### 2.1.3. Evolution scoring

In order to identify experimentally feasible transformations amongst the large number of suggestions resulting from the one-step retrosynthesis module, we trained a statistical model to predict the likelihood that the proposed reaction can be evolved starting from the precedent reaction. We obtained a training dataset that was inexpensive, chemically diverse, and did not require laboratory resources by assuming that transformations associated with homologous enzymes provided examples of both reactive promiscuity and substrate promiscuity seen in enzymes.

We performed global pairwise alignment, as implemented by Biopython (bio.pairwise2),^37^ between two primary amino acid sequences. BLOSUM-62 (blocks of amino acid substitution matrix) substitution matrix was used. Gap-open and gap-extend penalties were set at 10 and 0.5, respectively, and gaps at the end of the alignment were not penalized. After sequence alignment, percent identity of the highest scoring alignment was computed as 
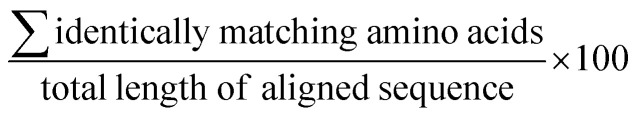
. To ensure our implementation in Biopython was setup appropriately, we compared it to EMBL-EBI's sequence analysis tool^38^ which uses the Needleman–Wunsch algorithm to perform its global sequence alignment (Fig. S16 and Table S3†). Default settings were used (matrix: BLOSUM 62, gap open penalty: 10, gap extend penalty: 0.5, end gap penalty: false).

We aimed to obtain examples of reactions associated with homologs *versus* evolutionarily distant enzymes. Reaction SMILES were obtained from RHEA.^30^ Further, reaction SMILES containing unknown, wildcard atoms were ignored from the analysis to ensure satisfactory data quality. Similarly, only manually annotated and reviewed primary amino acid sequences were used from UniProt/SwissProt.^31^ If multiple amino acid sequences were annotated for a given reaction, one exemplary sequence corresponding to each reaction was randomly selected for the analysis.

We identified 18.8k homologous enzyme reaction pairs, and balanced the dataset with 19.3k evolutionarily distant reaction pairs. While ensuring sequence length was longer than 100 amino acids, sequence identities greater than 52% were considered to be homologous and labeled as positive, and sequence identities less than 15% were labeled as negative^39^ (Fig. S17 and S18†). To assign labels, the dataset was made binary as evolvable (score = 1) or not-evolvable (score = 0).

Given the limited amount of data available for training the model, it was important to ensure that the model could generalize without overfitting. Therefore, reaction similarity and overall molecular similarity of the pair of reactions were used as features to discriminate between homologs *vs.* evolutionarily distant enzymes.

Molecular similarity_overall_ was defined as the (molecular similarity_reactants_ × molecular similarity_products_). Given a pair of reactions, the overall molecular similarity was calculated by representing the reactants (or products) of each reaction as Morgan fingerprints (radius = 2, using chirality, and using features) and quantifying Dice similarity (see ESI† for implementation details). Since the reactions were considered reversible, every possible combination of reactants and products were used to compute molecular similarity_overall_ and the maximum score was used as the representative feature.

Reaction similarity requires a reaction fingerprinting technique and a similarity metric. For this study, reaction fingerprints were computed as the difference between reactant and product fingerprints. Further, Dice similarity quantified similarity between fingerprint vectors (Fig. S19–S22†).

**Fig. 5:**
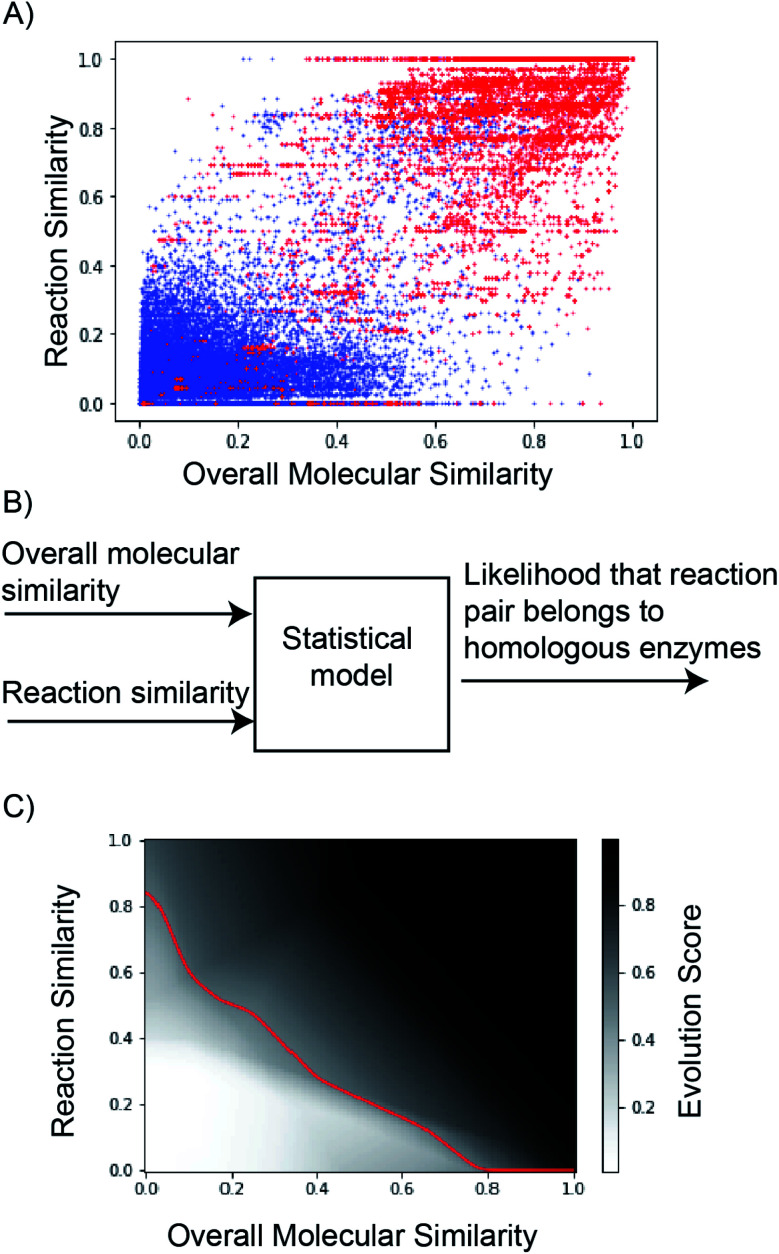
(A) Reaction similarity *vs.* overall molecular similarity of 38 126 reaction pairs associated with homologs *versus* evolutionarily distant enzymes. Red corresponds to reaction pairs of homologous enzymes (total = 18 809 pairs). Blue corresponds to reaction pairs of evolutionarily distant enzymes (total = 19 317 pairs). (B) ‘Reaction similarity’ and ‘Overall molecular similarity’ of a pair of reactions was used to discriminate between homologs *vs.* evolutionarily distant enzymes. (C) The output of the neural network (‘Evolution Score’) is plotted as a function of reaction- and overall molecular-similarity feature values. The decision boundary (at evolution score = 0.5) is shown in red. The model has learned that reaction pairs with high reaction- and overall molecular-similarity scores are likely evolvable.

This study explores ‘multi-layer perceptron’ to discriminate reaction pairs as evolvable or not-evolvable. However, we emphasize our goal is not to exhaustively understand the different approaches to make this judgement, but to find a tool that can promote promising suggestions and avoid poor ones. Reaction similarity- and overall molecular similarity-scores were used as model inputs (Fig. 5A and B). The dataset was randomly split into training : validation : test sets (80 : 10 : 10). To verify that there was no leakage of training data into the validation/test data, we verify that every pair of RHEA identifiers (‘RHEA ID’) in our dataset was unique to ensure every reaction pair was unique. Binary cross entropy loss and a modified stochastic gradient descent algorithm were used to train the model. The output score in the range 0–1 reflected the probability that the reactions were evolvable.

Hyperparameter optimization was performed using the validation dataset to determine the number of hidden layers and the number of nodes per hidden layer (Table S4†). The final model requires 37 parameters, described in detail in Table S5.† Three hidden layers with ReLu activation were used prior to the sigmoid output layer (Fig. S23†). The resulting model achieves a receiver operating characteristic curve-area under the curve (ROC-AUC) of 0.98 on the test data (Fig. S24†). The classification threshold probability was set at 0.5 (Fig. S25†). The model's output, evolution score, ranges from 0 to 1, and reaction pairs with high reaction similarity- and overall molecular similarity-scores tend to have a high evolution score (Fig. 5C).

**Fig. 6:**
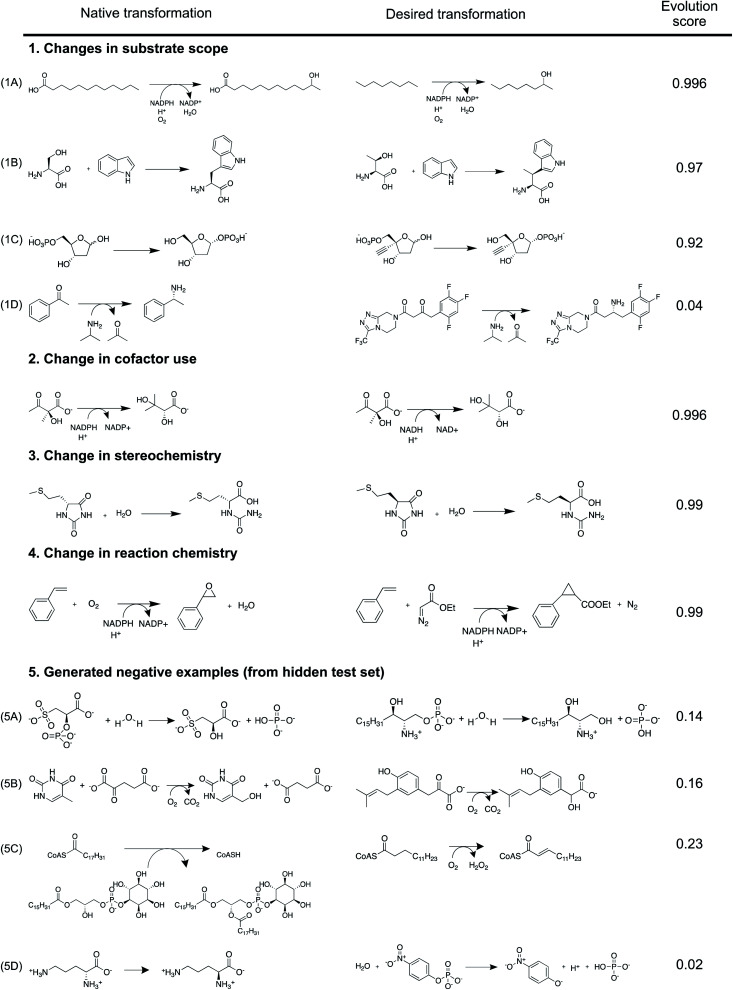
Using case studies that were in fact experimentally implemented prior to our model, we demonstrate our model understands that directed evolution of enzymes (1) optimizes their catalytic activity towards new substrates,^2,40,41^ (2) alters their cofactor dependence,^42^ (3) inverts their enantioselectivity,^43^ and (4) makes them catalyze new chemical reactions.^44^ Using generated negative examples, we illustrate types of reaction proposals that the model is likely to avoid. In (5A) and (5B), desired *vs.* proposed transformations significantly overlap in reaction chemistry, but they accommodate drastically different substrates. Both transformations in (5C) have similar substrates (*i.e.* acyl-CoA), but catalyze different reactions. In (5D), neither substrate nor reaction chemistry overlap between the native- and desired-transformations. The model intentionally discourages making drastic changes to the native transformation to yield the desired transformation, sometimes resulting in false negatives similar to (1D).^1^

Evolution score can be interpreted as the likelihood that the proposed reaction is evolvable starting from the precedent reaction in database. Implicitly, the model understands that directed evolution of enzymes optimizes their catalytic activity towards new substrates, alters their cofactor dependence, inverts their enantioselectivity, and makes them catalyze new chemical reactions. Here, we demonstrate this understanding using selected case studies that were in fact experimentally implemented prior to our model (Fig. 6). Further, curated examples from the test set to demonstrate this understanding are also shown in Fig. S26–S32.†

The model successfully understands that enzymes can tolerate minor chemical changes to their substrates. First, Glieder *et al.* converted a medium chain (*e.g.* C12) fatty acid monooxygenase into a catalyst for the conversion of medium chain alkanes (*e.g.* C8) to alcohols^40^ (Fig. 6(1A)). Second, Herger *et al.* engineered a subunit of tryptophan synthase to accommodate l-threonine, *in lieu* of l-serine, in the β-substitution reaction to yield (2*S*,3*S*)-β-methyltrytophan^41^ (Fig. 6(1B)). Third, Huffman *et al.* enabled *E. coli* phosphopentomutase to accommodate an unnatural substrate containing an additional ethynyl group^2^ (Fig. 6(1C)). These experimentally implemented substrate scope changes were correctly predicted by the model to have high (>0.92) evolution scores. Because our similarity-based retrosynthesis tool usually proposes suggestions with altered substrates, multiple such examples (Fig. 6(1A–1C)) are discussed and emphasized.

Beyond substrate scope, the model is implicitly aware of other enzyme properties that can be altered by directed evolution. First, Bastian *et al.* altered the co-factor dependence of an enzyme so that it can rely on NADH, *in lieu* of NADPH^42^ (Fig. 6(2)). Second, May *et al.* inverted reaction enantioselectivity^43^ (Fig. 6(3)). Finally, Coelho *et al.* altered reaction chemistry of enzymes by starting from a cytochrome P450 catalyzed monooxygenation reaction to ultimately facilitate a cyclopropanation reaction^44^ (Fig. 6(4)). These experimentally implemented changes to enzyme properties were correctly predicted by the model to have high (>0.99) evolution scores.

Due to a lack of published negative results, we illustrate the pattern that the model seeks to avoid using our generated negative examples (Fig. 6(5)). Four negative examples from the test set at different limits of reaction- and overall molecular-similarity feature values are presented (Table S6†). First, the model discourages major chemical changes to the substrate even when the reaction chemistry is largely conserved. In Fig. 6(5A), both reactions are associated with phosphatases; however, the substrates are chemically dissimilar (Fig. S33†). Similarly in Fig. 6(5B), both reactions describe dioxygenases, but with chemically different substrates (Fig. S34†). Second, the model discourages major changes to reaction chemistry even when the substrate is largely held constant. For example, in Fig. 6(5C), both enzymes catalyze reactions on chemically similar acyl-CoA substrates (Fig. S35†). However, their reaction chemistries are different (*i.e.* acyl transfer *vs.* oxidation). Finally, the model discourages simultaneous changes to both reaction chemistry and substrate. Fig. 6(5D) describes two enzymes catalyzing different transformations on different substrates (Fig. S36†). These negative examples were correctly predicted by the model to have low (<0.23) evolution scores. These simple rules captured by the model discriminate with a test set accuracy of 94% (ROC-AUC = 0.98) on *ca.* 4000 examples. However, exceptionally promiscuous enzymes are not captured by this model (Fig. S31†).

This statistical model is designed to serve as a sanity check. It avoids suggestions proposed by similarity based retrosynthesis that are obviously out-of-scope of the entire reaction database (*e.g.* due to unfavorable steric or electronic effects). While it is suited for this purpose, there are some limitations in the broader context of enzyme engineering and directed evolution. First, with significant effort, it can be possible to make drastic chemical changes to reactions catalyzed by enzymes. For example, Savile *et al.* altered the substrate scope of a transaminase to recognize a complex ketone in place of its smaller native substrate for sitagliptin manufacture^1^ (Fig. 6(1D)). Our model discourages such suggestions owing to the complex challenges associated with such an enzyme engineering problem, despite it being tractable. Second, new enzymatic reactions that do not have any similar, natural precedents cannot be predicted by this model. For example, Siegel *et al.* describe the *de novo* computational design of enzymes catalyzing a Diels–Alder reaction, for which there are no known natural analogs.^17^ Completely new reactions with no precedents are unlikely to be captured using this model because it is intentionally designed to take advantage of a database of existing enzymatic transformations to evaluate and propose new reactions. Finally, because the model *only* understands general similarity patterns between reactions from a chemical perspective without detailed knowledge about any given enzyme (*e.g.* its binding pocket, catalytic site, reaction mechanism, kinetics, expressability, solubility, *etc.*), some false positive results were observed (*e.g.* Fig. S32†). Notwithstanding this limitation, the model fits its intended use during the initial stages of enzymatic synthesis planning, where detailed implementation plans might not be necessary.

#### 2.1.4. Enzymes used in synthetic applications

**Fig. 7:**
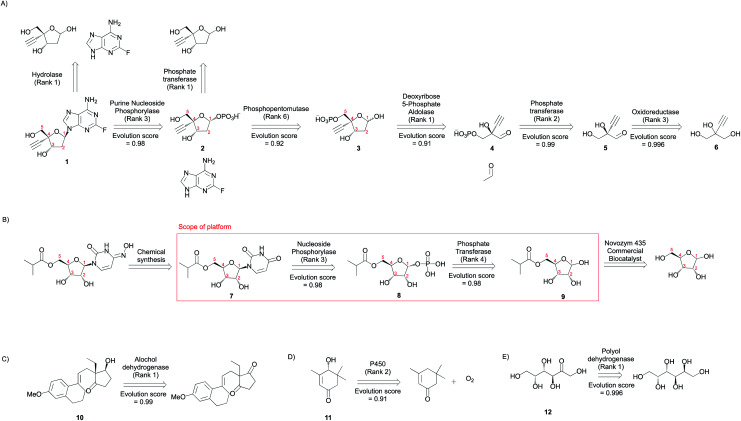
Synthetic applications. Multi-step synthesis plans for medicinal compounds (A) Islatravir, (B) Molnupiravir, (C) (13*R*,17*S*)-ethyl secol, (D) (*R*)-4-hydroxy isophorone, and (E) d-tagatose (Fig. S37–S46†). All experimentally implemented suggestions are shown along with an evolution score. The rank and evolution score capture the promising nature of suggestions that were implemented experimentally.

Islatravir,^2^ Molnupiravir,^4^ (13*R*,17*S*)-ethyl secol,^45^ (*R*)-4-hydroxy isophorone,^46^ and (d)-tagatose^47^ serve as model compounds to demonstrate this tool's capability to solve problems in biocatalysis. Further, these compounds were also selected because they can be synthesized using natural enzymatic transformations expected to be represented in our knowledge base from RHEA. Our one-step modules (retrosynthesis and evolution scoring) are applied in a recursive fashion to facilitate synthesis planning (Fig. 7 and S37–S46†). Importantly, none of these compounds appear as products in the knowledgebase from which suggestions are made.

The first suggestion for Islatravir is a purine nucleoside phosphorylase (rank = 3), which stereoselectively displaces the phosphate with a nucleobase to yield the (1*R*) diastereomer (1). The top ranking suggestion is a reverse hydrolysis reaction catalyzed by a hydrolase. However, the desired aqueous reaction condition is unlikely to result in a high yielding commercial process because of the equilibrium position. Second, a phosphopentomutase (rank 6) stereospecifically transfers the phosphate group from 5- to 1-position to yield the (1*R*) diastereomer (2). The top ranking suggestion is the transfer of a phosphate group from a donor (*e.g.* ATP) to the target using a kinase/phosphate transferase. This strategy potentially requires the *in situ* regeneration of the phosphate donor, and we note that a phosphate transferase is used in the synthesis of Molnupiravir. Third, a deoxyribose 5-phosphate aldolase (DERA) catalyzes the forward aldol reaction converting a glyceraldehyde 3-phosphate analogue and acetaldehyde to the sugar 5-phosphate (3). This new C–C bond forming reaction is stereoselective favoring the synthesis of (3*S*,4*R*) diastereomer. Finally, oxidation and phosphorylation reactions can convert the simple achiral building block 2-ethynylglycerol (6) to the enantiomerically enriched 2-ethynylglyceraldehyde 3-phosphate (4).

The first step for Molnupiravir is the conversion of the amidic carbonyl in the uracil ring to the corresponding oxime. This was accomplished chemically. Second, a nucleoside phosphorylase (rank 3) stereoselectively displaces the phosphate with a nucleobase to yield the (1*R*) diastereomer of the target (7). Third, a phosphate transferase/kinase (rank 4) stereo- and regio-selectively transfers the phosphate group from a donor (*e.g.* ATP) to the 1-OH of the accepting sugar (9) and yields the (1*R*) diastereomer (8). Finally, a commercial lipase catalyzes the selective esterification of the ribose sugar using an isobutyrl donor. Since this is a proprietary enzyme by Novozymes, its transformations are not present in our database. However, we hypothesize that 6-acetylglucose-deacetylase (RHEA: 18487) is a potential candidate to facilitate the esterification transformation because it yields a chemically similar product (Fig. S47†).

Many prominent examples of biocatalytic reactions in organic chemistry catalyze selective transformations to yield chiral compounds. Using a few illustrative examples, we demonstrate selectivity considerations captured by the approach. First, the algorithm proposes the reductive desymmetrization of ethyl secodione to (13*R*,17*S*)-ethyl secol (10). This proposed reaction is highly demanding in regio- and stereo-selectivity; theoretically, without control of selectivity, ten isomeric products could be obtained (including the over-reduction products). Further, RDEnzyme is also able to automatically capture the retrosynthetic destruction of a chiral center distant from the atoms participating in the reaction. Second, a cytochrome P450 monooxygenase is proposed to catalyze the regio- and stereo-selective oxidation of α-isophorone to (*R*)-4-hydroxy isophorone (11). Using example reactions from RHEA, the tool proposes this selective enzyme catalyzed carbon–hydrogen functionalization reaction with significant economic and environmental benefits over traditional synthetic methods. Finally, the tool takes advantage of the high regioselectivity associated with enzyme catalyzed reactions to propose the conversion from galactitol to d-tagatose (12). These examples highlight the tool's capability to propose advantageous enzyme catalyzed selective reactions.

#### 2.1.5. Enzymes used in metabolic engineering applications

**Fig. 8:**
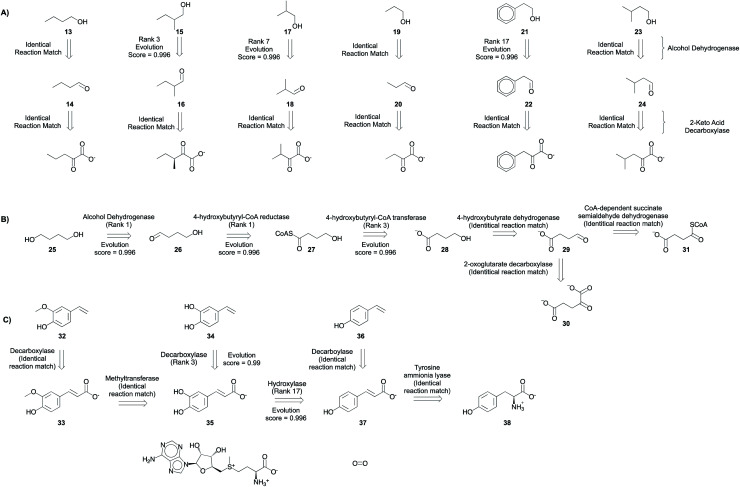
Metabolic engineering applications. Multi-step synthesis plans for commodity chemicals (A) branched chain higher alcohols (B) 1,4-butanediol, and (C) hydroxystyrene derivatives (Fig. S48–S71†). Only experimentally implemented suggestions are shown. The rank and evolution score capture the promising nature of suggestions that were implemented experimentally.

Branched chain higher alcohols,^6^ 1,4-butanediol,^5^ and hydroxystyrene derivatives^48^ serve as model compounds to demonstrate this tool's capability to solve problems in metabolic engineering. Further, these compounds were also selected because they can be synthesized using natural enzymatic transformations we would expect in our knowledge base from RHEA. Here, we show our platform's capability to plan routes, starting from the target compound and ending at a desired host metabolite, by recalling exact reactions and inferring novel transformations not present in our knowledgebase (Fig. 8 and S48–S71†). The ability to infer novel transformations is useful if they have not previously been discovered/evolved or if they are simply missing from the database.

The program is able to propose pathways that require only two non-native steps to shunt intermediates from amino acid biosynthesis pathways to alcohol production (Fig. 8A), similar to the experimentally implemented pathway.^6^ In the first retrosynthetic step, an alcohol dehydrogenase converts the aldehydes into alcohols. In the second retrosynthetic step, a 2-keto-acid decarboxylase converts the 2-keto acids to aldehydes. The proposed 2-keto acids are intermediates in amino acid biosynthesis pathways in *E. coli*, the host. Some suggestions are proposed because an identical reaction is present in the knowledgebase. Others are inferred with ranks ranging from 3–17. Rank 17 suggestion corresponds to the conversion of 2-phenylacetaldehyde to 2-phenylethanol. Several related reactions are missing from our knowledgebase, but they are present in the complete, online version of RHEA (Fig. S72†). Notwithstanding this limitation, the platform is capable of inferring the transformation with a high evolution score.

The program proposes the reductive biosynthesis of 1,4-butanediol starting from α-ketoglutarate and succinyl CoA. The first suggestion for 1,4-butanediol biosynthesis is an alcohol dehydrogenase, which converts the aldehyde 4-hydroxybutyraldehyde (26) into the desired alcohol, 1,4-butanediol (25). Second, a 4-hydroxybutyryl-CoA reductase catalyzes the reduction of 4-hydroxybutyryl CoA (27) to 4-hydroxybutyraldehyde (26). This is a strategy that is automatically learned from the reaction corpus, despite the higher molecular complexity of the reactant over product. Third, a 4-hydroxybutyrl-CoA transferase loads 4-hydroxybutyrate (28) onto coenzyme A. Then, a 4-hydroxybutyrate dehydrogenase converts the aldehyde, succinyl semialdehyde (29), into the alcohol, 4-hydroxybutyrate (28). Finally, succinyl semialdehyde can be synthesized using intermediates from *E. coli*'s (the host) citric acid cycle α-ketoglutarate (30) and succinyl CoA (31) using enzymes 2-oxoglutarate decarboxylase and CoA-dependent succinate semialdehyde dehydrogenase, respectively. This success is particularly impressive considering that there is no high-level retrosynthesis strategy to guide the program. By mimicking the implicit biosynthesis strategy available in the reaction database, the program is able to recover and rank highly the different steps of the experimentally implemented pathway to 1,4-butanediol.^5^

A biosynthetic pathway to synthesize hydroxystyrene derivatives [*e.g.* 4-hydroxy-3-methoxystyrene (32), 3,4-dihydroxystyrene (34), and 4-hydroxystyrene (36)] from l-tyrosine was planned using the tool. The strategy involves the synthesis of key phenolic acid intermediates ferulic acid (33), caffeic acid (35), and 4-coumaric acid (37). Then, the next steps in the proposed pathway use phenolic acid decarboxylases to convert the acids to their styrene derivatives. Using the search parameters suggested in the ESI,† this series of single step retrosynthetic searches took O (1 second) per step (Table S7†). Novel suggestions that were inferred using existing reactions in the database have a high evolution score, capturing the promising nature of experimentally implemented suggestions.

## 3 Discussion

In this study, we developed a tool to facilitate idea generation for single-step enzymatic retrosynthesis. This tool proposes recommendations by generalizing known enzyme chemistry in addition to searching for exact literature precedents. While generalizing, we ensure that our suggestions are conservative for likely experimental feasibility. First, the reaction templates are conserved between the proposed and precedent reactions. Second, the proposed substrates and products are chemically similar to the precedent substrates and products, respectively. Our approach exceeds current methods^18,19^ by carefully handling stereochemistry, while being able to algorithmically extract and apply templates from a database of enzymatic transformations.

To identify suggestions that are experimentally promising, we applied a statistical model that uses an engineered feature representation of pairs of reactions to computationally predict the likelihood of success of enzyme evolution efforts. We first developed two features to capture the substrate and reactive promiscuity of enzymes using molecular and reaction fingerprint similarity, respectively. We then trained the statistical model with pairs of reactions corresponding to co-evolved enzymes and negative examples, totaling ∼30 000 reaction pairs. Next, we applied the resulting model to evaluate the quality of suggestions of experimentally implemented enzymatic synthesis routes to medicinal compounds (*e.g.* Islatravir) and commodity chemicals (*e.g.* 1,4-butanediol). Excitingly, our model performed well and evaluated all relevant suggestions as promising. Further, the absence of exact literature precedents for our recommendations showed that our approach was capable of generalization, thus permitting access to novel transformations. Compared to RetroPath RL, our model has seen more examples of enzyme promiscuity during training/validation (O(10^4^) *vs.* O(10^1^)), and it has also been tested on a larger scale (O(10^3^) *vs.* O(10^2^)). Therefore, through the use of more examples, our approach is better equipped to predict enzyme promiscuity. To our knowledge, this is the first enzyme evolution predictor, integrated into a computer aided synthesis planning tool, capable of generating candidate starting points for evolution campaigns. It sets the stage for the further development of such tools with a deeper understanding of the catalytic mechanisms of enzymes.

Taken together, our retrosynthesis tool and evolution scoring model gave us the ability to perform single-step retrosynthesis. We subsequently expanded our tool's utility by recursively applying the single-step retrosynthesis model to plan the syntheses of medicinal compounds (*e.g.* Islatravir, Molnupiravir) and of commodity chemicals (*e.g.* branched chain higher alcohols, 1,4-butanediol). The algorithm understands and applies the chemical logic associated with multi-step enzymatic pathway design, which would otherwise require the intuition of a trained biochemist. In the final Islatravir synthesis plan, the algorithm started from simple, achiral building blocks and successively built the required stereochemical complexity to yield the target. This synthesis plan was guided by the similarity scoring complemented by SCScore; as a result, the algorithm learned to put together the necessary stereo- and regio-specific enzymatic transformations in the appropriate sequence. Similarly, while planning 1,4-butanediol synthesis, the algorithm successively reduced the oxidized intermediates of the citric acid cycle, succinyl CoA and α-ketoglutarate, to produce 1,4-butanediol. The cofactor NAD(P)H was used to facilitate the reduction reactions, and acetyl-CoA was used as the energy source for the reaction series. Here, by solely using the similarity based ranking to identify how chemically similar products are made, the algorithm took advantage of the implicit biosynthesis strategy available within its extensive enzymatic reaction database, which includes reactions corresponding to ∼22 million enzymes. Therefore, recursive application of single-step enzymatic retrosynthesis model with human intervention is an effective starting point for planning multi-step enzymatic synthesis.

This enzymatic retrosynthesis tool was developed with off-the-shelf algorithm(s) commonly used for organic retrosynthesis to highlight the applicability of existing, organic CASP tools and algorithms to problems in biocatalysis and metabolic engineering. Addressing the following challenges could further advance computer aided planning of syntheses involving enzyme catalyzed reactions.

The reaction dataset includes natural enzymes (*e.g.* metabolic pathways) in a variety of organisms. This imposes a set of challenges. First, the database likely includes enzymes that have never been purified previously, increasing process development risks. Second, enzymes might have to be expressed in yeast, insect or mammalian systems, making process scale up more expensive/challenging. Third, the database has limited examples of enzymes used in synthetic chemistry, limiting the substrate scope of enzymatic transformations. For example, montelukast is an anti-inflammatory medication that uses a ketoreductase to facilitate the reduction of a ketone intermediate to a chiral alcohol.^3^ This opportunity is not captured by the approach presented here because the dataset lacks chemically similar transformations (Fig. S73†).

All reactions in our knowledge base are considered to be reversible. While it is true that biocatalytic cascades can overcome thermodynamic limitations, not all reactions are reversible while also having high yields to make them economically viable. Therefore, incorporating thermodynamic considerations could help avoid some potential low yielding reactions. All reactions are atom mapped computationally,^34^ but since the techniques were developed for organic reactions, some enzymatic transformations could be mapped incorrectly or in an ambiguous fashion. An atom mapping tool tailored to map biochemical transformations, with a set of biochemical heuristics, would enhance the quality of the atom mapping (Fig. S74†).

Our approach inherently favors known chemistries and substrate scopes of enzymes. We identify and rank proposed reactions based on chemical similarity to a precedent reaction. Further, our reaction template is conserved between the proposed and precedent reactions. We emphasize that this is an intentional choice, and our goal is to identify enzymatic synthesis opportunities in a conservative fashion.

Recent studies in protein engineering and *de novo* computational enzyme design have vividly shown the potential of enzymes to catalyze transformations distant from their natural substrate scopes^1^ and to catalyze reactions not previously observed in nature.^15,17^ Novel transformations with no precedents in our knowledge base are not likely to be captured by our approach.

## 4 Conclusion

We have developed a computer aided enzymatic synthesis planner that is based on similarity. In addition to finding exact literature precedents for our recommendations, our retrosynthesis algorithm is also able to generalize enzyme chemistry. An evolution scoring model, that understands ‘similarity’ in the context of enzyme evolution, ensures that the suggestions are feasible and conservative. Through recursive application of the one-step retrosynthesis tool and evolution model, we were able to put together multi-step enzymatic pathways that appropriately capture the chemical logic behind pathway design. The tool's algorithms, models, and dataset have open-access arrangements. Further, proposed reactions are often linked to primary amino acid sequences in UniProt to facilitate experimental implementation of the suggestions. This computer aided synthesis planning tool can aid in brainstorming efforts to develop enzyme-based, sustainable manufacturing processes for commodity chemicals and pharmaceutical agents.

## Data availability

The tool's algorithms, models and datasets are publicly available at https://github.com/karthiksankar93/retrosim_enz. Additional information on the methods and supporting tables and figures are provided in the ESI.†

## Author contributions

Karthik Sankaranarayanan – conceptualization, data curation, formal analysis, investigation, methodology, software, validation, visualization, writing – original draft, writing – review & editing. Esther Heid – data curation, methodology, software, validation, visualization, writing – review & editing. Connor W. Coley – methodology, supervision, writing – review & editing. Deeptak Verma – project administration, software, supervision, writing – review & editing. William H. Green – supervision, writing - review & editing. Klavs F. Jensen – conceptualization, methodology, project administration, resources, supervision, writing – review & editing.

## Conflicts of interest

There are no conflicts to declare.

## Supplementary Material

SC-013-D2SC01588A-s001
