# Supplementary material for: Similarity based enzymatic retrosynthesis
Source: Chem Sci. 2022 Apr 26;13(20):6039–53. doi: 10.1039/d2sc01588a (PMC9132021; doi:10.1039/d2sc01588a)
Supplement: SC-013-D2SC01588A-s001 [file SC-013-D2SC01588A-s001.pdf]

Electronic Supplementary Material (ESI) for Chemical Science.
This journal is © The Royal Society of Chemistry 2022

SUPPORTING INFORMATION

TITLE

Similarity based enzymatic retrosynthesis

AUTHORS

Karthik Sankaranarayanan[a], Esther Heid[a,b], Connor W. Coley[a], Deeptak Verma[c], William. H. Green[a], Klavs F. Jensen[a]
[a] Department of Chemical Engineering, Massachusetts Institute of Technology; 77 Massachusetts Avenue, Cambridge, Massachusetts 02139, United States
[b] Institute of Materials Chemistry, TU Wien, 1060 Vienna, Austria
[c] Computational and Structural Chemistry, Discovery Chemistry, Merck & Co., Inc., Kenilworth, NJ 07033, USA.

Table of Contents

Methods 5
- **Retrosynthesis approach** 5
- **Retrosynthesis algorithm evaluation: top-k accuracy analysis** 7
- **Retrosynthesis algorithm demonstration: product-based search** 9
- **Retrosynthesis algorithm demonstration: recursive multi-step synthesis planning** 10
- **Common biochemical molecule database curation** 12
- **Use of evolution scoring model** 12

Supporting tables and figures 13
- **Figure S1: Retrosynthetic analysis of Islatravir biocatalytic pathway** 13
- **Table S1: Difficulties encountered during atom mapping and resulting number of reactions not atom mapped.** 14
- **Table S2: Reactions containing multiple atom mapping solutions** 14
- **Figure S2: Different fingerprint settings and similarity metrics for one-step retrosynthesis are evaluated using the validation dataset.** 15
- **Figure S3: Example molecule (#1) selected from the one-step retrosynthesis test set.** 16
- **Figure S4: Example molecule (#2) selected from the one-step retrosynthesis test set.** 17
- **Figure S5: Example molecule (#3) selected from the one-step retrosynthesis test set.** 18
- **Figure S6: Example molecule (#4) selected from the one-step retrosynthesis test set.** 19
- **Figure S7: Example molecule (#5) selected from the one-step retrosynthesis test set.** 20
- **Figure S8: Example molecule (#6) selected from the one-step retrosynthesis test set.** 21
- **Figure S9: Example molecule (#7) selected from the one-step retrosynthesis test set.** 22
- **Figure S10: Example molecule (#8) selected from the one-step retrosynthesis test set.** 23
- **Figure S11: Example search (#1) for a target compound already present as a product in the reaction database.** 24
- **Figure S12: Example search (#2) for a target compound already present as a product in the reaction database.** 25
- **Figure S13: Example search (#3) for a target compound already present as a product in the reaction database.** 26

Figure S14: Example search (#4) for a target compound already present as a product in the reaction database. 27
Figure S15: Exemplary molecules in ‘common biochemical molecules’ database. 28
Figure S16: Primary amino acid sequence alignment comparison EMBL-EBI vs. Biopython.............29
Table S3: Uniprot IDs corresponding to amino acid sequences used for EMBL-EBI vs. Biopython comparison and their corresponding % identity score...29
Figure S17: Examples of reaction pairs corresponding to homologous enzymes and negative examples...30
Figure S18: Histogram representing the distribution of % identities of amino acid sequence pairs in the dataset..31
Figure S19: Example (#1) reaction similarity score calculation...32
Figure S20: Example (#2) reaction similarity score calculation...33
Figure S21: Example (#3) reaction similarity score calculation...34
Figure S22: Example (#4) reaction similarity score calculation...35
Table S4: Comparison between different settings (number of hidden layers, number of nodes per hidden layer) in terms of validation ROC-AUC for evolution scoring......................................36
Table S5: Parameters required in neural network for evolution scoring. 36
Figure S23: Network architecture corresponding to multilayer perceptron used to determine probability that reaction pairs are similar. 36
Figure S24: ROC-AUC plot evaluating model performance after training. 36
Figure S25: True positive rate and False positive rate as a function of evolution score threshold. ...37
Figure S26: Example reaction pair (#1) selected from the evolution scoring test set.......................38
Figure S27: Example reaction pair (#2) selected from the evolution scoring test set.......................39
Figure S28: Example reaction pair (#3) selected from the evolution scoring test set.......................40
Figure S29: Example reaction pair (#4) selected from the evolution scoring test set.......................41
Figure S30: Example reaction pair (#5) selected from the evolution scoring test set.......................42
Figure S31: Example reaction pair (#6) selected from the evolution scoring test set.......................43
Figure S32: Example reaction pair (#7) selected from the evolution scoring test set.......................44
Table S6: Reaction- and overall molecular- similarity feature values for the negative examples presented in Figure 6 (5)...45
Figure S33: Reaction details for Figure 6 (5A), a generated negative pair. 46
Figure S34: Reaction details for Figure 6 (5B), a generated negative pair. 47
Figure S35: Reaction details for Figure 6 (5C), a generated negative pair..48
Figure S36: Reaction details for Figure 6 (5D), a generated negative pair. 49
Figure S37: Single step retrosynthetic analysis for Islatravir (Compound 1)....................................50
Figure S38: Single step retrosynthetic analysis for intermediate compound 2 in Islatravir enzymatic synthesis 50
Figure S39: Single step retrosynthetic analysis for intermediate compound 3 in Islatravir enzymatic synthesis. 51
Figure S40: Single step retrosynthetic analysis for intermediate compound 4 in Islatravir enzymatic synthesis. 51
Figure S41: Single step retrosynthetic analysis for intermediate compound 5 in Islatravir enzymatic synthesis. 52
Figure S42: Single step retrosynthetic analysis for intermediate compound 7 in Molnupiravir enzymatic synthesis...52
Figure S43: Single step retrosynthetic analysis for intermediate compound 8 in Molnupiravir enzymatic synthesis...53
Figure S44: Single step retrosynthetic analysis for (13R,17S) -ethyl secol (Compound 10) 53

Figure S45: Single step retrosynthetic analysis for (R)-4-hydroxy isophorone (Compound 11)........54
Figure S46: Single step retrosynthetic analysis for D-tagatose (Compound 12)...............................54
Figure S47: Alternative suggestion to catalyze the selective esterification of the ribose sugar using an isobutyryl donor 55
Figure S48: Single step retrosynthetic analysis for 1-butanol synthesis (Compound 13). 56
Figure S49: Single step retrosynthetic analysis for intermediate compound 14 in 1-butanol synthesis. 56
Figure S50: Single step retrosynthetic analysis for 2-methyl-1-butanol (Compound 15) synthesis. ..57
Figure S51: Single step retrosynthetic analysis for the intermediate compound 16 in 2-methyl-1-butanol synthesis. 57
Figure S52: Single step retrosynthetic analysis for isobutanol synthesis (Compound 17). 58
Figure S53: Single step retrosynthetic analysis for the intermediate compound 18 in isobutanol synthesis. 58
Figure S54: Single step retrosynthetic analysis for 1-propanol synthesis (Compound 19). 59
Figure S55: Single step retrosynthetic analysis for the intermediate compound 20 in 1-propanol synthesis. 59
Figure S56: Single step retrosynthetic analysis for 2-phenylethanol synthesis (Compound 21)........60
Figure S57: Single step retrosynthetic analysis for the intermediate compound 22 in 2-phenylethanol synthesis...60
Figure S58: Single step retrosynthetic analysis for isobutanol synthesis (Compound 23). 61
Figure S59: Single step retrosynthetic analysis for the intermediate compound 24 in isobutanol synthesis. 61
Figure S60: Single step retrosynthetic analysis for 1,4-butanediol (Compound 25). 62
Figure S61: Single step retrosynthetic analysis for the intermediate compound 26 in 1,4-butanediol synthesis. 62
Figure S62: Single step retrosynthetic analysis for the intermediate compound 27 in 1,4-butanediol synthesis. 63
Figure S63: Single step retrosynthetic analysis for the intermediate compound 28 in 1,4-butanediol synthesis. 63
Figure S64: Single step retrosynthetic analysis for the intermediate compound 29 in 1,4-butanediol synthesis. 64
Figure S65: Single step retrosynthetic analysis for the intermediate compound 29 in 1,4-butanediol synthesis. 64
Figure S66: Single step retrosynthetic analysis for 4-hydroxy-3-methoxystyrene (Compound 32). ..65
Figure S67: Single step retrosynthetic analysis for the intermediate compound 33 in hydroxystyrene derivative synthesis..65
Figure S68: Single step retrosynthetic analysis for 3,4-dihydroxy styrene (Compound 34)..............66
Figure S69: Single step retrosynthetic analysis for the intermediate compound 35 in hydroxystyrene derivative synthesis..66
Figure S70: Single step retrosynthetic analysis for 4-hydroxystyrene (Compound 36). 67
Figure S71: Single step retrosynthetic analysis for the intermediate compound 37 in hydroxystyrene derivative synthesis..67
Figure S72: RHEA reactions 63736 and 63740 are missing from our knowledgebase. 68
Table S7: The single-step retrosynthetic search for hydroxstyrene derivatives takes *O* (1 second) per step. 69
Figure S73: Poor applicability of the tool to enzymes outside the scope of current data set. 70
Figure S74: Ambiguous atom mapper output for an exemplary enzymatic transformation. 71

Figure S75: Chemical similarity is evaluated using RDKit's implementation of Morgan circular fingerprint (radius =2, using chirality and features) and Dice similarity. 72
Table S8: Reaction processing to ensure compatibility with RDChiral. 72
Table S9: RDChiral shows promise for applications in enzymatic retrosynthesis. 73
Figure S76: Additional features in RDEnzyme relevant to enzymatic retrosynthesis. 74
Table S10: RDEnzyme is evaluated to determine compatibility with RHEA enzymatic dataset. 75

References: 76

Methods

Retrosynthesis approach

First, we recall similar products from the enzymatic reaction database (Figure 2, step 1). Quantifying chemical similarity based on two-dimensional structure requires a fingerprinting technique and a similarity metric. This study explores a range of fingerprint settings and similarity metrics. However, we emphasize our goal is not to conduct an exhaustive search of parameters, but rather to identify settings that facilitate computational enzymatic retrosynthesis.

Following procedures adapted from Coley et. al.,[1] Morgan circular fingerprints were selected to represent chemical molecules (Figure S75). In RDKit's implementation, 'radius', 'features', and 'chirality' are three user specified parameters for defining Morgan fingerprints. The 'radius' of a circular fingerprint refers to the size of the largest neighborhood surrounding each atom that is considered during enumeration. Fingerprinting 'with chirality' refers to the inclusion of stereochemistry of the atom in consideration. Fingerprinting 'with features' refers to the inclusion of information in the initial atom encoding beyond atomic identity to, for example, take into consideration the similarity between different halogens. We chose to evaluate Morgan fingerprints of radius = 2,3 and with/without features in this study. Chirality was always set to true in order to capture stereo-specificity of enzymatic transformations.

As in Coley et al.[1], Dice , Tanimoto , Tversky similarity metrics were evaluated in this study. Dice similarity (Eq. 1) quantifies the similarity between two fingerprint vectors **x** and **y** by calculating the ratio between the prevalence of overlapping substructures (as measured by nonzero values of $x_i y_i$ for each vector index i) and the number of distinct substructures observed in each (as measured by the summation over x_i^2 and y_i^2 for each fingerprint separately). The Tanimoto metric (Eq. 2) instead normalizes the prevalence of overlapping substructures (in x and y) by the total number of unique substructures (in x or y). The Tversky similarity (Eq. 3) is a generalization of the Tanimoto similarity that is parametrized by α and β to enable an asymmetrically weighted normalization.

$$Dice\ (\boldsymbol{x}, \boldsymbol{y}) = \frac{2\sum x_i y_i}{\sum x_i{}^2 + \sum y_i{}^2}\ (Eq\ 1)$$

$$Tanimoto\ (\boldsymbol{x}, \boldsymbol{y}) = \frac{\sum x_i y_i}{\sum x_i{}^2 + \sum y_i{}^2 - \sum x_i y_i}\ (Eq\ 2)$$

$$Tversky\ (\boldsymbol{x}, \boldsymbol{y}; \boldsymbol{\alpha}, \boldsymbol{\beta}) = \frac{\sum x_i y_i}{\boldsymbol{\alpha}\sum x_i{}^2 + \boldsymbol{\beta}\sum y_i{}^2 - \sum x_i y_i}\ (Eq\ 3)$$

Second, enzymatic retrosynthetic analysis requires a tool capable of extracting and applying reaction templates, i.e., subgraph patterns that describe the changes in connectivity between a product molecule and its corresponding reactant(s) (Figure 2, Step 2). RDChiral is an open-source Python wrapper for RDKit designed to accomplish this task for organic transformations[2]. First, its retrosynthetic template extraction algorithm generates SMARTS patterns from atom-mapped reaction SMILES strings. Second, its template application algorithm generates reaction precursors from products while consistently handling stereochemical information.

Our atom mapped dataset was processed to ensure compatibility with the RDChiral[1,2]. First, multi-product reactions were enumerated into multiple reactions, each containing a single product.

Then, reactions resulting in by-product formation (or salts) were removed. A by-product or salt was defined as a reaction product that (1) appeared at least 50 times in the dataset or (2) had a SMILES string of length less than two characters. Finally, transport reactions (e.g. proteins facilitating transport of molecules across a membrane) were discarded by identifying reactions where the reactant and product smiles were identical. (Table S8)

RDChiral, initially designed for organic transformations, also showed promise for applications in enzymatic retrosynthesis, but was only able to capture 71.3% of the reactions RHEA enzymatic reaction database[3] as opposed to 97.4% USPTO dataset[1] (Table S9). As a result, we built upon RDChiral to develop RDEnzyme for template extraction and application of enzymatic transformations (Figure S76). First, RDEnzyme treats stereochemistry associated with leaving groups consistently. Second, it is capable of handling stereo-isomerization reactions. Third, it allows for the destruction of chiral centers away from the reaction center in proposed retrosynthetic transformations, even if not indicated in the precedent transformation. This change reflects the potential to use directed evolution to change the stereo-specificity of enzyme catalyzed reactions[4].

We evaluated RDEnzyme on the RHEA enzymatic dataset. First, for every reaction, a generalized retrosynthetic template was extracted. Second, this template was applied to the product of the reaction to generate a set of proposed reactants. Because of the generalized nature of the template, multiple sets of reactants were possible during this step. Finally, the presence of the true reactant in the set of proposed reactants was verified by requiring a canonicalized SMILES string match. When multiple atom mapping solutions existed for a given reaction (~7% of dataset), the same procedure was applied to all atom mapped solutions. If any atom mapped solution met the success criteria, then the overall reaction was considered a success. This strategy was selected since in many cases only one of the atom mapped solution was accurate. RDEnzyme was able to extract templates successfully for 90.6% of the reactions in the RHEA enzymatic dataset, in contrast to the 71.3 % extracted by RDChiral (Table S10).

Only the successful transformations were used for the retrosynthetic analysis and associated case studies. As a consequence, our algorithm was able to extract and apply a retrosynthetic template to yield stereochemically accurate reactants for all reactions in the published dataset.

Finally, the proposed reactions are ranked by overall molecular similarity scores, defined as ($\text{similarity}_{\text{reactant}}$*$\text{similarity}_{\text{product}}$) (Figure 2, step 3).

Retrosynthesis algorithm evaluation: top-k accuracy analysis

Here, we restate our approach to performing the top-k accuracy analysis in greater detail.

Dataset Processing:

1. Processed data from RHEA contained 15690 reactions. 142 reactions containing single atom products are removed. At the end of this step, 15,548 total reactions remained.
2. Reactions containing multiple atom mapped solutions were enumerated to ensure that every solution is considered during the retrosynthetic analysis. At the end of this step, the dataset contained 16,648 total reactions.
3. Duplicate reactions, even if they correspond to different enzymes, were filtered out to ensure only one instance of every reaction is present. Briefly, reaction SMILES were canonicalized after removing atom mapping information. This canonicalized reaction SMILES string was used to identify duplicates. At the end of this step, 14,013 total reactions remained.
4. Transformation rules from the resulting reactions were extracted using RDEnzyme. In summary, the transformation rules contain atoms and bonds that changed in the course of the reaction, and a varying number of neighbors determined using a fixed distance and/or heuristics that decide which neighboring atoms are relevant. Reactions corresponding to rare templates were filtered out, and only reactions with rules that occurred at least three times were kept for this analysis. At the end of this step, 6973 total reactions remained.
5. The dataset was randomly split into training: validation: test splits, 5,578 (80%): 697 (10%): 698 (10%).

Retrosynthesis Approach:

1. Calculate the Morgan fingerprint (radius =2, using chirality and features) of the target compound.
2. Calculate a product Dice similarity score, s_{prod}, between the target compound and each product that appears in the training set.
3. Iterate through each of the precedent reactions from the knowledge base in order of decreasing product similarity. For computational efficiency, this considers the 40 most similar products only. For each of these reaction precedents, extract a localized reaction template based on the atom mapped transformation, using RDEnzyme. In the control experiment, a random set of 40 products from the training set were considered.
4. Still iterating through the precedent reactions, apply the extracted template to the target molecule to get candidate precursors.
5. For each candidate precursor generated in the previous step, compute the candidate precursor's Morgan fingerprint. Then, compare it to the reaction precedent's reactants' fingerprint to get a second similarity score, s_{reac}. This score reflects how similar the reactants of the known reaction are to the proposed reactants of this theoretical reaction.
6. Still for each candidate precursor set, multiply the product similarity score s_{prod} with the reactant similarity score s_{reac} to get the overall similarity $s = s_{prod} \cdot s_{reac}$. This score represents the extent to which the proposed retrosynthetic disconnection is analogous to the precedent reaction.
7. Rank all candidate disconnections by their overall scores, s. Remove any duplicates in the candidate precursor list as determined by their isomeric SMILES string, while retaining only the highest score when there are multiple entries. In the control experiment, candidate disconnections were ranked randomly.

8. Compare each of the candidate precursor's isomeric SMILES representation to the target compound's known reactant SMILES string to evaluate the performance of the approach.

Random control experiments were run in triplicate. Mean and standard deviation of the three independent runs are reported.

Retrosynthesis algorithm demonstration: product-based search

Here, we restate our approach to identifying and prioritizing different transformations resulting in the synthesis of an identical product. The approach is similar to the retrosynthesis algorithm used for the top-k accuracy analysis; we restate it in greater detail for clarity.

Dataset Processing:

1. Processed data from RHEA contained 15690 reactions. 142 reactions containing single atom products are removed. At the end of this step, 15,548 total reactions remained.
2. If multiple atom mapped solutions were present for a given enzymatic transformation, one was selected at random. Because this analysis was performed manually, the experimenter could explore alternative atom mapped solutions for relevant reactions without algorithmically enumerating all different possibilities resulting from atom-mapping ambiguity.

Retrosynthesis approach:

1. Calculate the Morgan fingerprint (radius =2, using chirality and features) of the target compound.
2. Calculate a product Dice similarity score, s_{prod}, between the target compound and each product that appears in the training set.
3. Iterate through each of the precedent reactions from the knowledge based in order of decreasing product similarity. For computational efficiency and ease of manual analysis, this considers the 40 most similar products only. For each of these reaction precedents, extract a localized reaction template based on the atom mapped transformation, using RDEnzyme.
4. Still iterating through the precedent reactions, apply the extracted template to the target molecule to get candidate precursors.
5. For each candidate precursor generated in the previous step, compute the candidate precursor's Morgan fingerprint. Then, compare it to the reaction precedent's reactants' fingerprint to get a second similarity score, s_{reac}. This score reflects how similar the reactants of the known reaction are to the proposed reactants of this theoretical reaction.
6. Still for each candidate precursor set, multiply the product similarity score s_{prod} with the reactant similarity score s_{reac} to get the overall similarity $s = s_{prod} \cdot s_{reac}$. This score represents the extent to which the proposed retrosynthetic disconnection is analogous to the precedent reaction.
7. Rank all candidate disconnections by their overall scores, s. Remove any duplicates in the candidate precursor list as determined by their isomeric SMILES string, while retaining only the highest score when there are multiple entries.

Retrosynthesis algorithm demonstration: recursive multi-step synthesis planning

Here, we restate our approach to recursive multi-step synthesis planning for test molecules (Islatravir, Molnupiravir, (13R, 17S)-ethyl secol, (R)-4-hydroxy isophorone, D-tagatose ,branched chain higher alcohols, 1,4-butanediol, and hydroxystyrene derivatives). The approach is similar to the retrosynthesis algorithm used for the top-k accuracy analysis; we restate it in greater detail for clarity.

Dataset Processing:

1. Processed data from RHEA contained 15690 reactions. 142 reactions containing single atom products are removed. At the end of this step, 15,548 total reactions remained.
2. Reactions containing multiple atom mapped solutions were enumerated to ensure that every solution is considered during the retrosynthetic analysis.

Retrosynthesis approach:

1. Calculate the Morgan fingerprint (radius =2, using chirality and features) of the target compound.
2. Calculate a product Dice similarity score, s_{prod}, between the target compound and each product that appears in the training set.
3. Iterate through each of the precedent reactions from the knowledge based in order of decreasing product similarity. For computational efficiency and ease of manual analysis, this considers the 50 most similar products only. For each of these reaction precedents, extract a localized reaction template based on the atom mapped transformation, using RDEnzyme.
4. Still iterating through the precedent reactions, apply the extracted template to the target molecule to get candidate precursors.
5. For each candidate precursor generated in the previous step, compute the candidate precursor's Morgan fingerprint. Then, compare it to the reaction precedent's reactants' fingerprint to get a second similarity score, s_{reac}. This score reflects how similar the reactants of the known reaction are to the proposed reactants of this theoretical reaction.
6. Still for each candidate precursor set, multiply the product similarity score s_{prod} with the reactant similarity score s_{reac} to get the overall similarity $s = s_{prod} \cdot s_{reac}$. This score represents the extent to which the proposed retrosynthetic disconnection is analogous to the precedent reaction.
7. Rank all candidate disconnections by their overall scores, *s*. Remove any duplicates in the candidate precursor list as determined by their isomeric SMILES string, while retaining only the highest score when there are multiple entries.

SCScore based ranking for enzyme use in biocatalysis applications:

1. Compute the SCScore of the product, defined as $SCScore_{product.}$
2. Consider all or the top-50 candidate disconnections (whichever is lower) ranked by overall similarity score, *s*.
3. For each candidate disconnection, iterate through the proposed reactants to check if they are present in a buyable database. For this publication, we checked the 'common biochemical molecule' database (see S.I. for curation details) and the commercial buyable database available in ASKCOS[5]. If a reactant is present in the buyable database, its SCScore was set to 1.

4. Still iterating through the reactants, compute the individual SCScores of reactants that are not buyable.
5. The maximum SCScore amongst all reactants is defined as $SCScore_{reactant}$.
6. Still for each candidate disconnection, compute $\Delta SCScore$ defined as $SCScore_{product}$ - $SCScore_{reactant}$.
7. Rank all candidate disconnections by $\Delta SCScore$.

We note that 2-phenylethanol (compound **21**) required the expansion of the search space to 500 similar products (step 3, retrosynthesis approach) in order to find the experimentally implemented solution.

Common biochemical molecule database curation

Commonly occurring biological molecules (cofactors, atp, etc.) are challenging to synthesize chemically using commercially available building blocks. However, they are often readily purchasable through commercial vendors. Therefore, we curate a set of biochemical molecules that are likely to be commercially available.

The enzymatic reaction dataset is analyzed to identify molecules that occur as products greater than 50 times. We identify a set of 51 such molecules, which are collectively present, as products, greater than 18,000 times in the dataset. We label these molecules as ‘common biochemical molecules’. A selection of these molecules is available in Figure S15.

Use of evolution scoring model

The evolution scoring model was trained using complete, balanced chemical reactions. Therefore, when using this model, it is important to provide as input complete, balanced chemical reactions. Input examples are available in Figure 6.

Supporting tables and figures

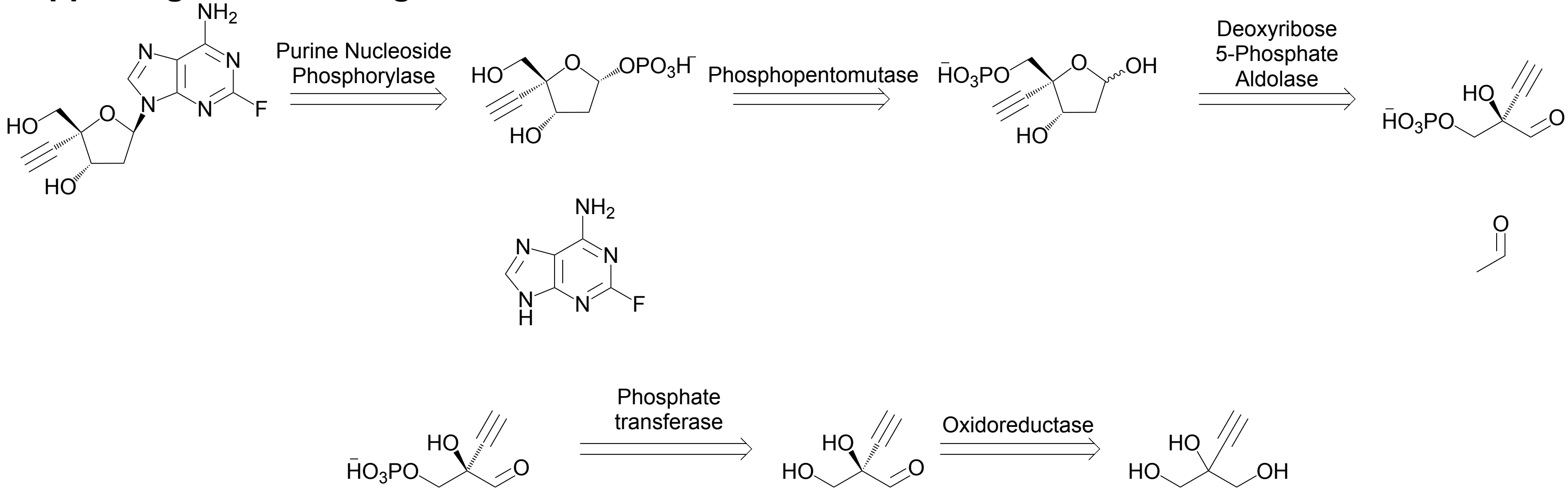

Figure S1: Retrosynthetic analysis of Islatravir biocatalytic pathway

Table S1: Difficulties encountered during atom mapping and resulting number of reactions not atom mapped.

Processing Step	Reactions Lost	Reactions Available
RHEA enzymatic reaction files (ChemDraw)		24,546
RDKit conversion from ChemDraw File to reaction SMILES	57	24,489
Reactions with wildcard (lost)	5084	19,405
Reactions with atom mapping timeout error (lost) (t=1000s/reaction)	659	18,746
Reaction lost due to miscellaneous reasons	682	18064

Table S2: Reactions containing multiple atom mapping solutions

Number of solutions	Number of reactions
1	16867
2	978
3	199
4	18
5	2

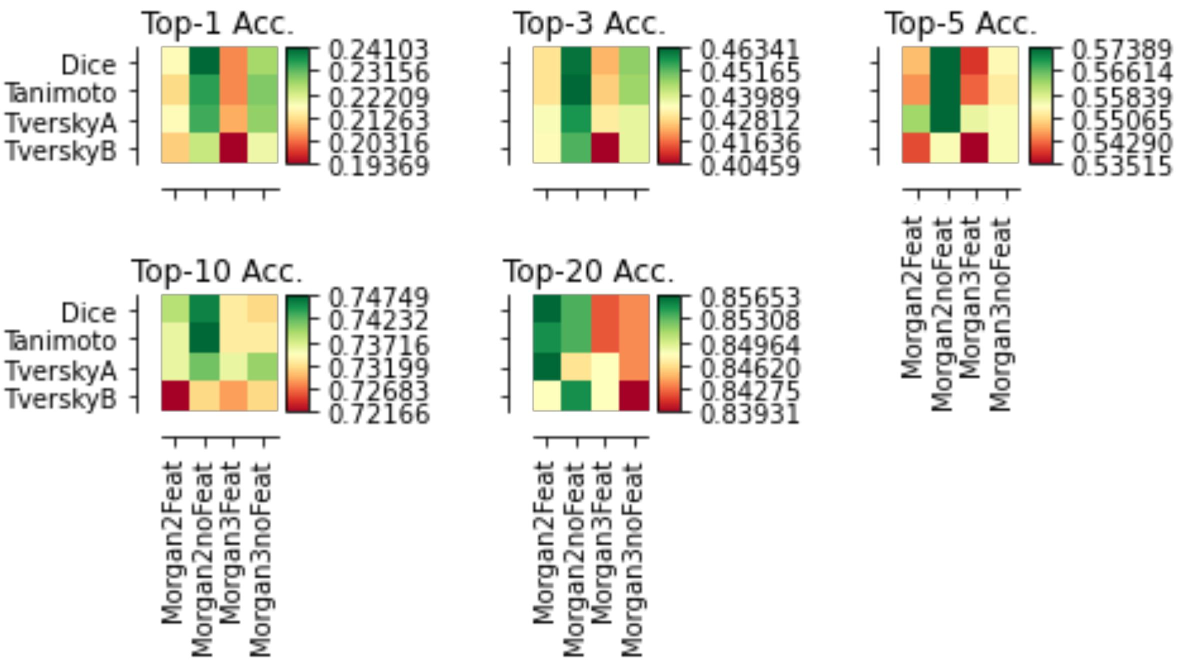

Figure S2: Different fingerprint settings and similarity metrics for one-step retrosynthesis are evaluated using the validation dataset. 'TverskyA' and 'TverskyB' are Tversky similarity metrics with (α=1.5, β=1.0) and (α=1.0, β=1.5), respectively (see equation 3). 'Morgan2Feat' and 'Morgan2noFeat' refer to Morgan fingerprints of radius =2 with and without features, respectively. 'Morgan3Feat' and 'Morgan3noFeat' refer to Morgan fingerprints of radius =3 with and without features, respectively.

Recorded Reaction (RHEA:24210)

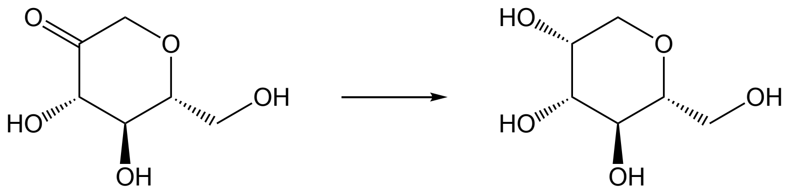

A selection of recommended precursors

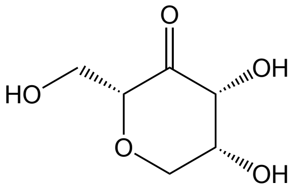

0.74

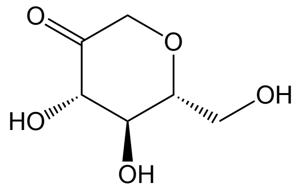

0.68 [True]

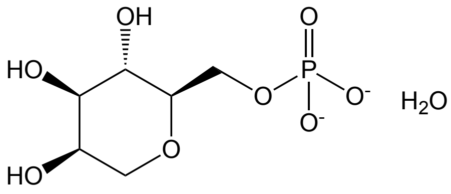

0.64

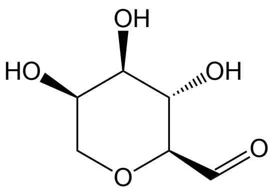

0.40

Figure S3: Example molecule (#1) selected from the one-step retrosynthesis test set. The developed algorithm was used as a brainstorming tool, and a subset comprising promising suggestions was selected. Numbers below precursor suggestions correspond to overall molecular similarity score. The approach proposes the recorded precursors with Rank 3 using a ketoreductase. Other suggestions include reductases (with different regio- or chemo- selectivity), and hydrolase.

Recorded reaction (RHEA:25798)

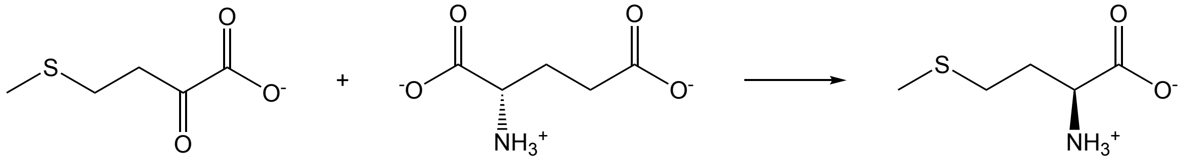

A selection of recommended precursors

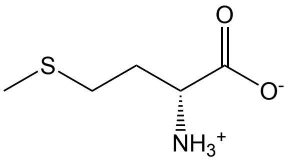

1.0

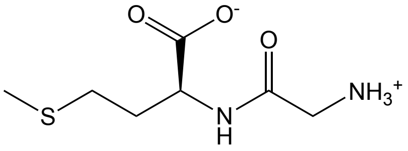

1.0

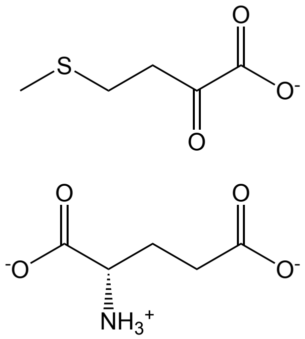

0.61 [True]

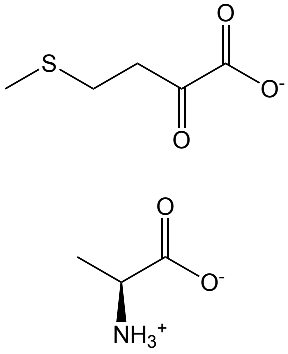

0.57

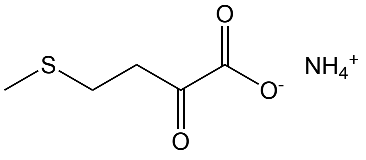

0.57

Figure S4: Example molecule (#2) selected from the one-step retrosynthesis test set. The developed algorithm was used as a brainstorming tool, and a subset comprising promising suggestions was selected. Numbers below precursor suggestions correspond to overall molecular similarity score. The approach proposes the recorded precursors with Rank 10 using a transaminase. Other suggestions include racemase, dipeptidase, transaminase (with a different amine donor), and dehydrogenase.

Recorded reaction (RHEA:24285)

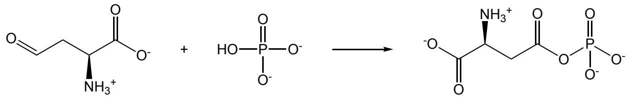

A selection of recommended precursors

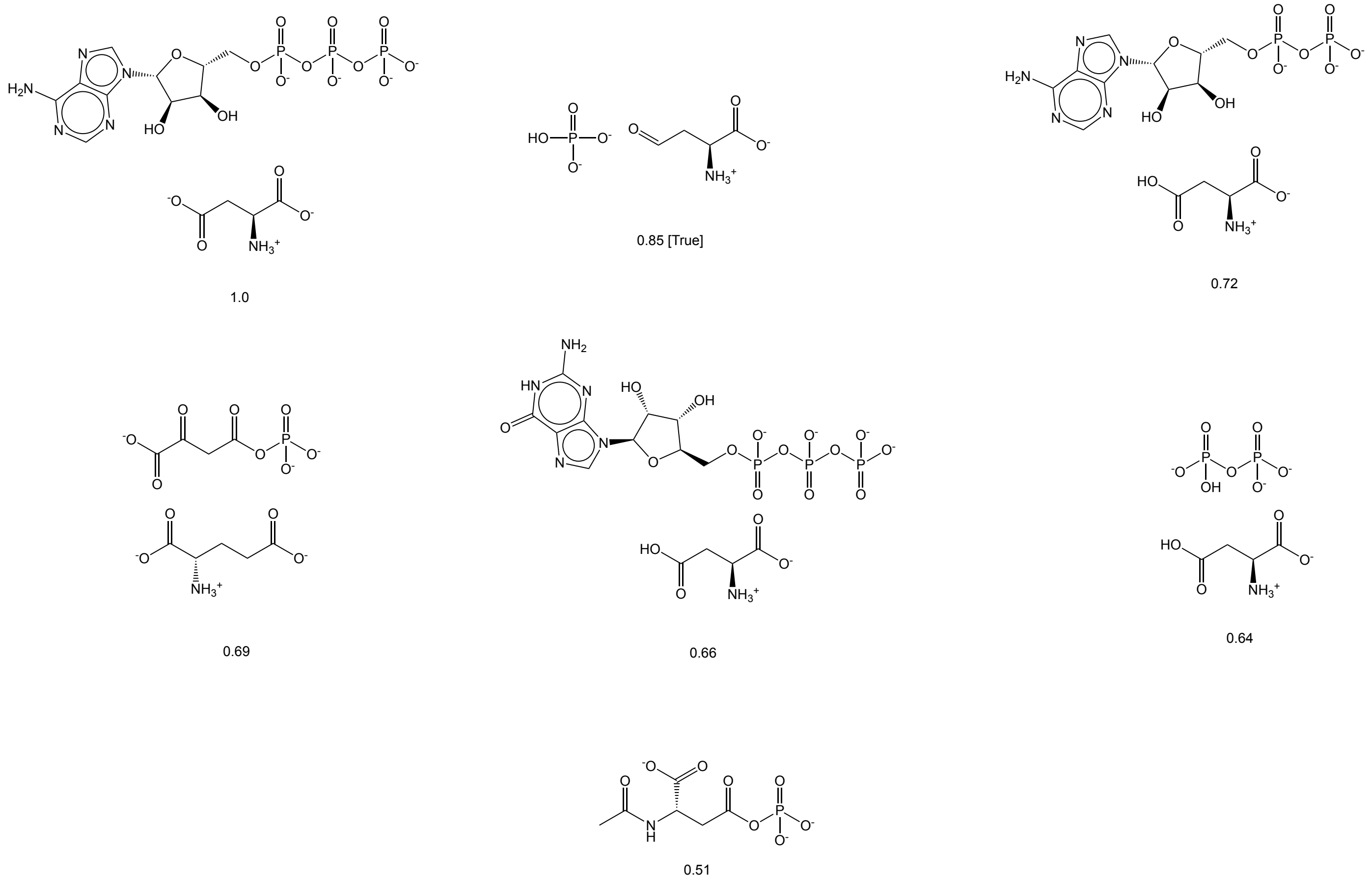

Figure S5: Example molecule (#3) selected from the one-step retrosynthesis test set. The developed algorithm was used as a brainstorming tool, and a subset comprising promising suggestions was selected. Numbers below precursor suggestions correspond to overall molecular similarity score. The approach proposes the recorded precursors with Rank 2 using a phosphate dependent semi-aldehyde dehydrogenase. Other suggestions include kinases with different phosphate donors (e.g. ATP, ADP, GTP), diphosphate dependent phosphotransferase, transaminase, and deacetylase.

Recorded reaction (RHEA:34264)

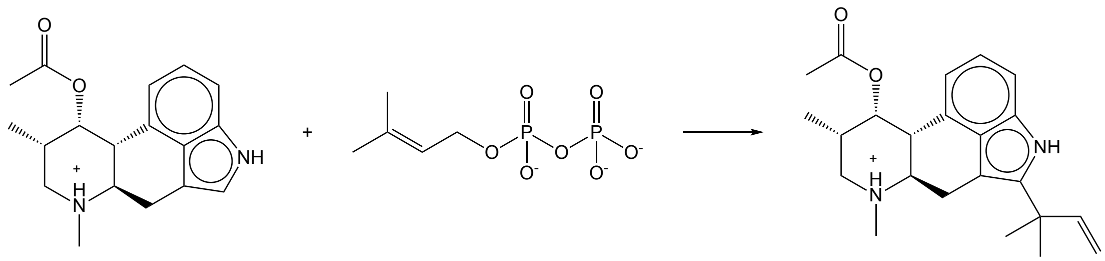

A selection of recommended precursors

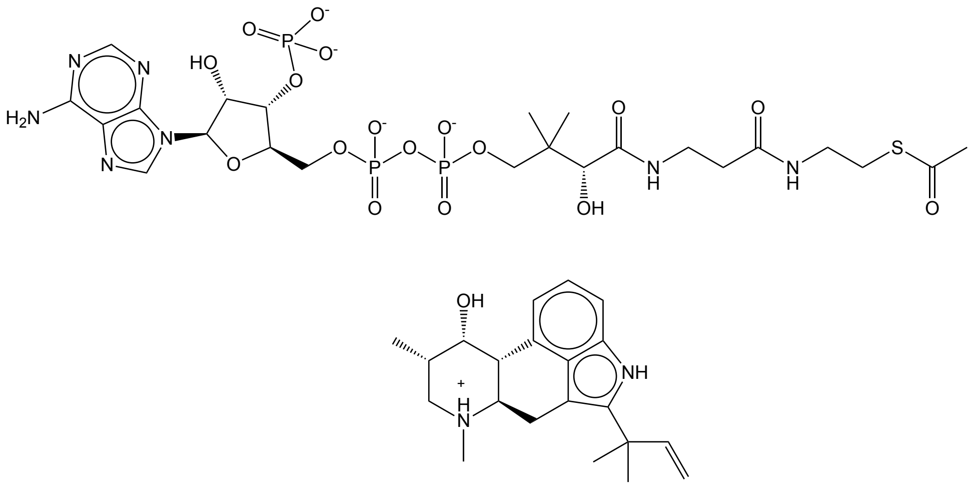

0.56

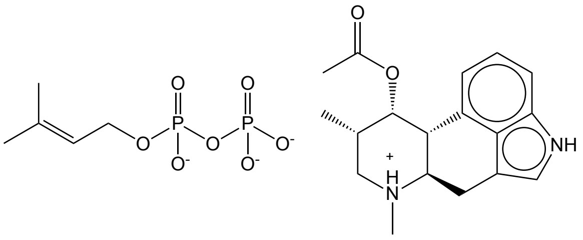

0.49 [True]

Figure S6: Example molecule (#4) selected from the one-step retrosynthesis test set. The developed algorithm was used as a brainstorming tool, and a subset comprising promising suggestions was selected. Numbers below precursor suggestions correspond to overall molecular similarity score. The approach proposes the recorded precursors with Rank 2 using a prenyltransferase. O-acetyltransferase is proposed as an alternative suggestion.

Recorded reaction (RHEA:28243)

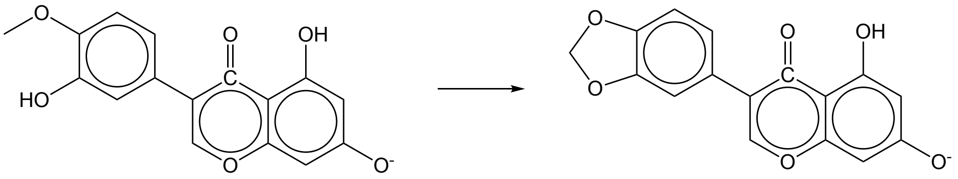

A selection of recommended precursors

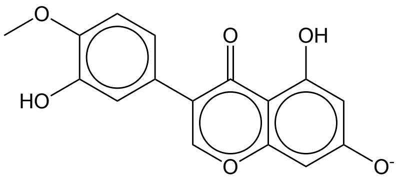

0.84 [True]

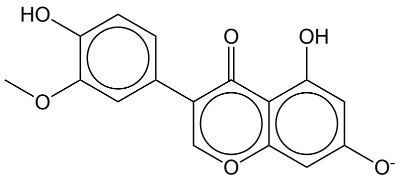

0.81

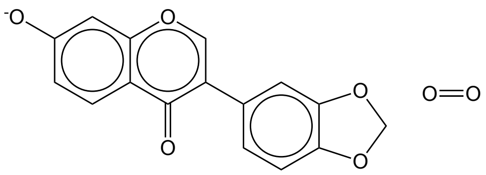

0.71

Figure S7: Example molecule (#5) selected from the one-step retrosynthesis test set. The developed algorithm was used as a brainstorming tool, and a subset comprising promising suggestions was selected. Numbers below precursor suggestions correspond to overall molecular similarity score. The approach proposes the recorded precursors with Rank 1 using a Cytochrome P450 to catalyze the methylenedioxy bridge formation reaction. Cytochrome P450 catalyzed hydroxylation is proposed as an alternative reaction.

Recorded reaction (RHEA:59301)

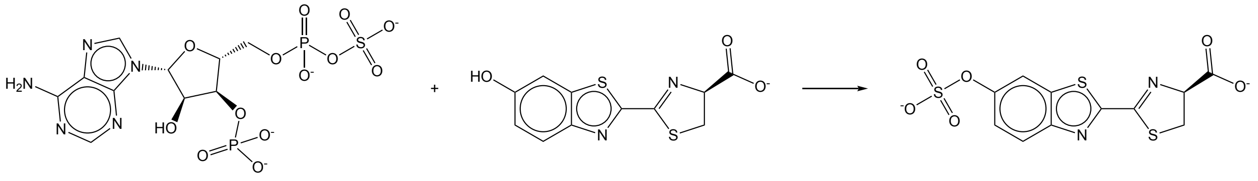

A selection of recommended precursors

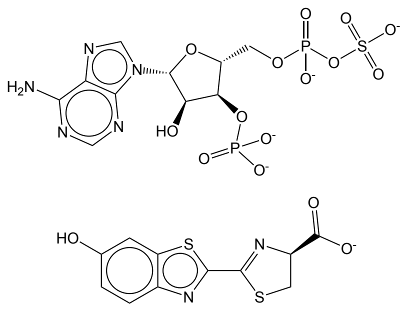

0.88 [True]

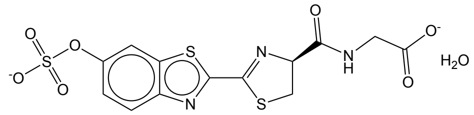

0.24

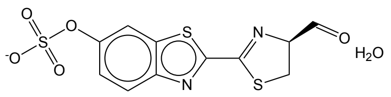

0.18

Figure S8: Example molecule (#6) selected from the one-step retrosynthesis test set. The developed algorithm was used as a brainstorming tool, and a subset comprising promising suggestions was selected. Numbers below precursor suggestions correspond to overall molecular similarity score. The approach proposes the recorded precursors with Rank 1 using a sulfotransferase. Other suggestions include hydrolase and oxidoreductase.

Recorded reaction (RHEA:36993)

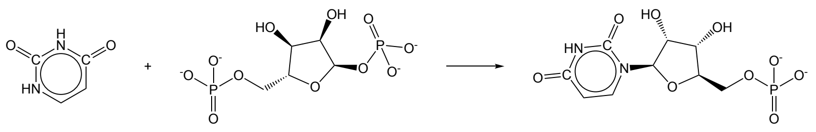

A selection of recommended precursors

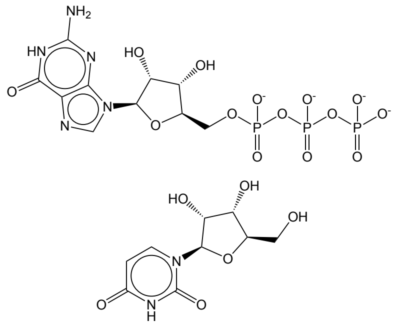

0.97

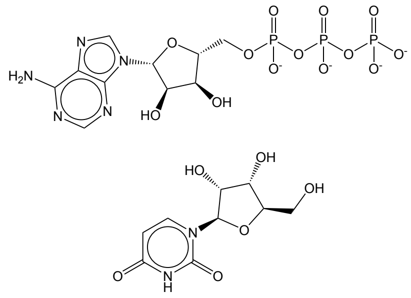

0.97

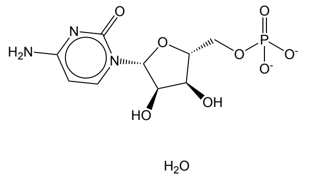

0.76

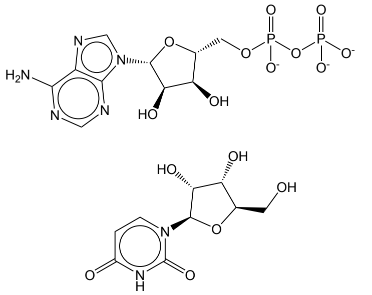

0.72

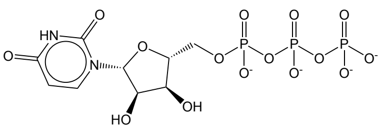

0.67

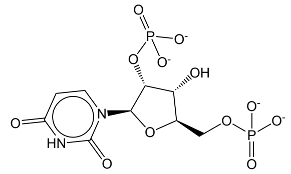

0.66

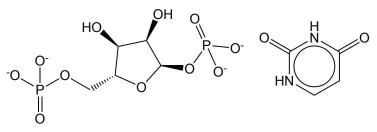

0.60 [True]

Figure S9: Example molecule (#7) selected from the one-step retrosynthesis test set. The developed algorithm was used as a brainstorming tool, and a subset comprising promising suggestions was selected. Numbers below precursor suggestions correspond to overall molecular similarity score. The approach proposes the recorded precursors with Rank 11 using a phosphorylase to catalyze the displacement of a phosphate group with uracil. Other suggestions include phosphate transferases (with different phosphate donors including GTP, ATP, and ADP), deaminase, nucleotide diphosphatase, and phosphatase.

Recorded reaction (RHEA:56529)

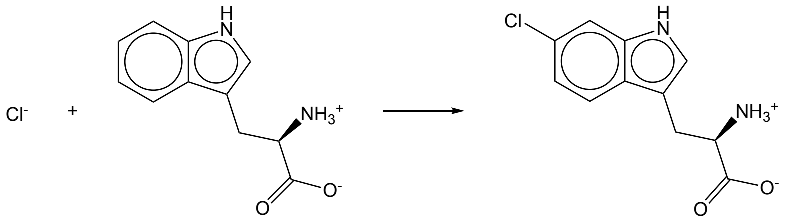

A selection of recommended precursors

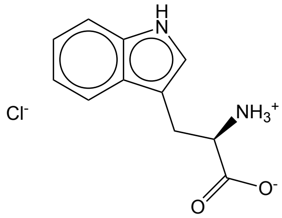

0.82
[True]

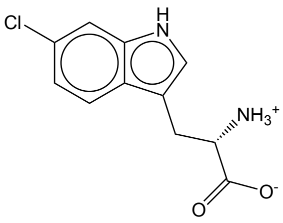

0.78

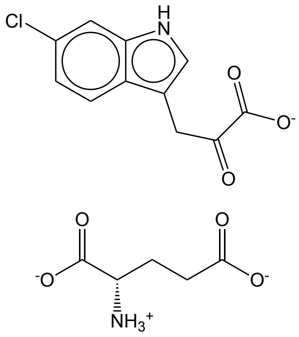

0.73

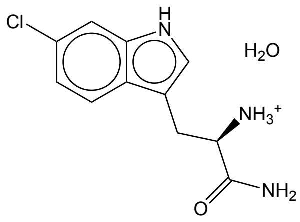

0.70

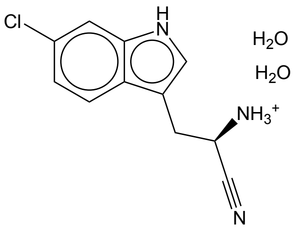

0.55

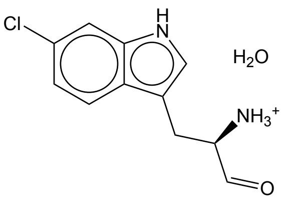

0.54

Figure S10: Example molecule (#8) selected from the one-step retrosynthesis test set. The developed algorithm was used as a brainstorming tool, and a subset comprising promising suggestions was selected. Numbers below precursor suggestions correspond to overall molecular similarity score. The approach proposes the recorded precursors with Rank 1 using a halogenase. Other suggestions include racemase, transaminase, amidase, nitrilase, and oxidoreductase.

Target compound:

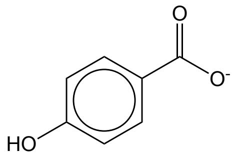

A selection of recommended precursors

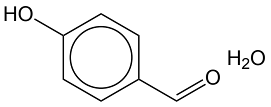

1.0 [True]

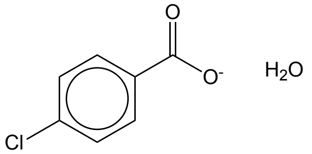

1.0 [True]

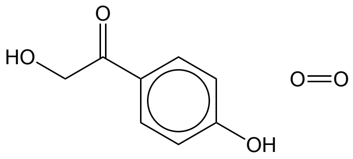

1.0 [True]

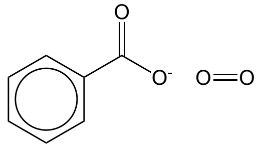

1.0 [True]

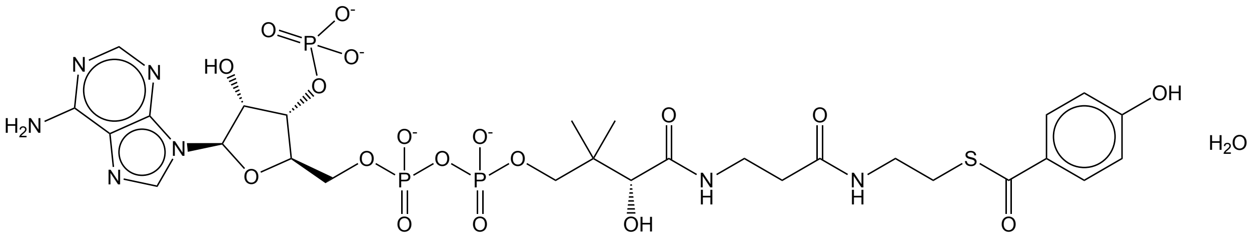

1.0 [True]

Figure S11: Example search (#1) for a target compound already present as a product in the reaction database. The developed algorithm was used as a brainstorming tool, and a subset comprising promising suggestions was selected. Multiple known approaches to synthesize the target compound are shown along with their overall molecular similarity score. Proposed enzymes include 4-hydroxybenzaldehyde dehydrogenase, 4-chlorobenzoate dehalogenase, 2,4′-dihydroxyacetophenone dioxygenase, benzoate-para-hydroxylase, and 4-hydroxybenzoyl-CoA thioesterase

Target compound:

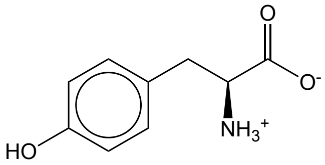

A selection of recommended precursors

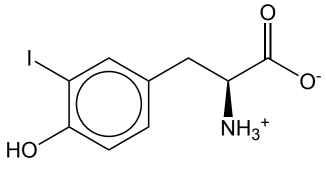

1.0 [True]

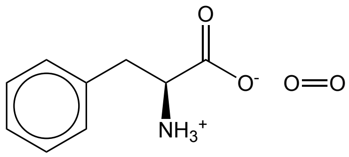

1.0 [True]

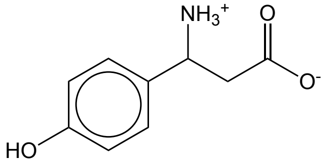

1.0 [True]

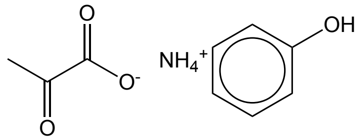

1.0 [True]

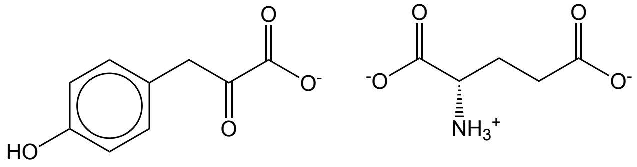

1.0 [True]

Figure S12: Example search (#2) for a target compound already present as a product in the reaction database. The developed algorithm was used as a brainstorming tool, and a subset comprising promising suggestions was selected. Multiple known approaches to synthesize the target compound are shown along with their overall molecular similarity score. Proposed enzymes include iodotyrosine dehalogenase, phenylalanine 4-monooxygenase, tyrosine 2,3-aminomutase, tyrosine phenol-lyase and aspartate transaminase.

Target compound:

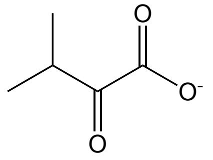

A selection of recommended precursors

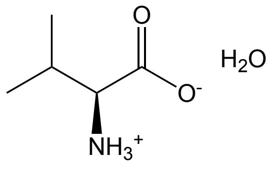

1.0 [True]

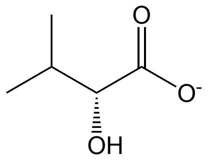

1.0 [True]

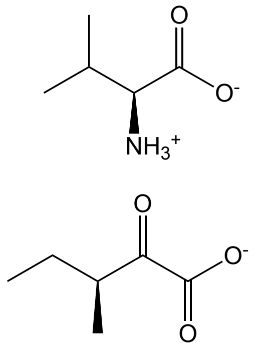

1.0 [True]

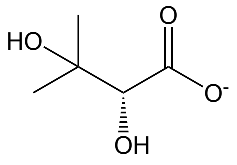

1.0 [True]

Figure S13: Example search (#3) for a target compound already present as a product in the reaction database. The developed algorithm was used as a brainstorming tool, and a subset comprising promising suggestions was selected. Multiple known approaches to synthesize the target compound are shown along with their overall molecular similarity score. Proposed enzymes include valine dehydrogenase, D-hydroxyisovalerate dehydrogenase, valine-isoleucine transaminase, and dihydroxy-acid dehydratase.

Target compound:

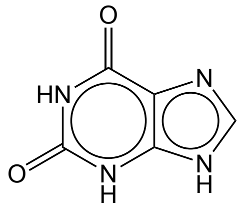

A selection of recommended precursors

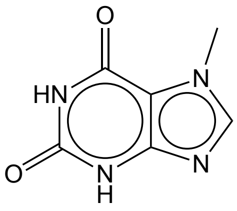

1.0 [True]

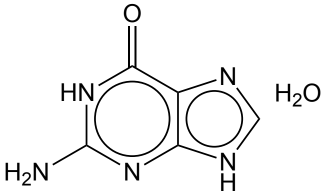

1.0 [True]

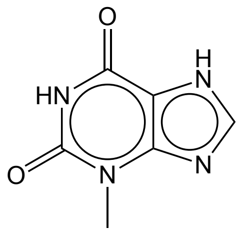

1.0 [True]

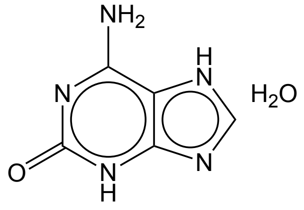

1.0 [True]

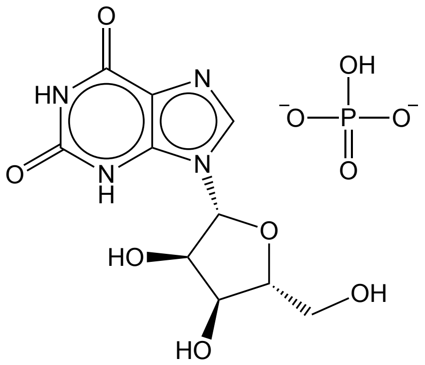

1.0 [True]

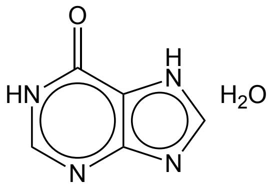

1.0 [True]

Figure S14: Example search (#4) for a target compound already present as a product in the reaction database. The developed algorithm was used as a brainstorming tool, and a subset comprising promising suggestions was selected. Multiple known approaches to synthesize the target compound are shown along with their overall molecular similarity score. Proposed enzymes include N-demethylases, deaminases, phosphorylase, and xanthine dehydrogenase.

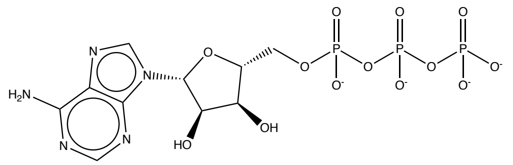

Adenosinetriphosphate (ATP)

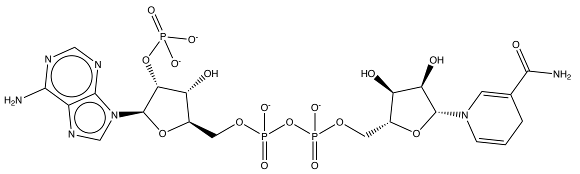

Reduced Nicotinamide Adenine Dinucleotide Phosphate (NADPH)

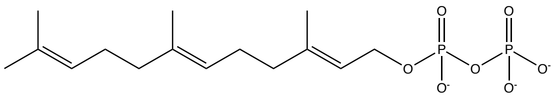

E-E-farnesyl diphosphate

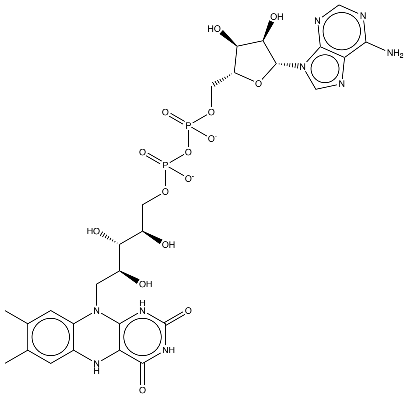

FADH2 dianion

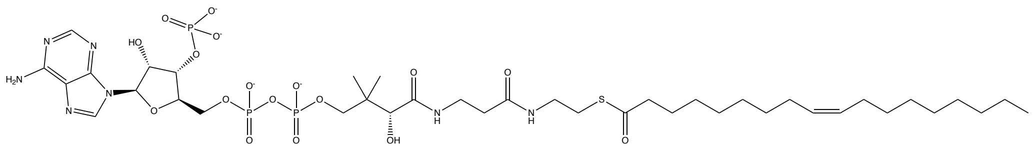

Oleoyl-coenzyme A

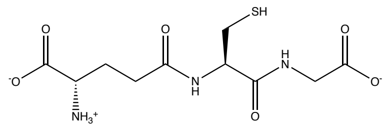

Glutathionate

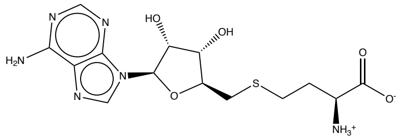

S-adenosyl-L-homocysteine zwitterion

Figure S15: Exemplary molecules in 'common biochemical molecules' database. Molecules shown are commercially available, and thus not incorporated into SCScore based analysis

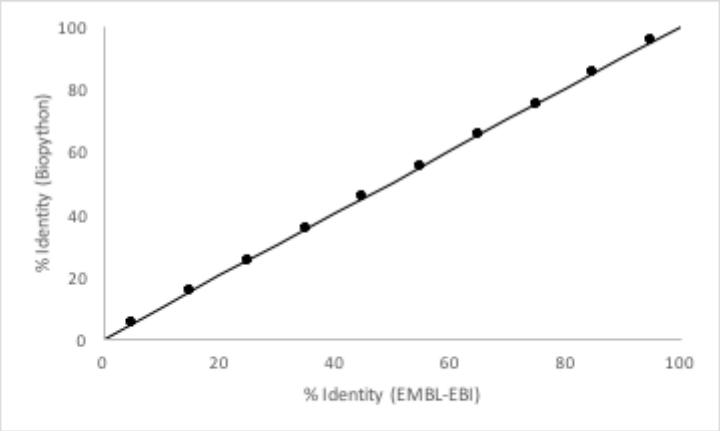

Figure S16: Primary amino acid sequence alignment comparison EMBL-EBI vs. Biopython. Closed circles represent % identity comparisons (range: 5-95%) for 10 pairs of primary amino acid sequences between EMBL-EBI tool and our analogous Biopython implementation. Solid line represents y=x.

Table S3: Uniprot IDs corresponding to amino acid sequences used for EMBL-EBI vs. Biopython comparison and their corresponding % identity score.

UniProt ID 1	UniProt ID 2	% Identity
K4C9E2	P33967	5
E3PRK1	O25536	15
B2HD59	Q9NWW9	25
P26690	P51635	35
E7CQW6	E9L011	45
Q9DAX2	O08564	55
Q60991	O75881	65
Q5KTC7	Q02083	75
Q8HYL8	Q3SZP5	85
B0BND0	Q8BGN3	95

A) Example of positively labeled datapoint (Identity = 58%)

RHEA ID: 44016

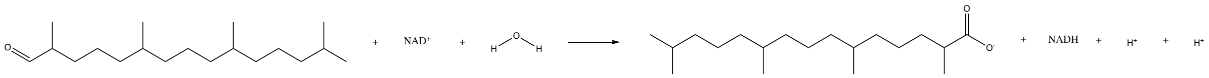

UNIPROT ID: P30839

RHEA ID: 44100

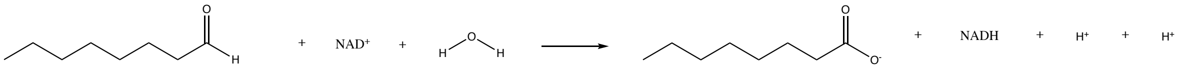

UNIPROT ID: P47739

B) Example of negatively labeled datapoint (Identity = 5.1%)

RHEA ID: 16565

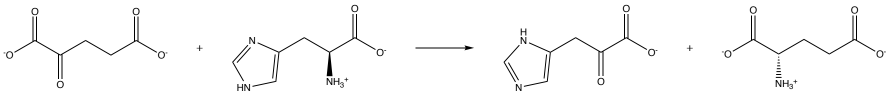

UNIPROT ID: Q8R5Q4

RHEA ID: 44636

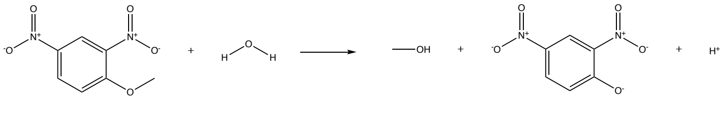

UNIPROT ID: A0A096ZED0

Figure S17: Examples of reaction pairs corresponding to homologous enzymes and negative examples. (A) An example of a pair of homologous enzymes (Identity = 58%). (B) An example of a pair of evolutionarily distant enzymes (Identity = 5.1%). Reaction id and amino acid sequence id are shown as RHEA ID and UNIPROT ID, respectively.

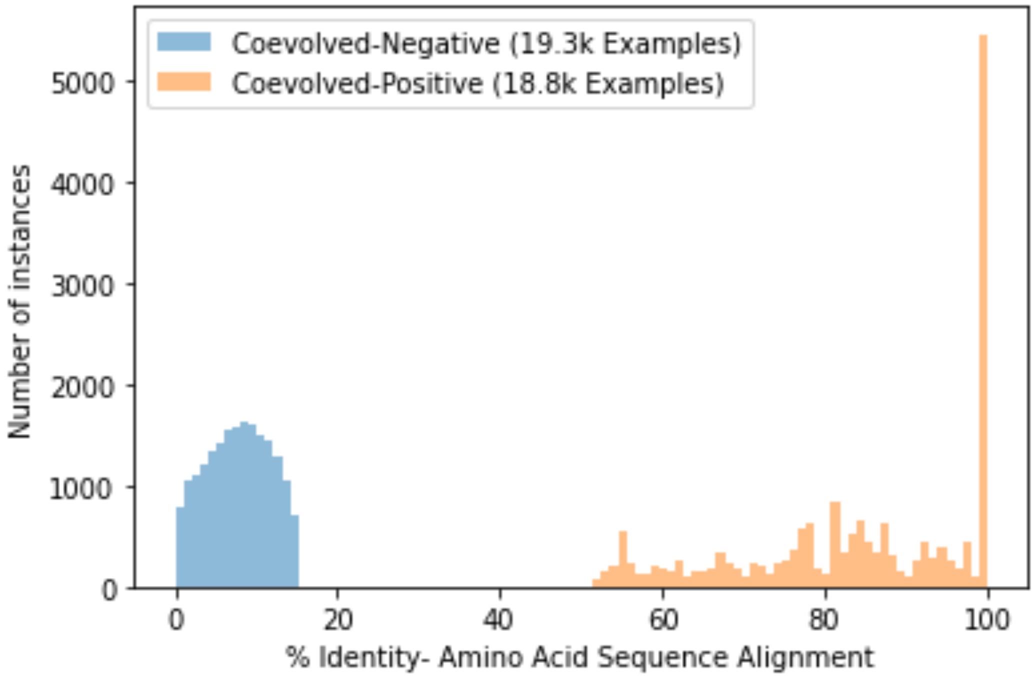

Figure S18: Histogram representing the distribution of % identities of amino acid sequence pairs in the dataset. While ensuring sequence length is longer than 100 amino acids, sequence identities greater than 52% are labeled as positive. On the other hand, sequence identities less than 15% are labeled as negative.

A) RHEA ID: 46141

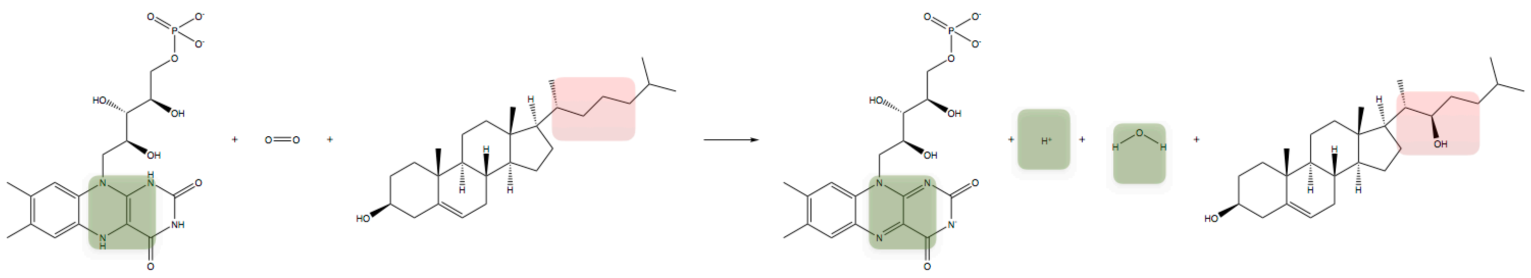

B) RHEA ID: 52289

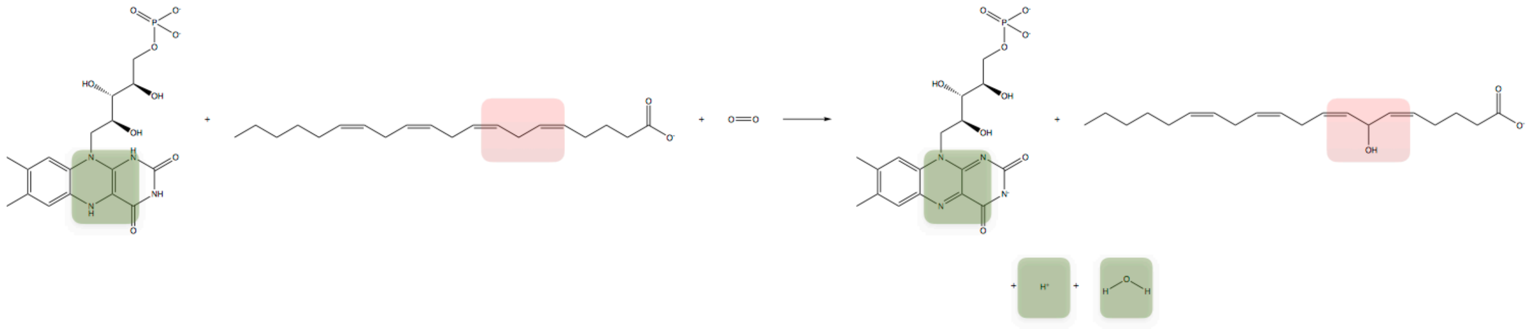

Figure S19: Example (#1) reaction similarity score calculation. The overall reaction similarity, computed using Morgan fingerprints (radius =2, using chirality and features) and the Dice metric, is 0.84. Colors indicate hypothesized atom-level contributions to the overall similarity (green: increases similarity score, red: decreases similarity score, uncolored: has no effect). While there is significant overlap between both transformations, the differences in (a) chirality preferences and (b) the reaction neighborhood surrounding the alcohol (both in red) result in a similarity score less than 1.

A) RHEA ID: 20529

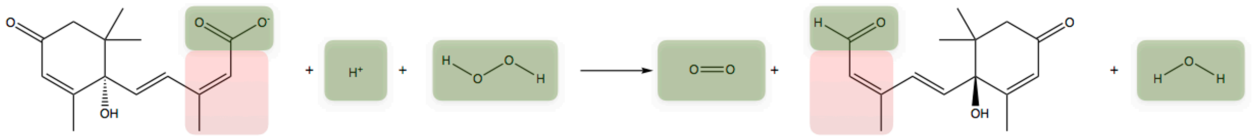

B) RHEA ID: 58992

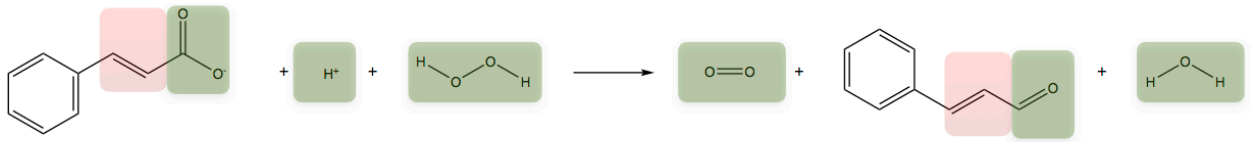

Figure S20: Example (#2) reaction similarity score calculation. The overall reaction similarity, computed using Morgan fingerprints (radius =2, using chirality and features) and the Dice metric, is 0.60. Colors indicate hypothesized atom-level contributions to the overall similarity (green: increases similarity score, red: decreases similarity score, uncolored: has no effect). While both transformations convert a carboxylic acid to an aldehyde, the differences in reaction environment surrounding these functional groups (highlighted in red) result in a similarity score of 0.6.

A) RHEA ID: 23284

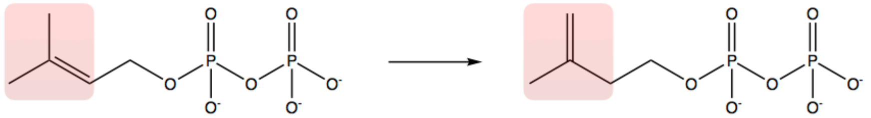

B) RHEA ID: 28518

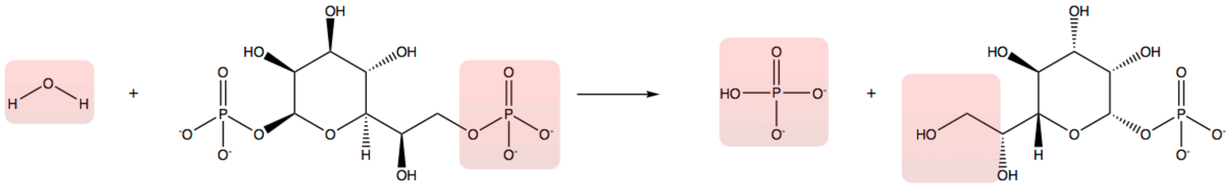

Figure S21: Example (#3) reaction similarity score calculation. The overall reaction similarity, computed using Morgan fingerprints (radius =2, using chirality and features) and the Dice metric, is 0. Colors indicate hypothesized atom-level contributions to the overall similarity (green: increases similarity score, red: decreases similarity score, uncolored: has no effect). The lack of any significant overlap in reaction functional groups reflects a similarity score of 0.

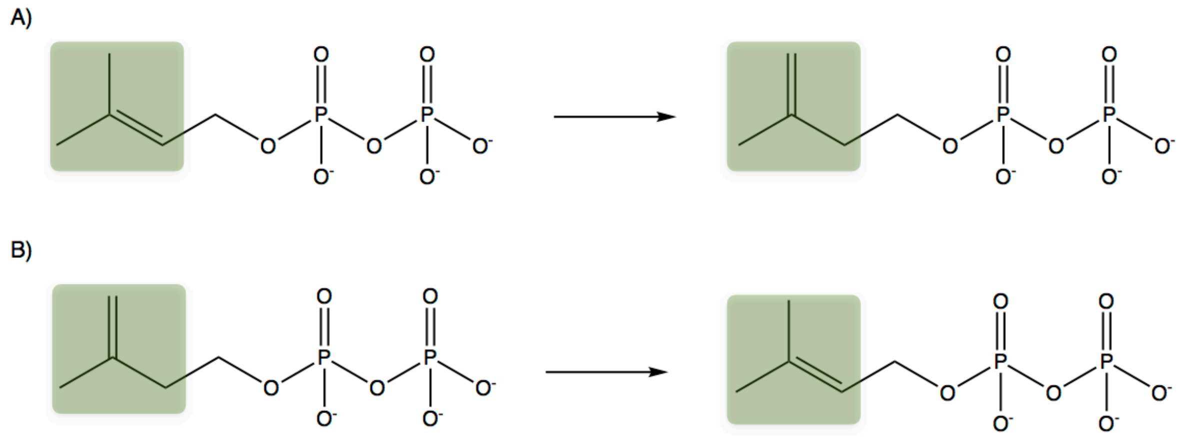

Figure S22: Example (#4) reaction similarity score calculation. The overall reaction similarity, computed using Morgan fingerprints (radius =2, using chirality and features) and the Dice metric, is 1. Colors indicate hypothesized atom-level contributions to the overall similarity (green: increases similarity score, red: decreases similarity score, uncolored: has no effect). Thus, the similarity score is not a function of the direction of the reaction.

Table S4: Comparison between different settings (number of hidden layers, number of nodes per hidden layer) in terms of validation ROC-AUC for evolution scoring.

Number of hidden layers	Number of nodes per hidden layer	Total number of parameters	ROC-AUC (Validation)
3	3	37	0.98
4	4	77	0.98
5	5	141	0.98

Table S5: Parameters required in neural network for evolution scoring.

Layer Name	Input dimension	Output dimension	# Parameters
Hidden Layer 1	2	3	9
Hidden Layer 2	3	3	12
Hidden Layer 3	3	3	12
Output	3	1	4

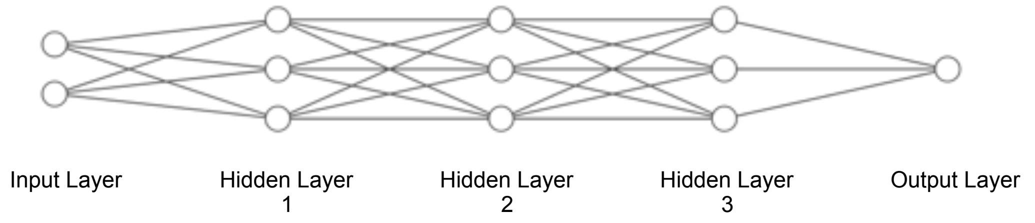

Figure S23: Network architecture corresponding to multilayer perceptron used to determine probability that reaction pairs are similar.

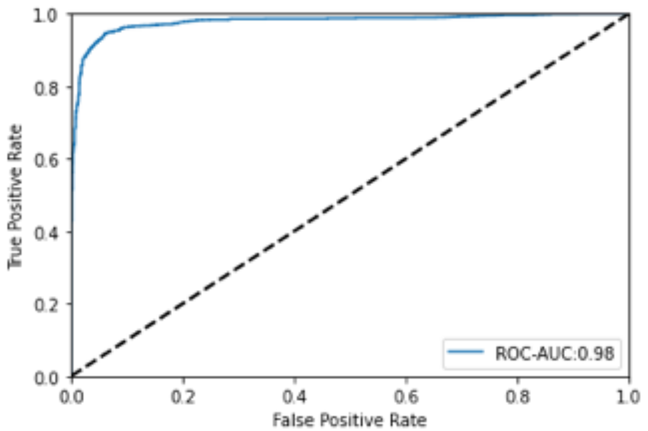

Figure S24: ROC-AUC plot evaluating model performance after training.

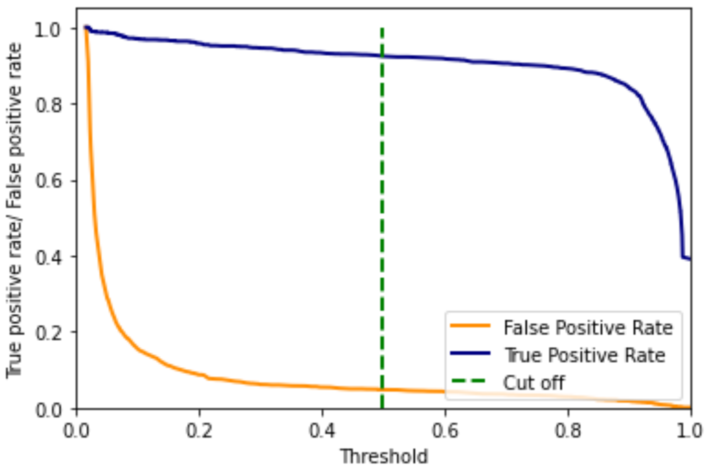

Figure S25: True positive rate and False positive rate as a function of evolution score threshold. A cut off threshold was set at 0.5, and the accuracy at this threshold is 93.9%.

Reaction 1 (RHEA ID: 37999, UniProt ID: Q2QM69) :

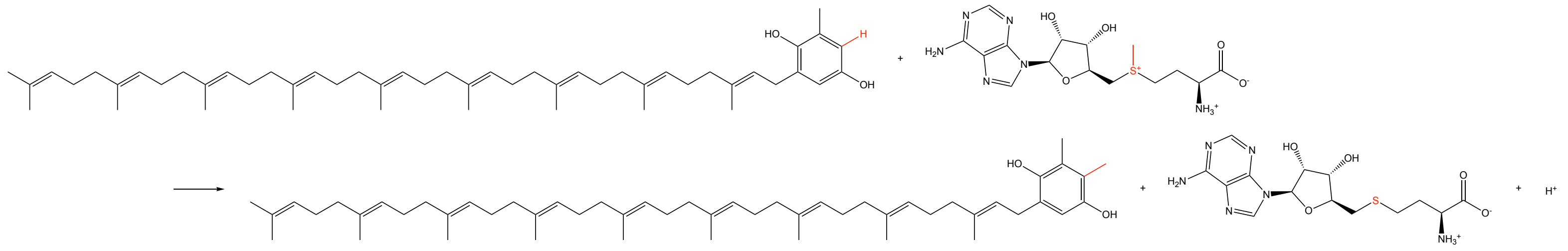

Reaction 2 (RHEA ID: 38007, UniProt ID: P23525):

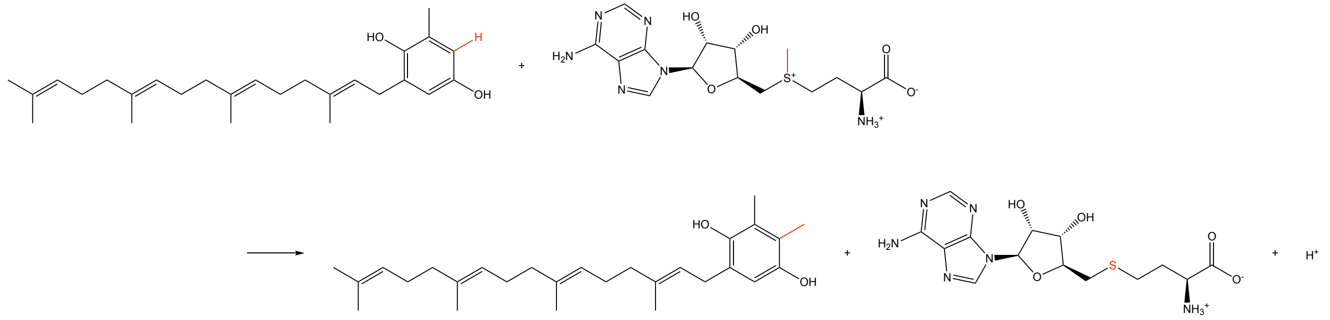

Figure S26: Example reaction pair (#1) selected from the evolution scoring test set. Reactions 1 and 2 transfer a methyl group from S-adenosyl-L-methionine to 2-methyl-6-all-trans-nonaprenylbenzene-1,4-diol and 6-geranylgeranyl-2-methylbenzene-1,4-diol, respectively. The evolution score is 0.98. In our dataset, reactions 1 and 2 are catalyzed by homologous enzymes (66% Identity) with UniProt IDs Q2QM69 and P23525, respectively. We also note that both individual enzymes are known to catalyze both reactions.

Reaction 1 (RHEA ID: 47204, UniProt ID: P56591) :

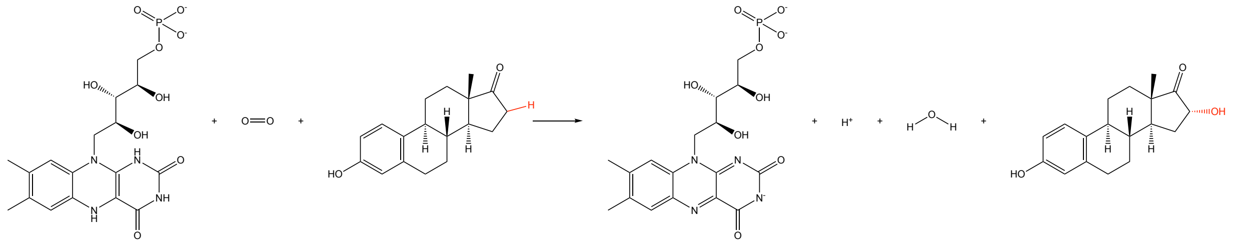

Reaction 2 (RHEA ID: 47313, UniProt ID: Q6GUR1):

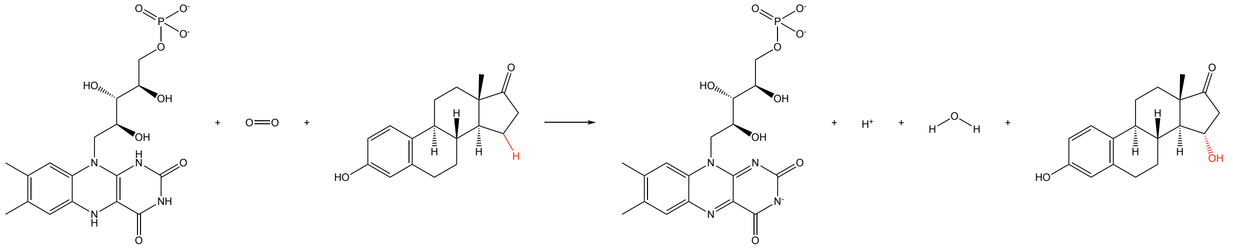

Figure S27: Example reaction pair (#2) selected from the evolution scoring test set. Reactions 1 and 2 describe the hydroxylation of C-H bonds, with differing regiospecificity. The evolution score is 0.99. In our dataset, reactions 1 and 2 are catalyzed by homologous enzymes (80% Identity) with UniProt IDs P56591 and Q6GUR1, respectively. We also note that both individual enzymes are known to catalyze both reactions.

Reaction 1 (RHEA ID: 59364, UniProt ID: Q08DW9) :

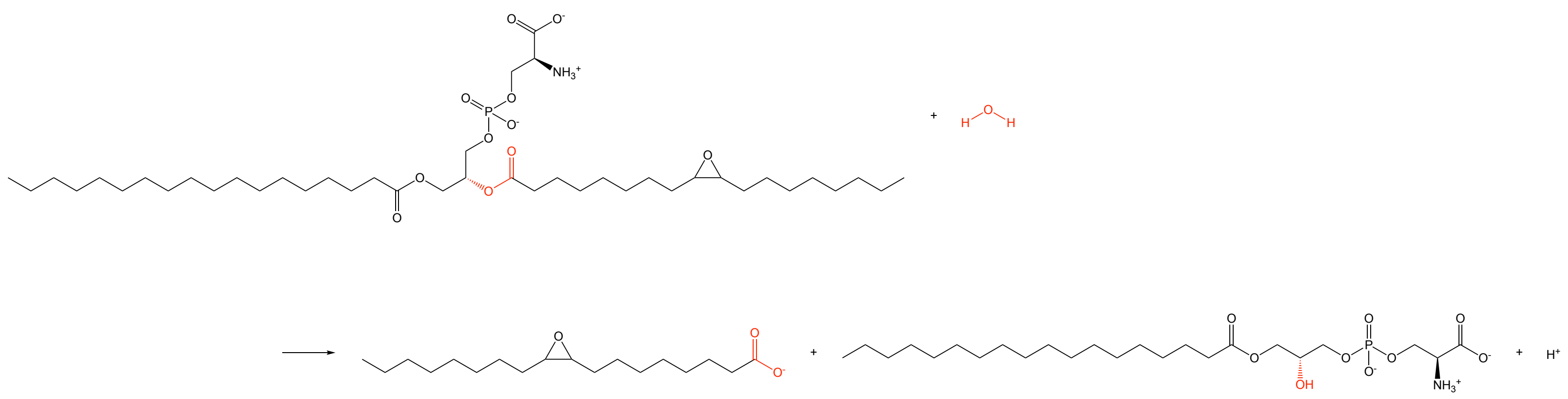

Reaction 2 (RHEA ID: 59372, UniProt ID:Q6AYT7):

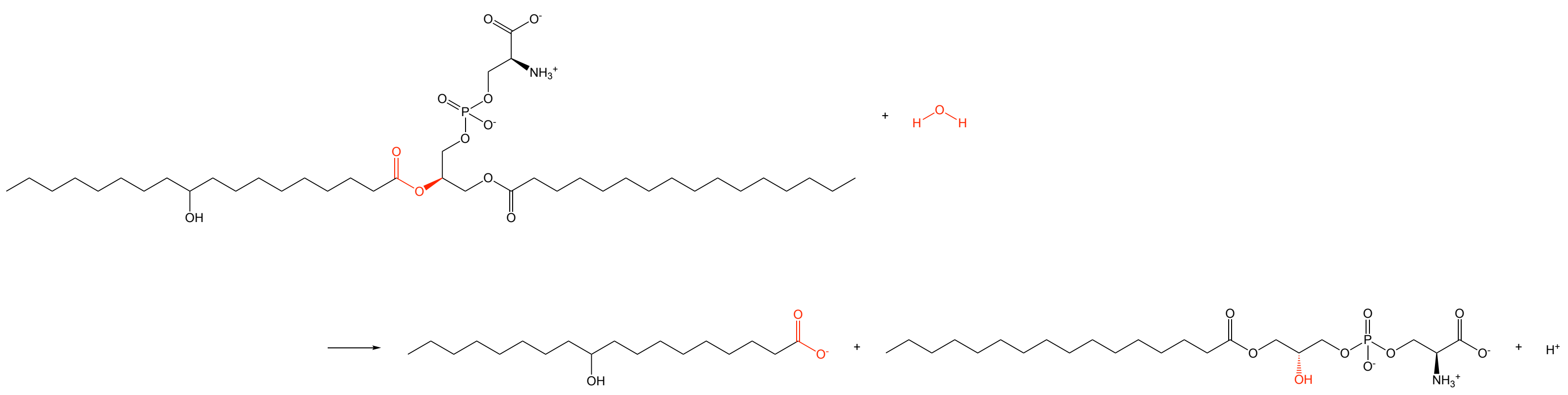

Figure S28: Example reaction pair (#3) selected from the evolution scoring test set. Reactions 1 and 2 describe the hydrolysis of an ester bond, with different substrate specificities. The evolution score is 0.99. In our dataset, reactions 1 and 2 are catalyzed by homologous enzymes (92% Identity) with UniProt IDs Q08DW9 andQ6AYT7, respectively. We also note that both individual enzymes are known to catalyze both reactions.

Reaction 1 (RHEA ID: 20665, UniProt ID: P82125) :

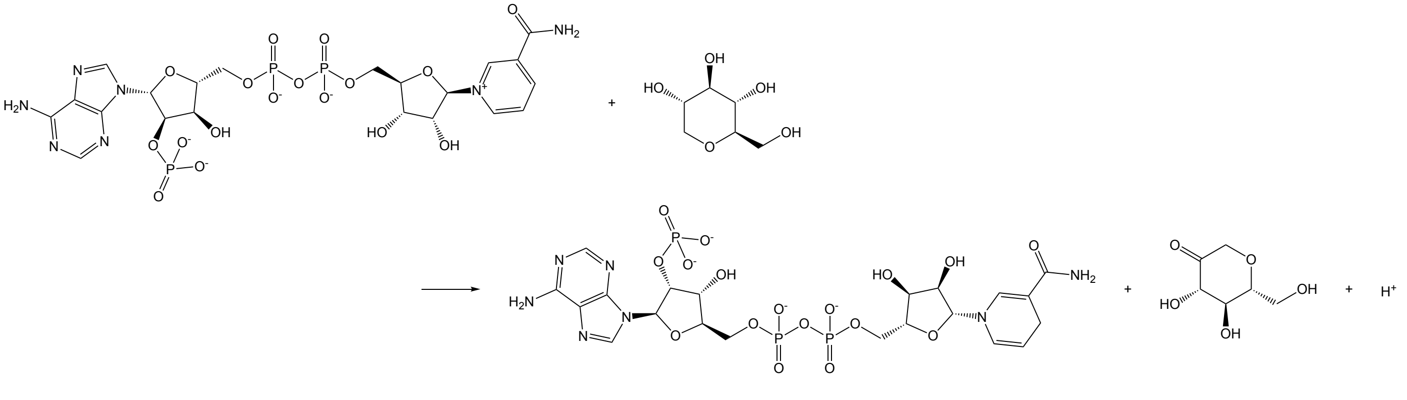

Reaction 2 (RHEA ID: 45312, UniProt ID: P45376):

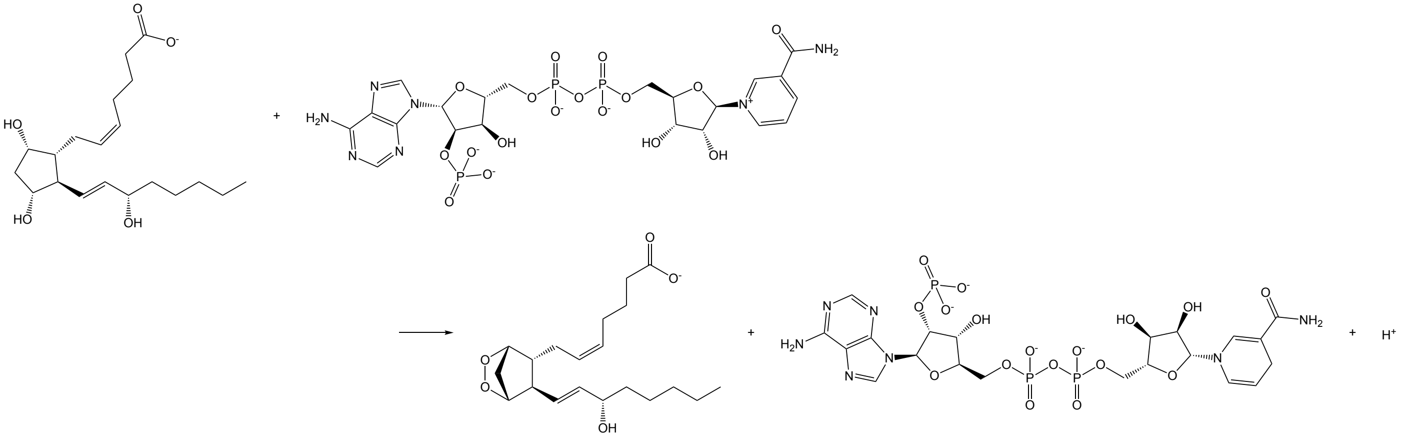

Figure S29: Example reaction pair (#4) selected from the evolution scoring test set. Reactions 1 and 2 describe oxidation reactions, with different substrate specificities. The evolution score is 0.95. In our dataset, reactions 1 and 2 are catalyzed by homologous enzymes (60% Identity) with UniProt IDs P82125 and P45376, respectively.

Reaction 1 (RHEA ID: 22868, UniProt ID: P0ABK5) :

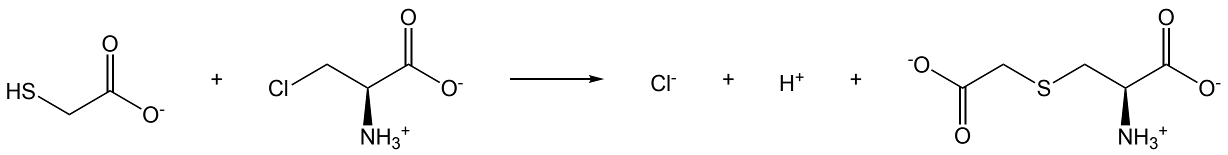

Reaction 2 (RHEA ID: 47333, UniProt ID: P10632):

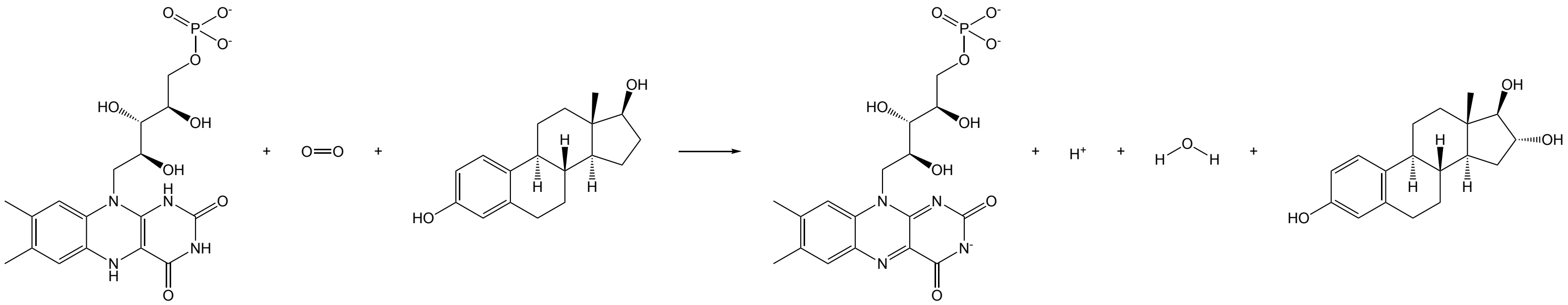

Figure S30: Example reaction pair (#5) selected from the evolution scoring test set. Reaction 1 describes a carbon-halide lyase, while reaction 2 describes an oxidation reaction catalyzed by P450 enzyme. The evolution score is 0.02. In our dataset, reactions 1 and 2 are catalyzed by enzymes with UniProt IDs P0ABK5 and P10632, respectively (14.7% identity).

Reaction 1 (RHEA ID: 47312, UniProt ID: P05176) :

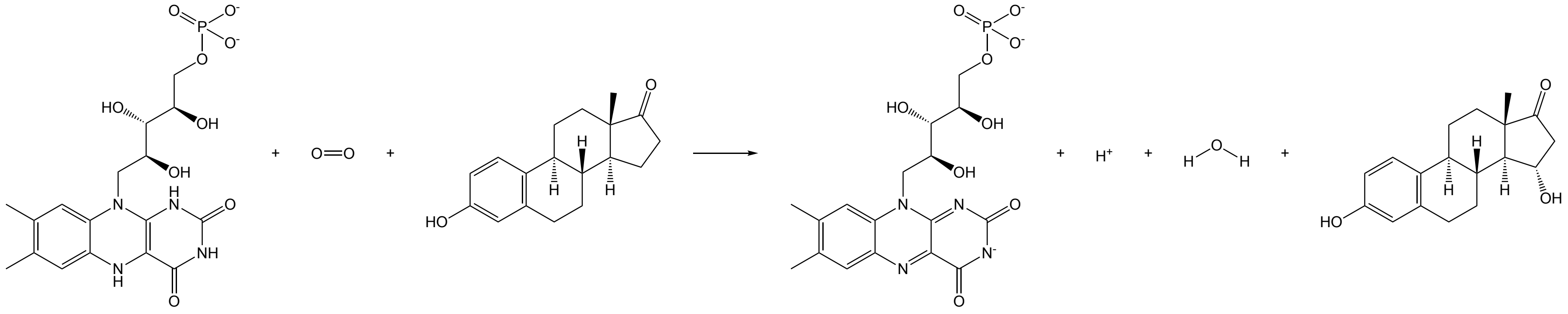

Reaction 2 (RHEA ID: 48636, UniProt ID: P05176):

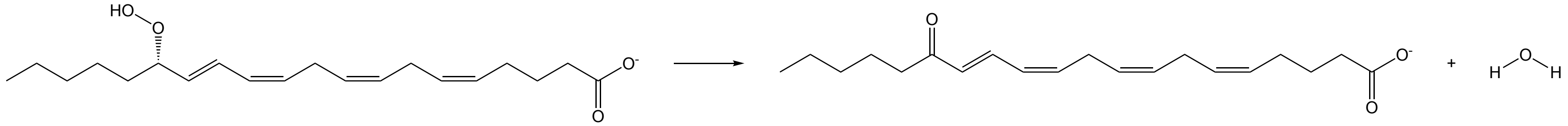

Figure S31: Example reaction pair (#6) selected from the evolution scoring test set. Reaction 1 describes a monooxygenase; it facilitates the incorporation of molecular oxygen into the substrate. On the other hand, reaction 2 describes the conversion of a hydroperoxide species into an oxo metabolite. The evolution score is 0.02. In our dataset, reactions 1 and 2 are both catalyzed by the same enzyme with UniProt ID P05176. The broad substrate and reactive scope of P450 enzymes is sometimes not captured by the model.

Reaction 1 (RHEA ID: 18841, UniProt ID: P77161) :

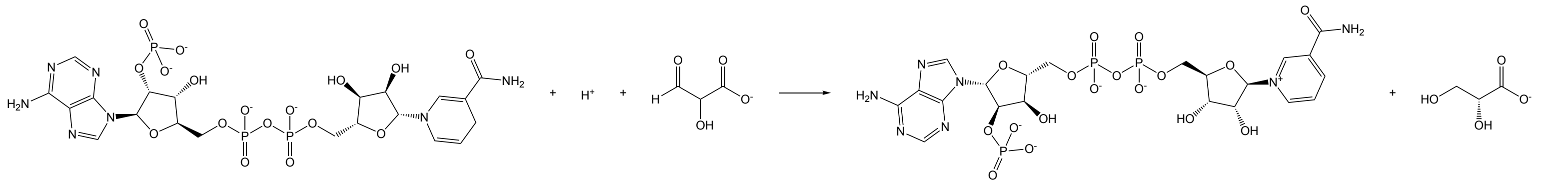

Reaction 2 (RHEA ID: 21176, UniProt ID: P39346):

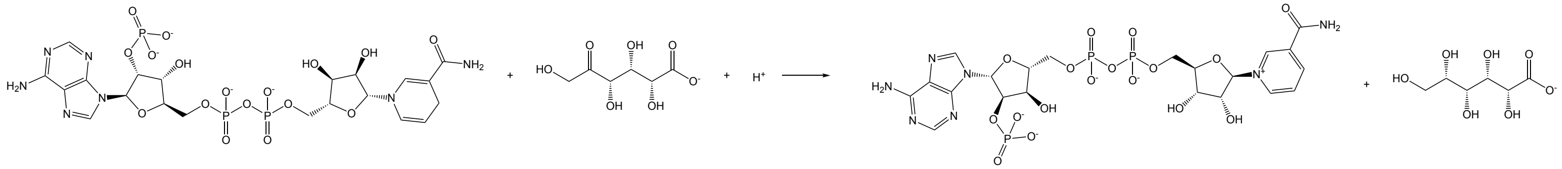

Figure S32: Example reaction pair (#7) selected from the evolution scoring test set. Reaction 1 describes the reduction of an aldehyde to an alcohol, while reaction 2 describes the reduction of a ketone to an alcohol. Both enzymes are NADP+/NADPH dependent. The evolution score is 0.98. In our dataset, reactions 1 and 2 are catalyzed by enzymes with UniProt IDs P77161 and P39346, respectively (10% identity). This pair is likely a false positive because (1) both describe reduction reactions resulting in a high reaction fingerprint similarity score (2) NADP+/NADPH, in lieu of the substrates, contributes significantly to the high overall molecular similarity score.

Table S6: Reaction- and overall molecular- similarity feature values for the negative examples presented in Figure 6 (5). The predicted evolution score and % identity sequence alignment value are also shown.

	Reaction Similarity	Overall molecular similarity	Evolution Score	Sequence Alignment (% Identity)
**Figure 6, 5A**	0.56	0.07	0.14	8.1
**Figure 6, 5B**	0.43	0.18	0.16	6.6
**Figure 6, 5C**	0	0.54	0.23	3.1
**Figure 6, 5D**	0	0.02	0.02	7.7

Reaction 1 (RHEA ID: 23416, Uniprot ID: Q67NP7):

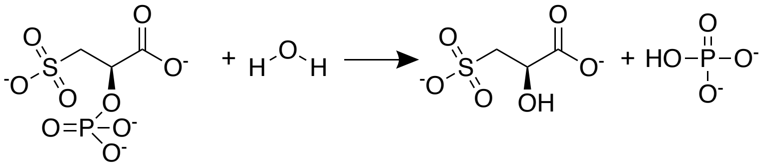

Reaction 2 (RHEA ID: 27514, Uniprot ID: Q9BX95):

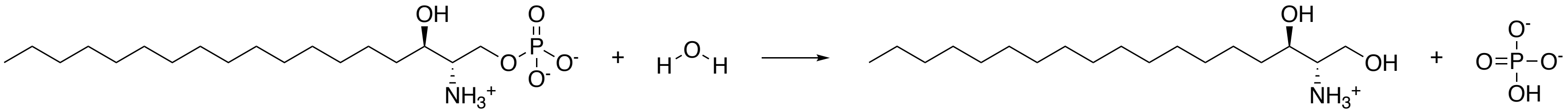

Figure S33: Reaction details for Figure 6 (5A), a generated negative pair. Reactions 1 and 2 describe phosphatase transformations, with drastically different substrates. The evolution score is 0.14. In our dataset, reactions 1 and 2 are catalyzed by enzymes with UniProt IDs Q67NP7 and Q9BX95, respectively (8.1% identity).

Reaction 1 (RHEA ID: 10316, Uniprot ID: P86937)

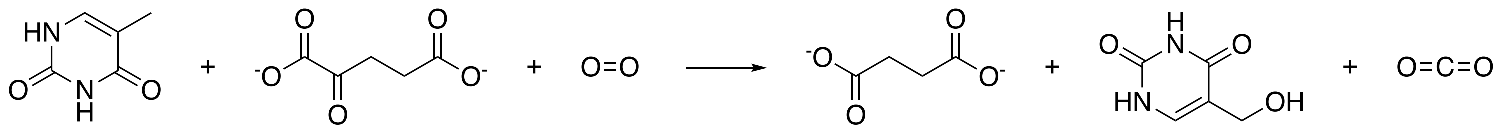

Reaction 2 (RHEA ID: 52908, Uniprot ID: Q8GHB1)

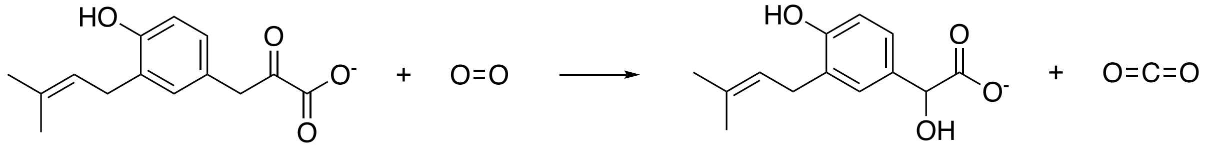

Figure S34: Reaction details for Figure 6 (5B), a generated negative pair. Reactions 1 and 2 describe dioxygenase transformations, with drastically different substrates. The evolution score is 0.16. In our dataset, reactions 1 and 2 are catalyzed by enzymes with UniProt IDs P86937 and Q8GHB1, respectively (6.6% identity).

Reaction 1 (RHEA ID- 35883, Uniprot ID- Q6NYV8):

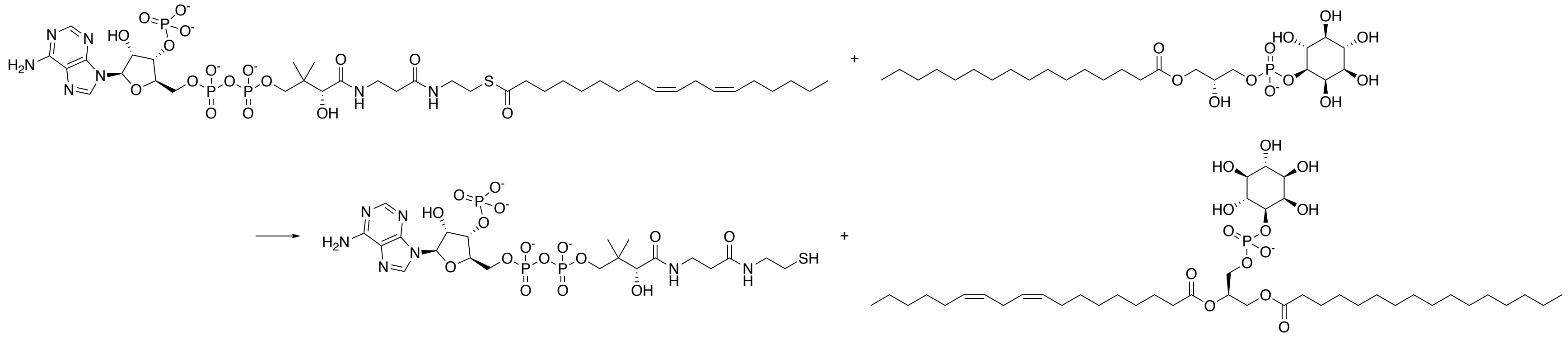

Reaction 2 (RHEA ID- 40183, Uniprot ID- P07872):

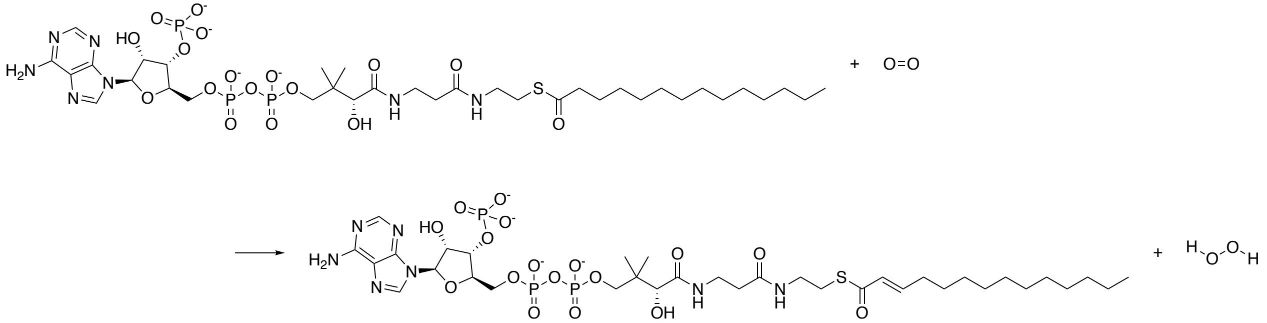

Figure S35: Reaction details for Figure 6 (5C), a generated negative pair. Reactions 1 and 2 both accept acyl-CoA as substrates, but catalyze different reactions. Reaction 1 is associated with an acyl transferase, while reaction 2 belongs to an oxidase. The evolution score is 0.23. In our dataset, reactions 1 and 2 are catalyzed by enzymes with UniProt IDs Q6NYV8 and P07872, respectively (3.1% identity).

Reaction 1 (RHEA ID: 11584, Uniprot ID: P32960):

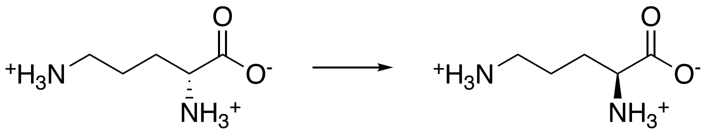

Reaction 2 (RHEA ID: 21664, Uniprot ID: P19881):

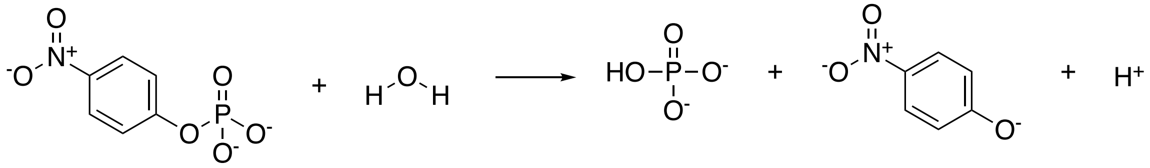

Figure S36: Reaction details for Figure 6 (5D), a generated negative pair. Reaction 1 is associated with a racemase, while reaction 2 belongs to a phosphatase. The substrate of reaction 1 is not chemically similar to that of reaction 2. The evolution score is 0.02. In our dataset, reactions 1 and 2 are catalyzed by enzymes with UniProt IDs P32960 and P19881, respectively (7.7% identity).

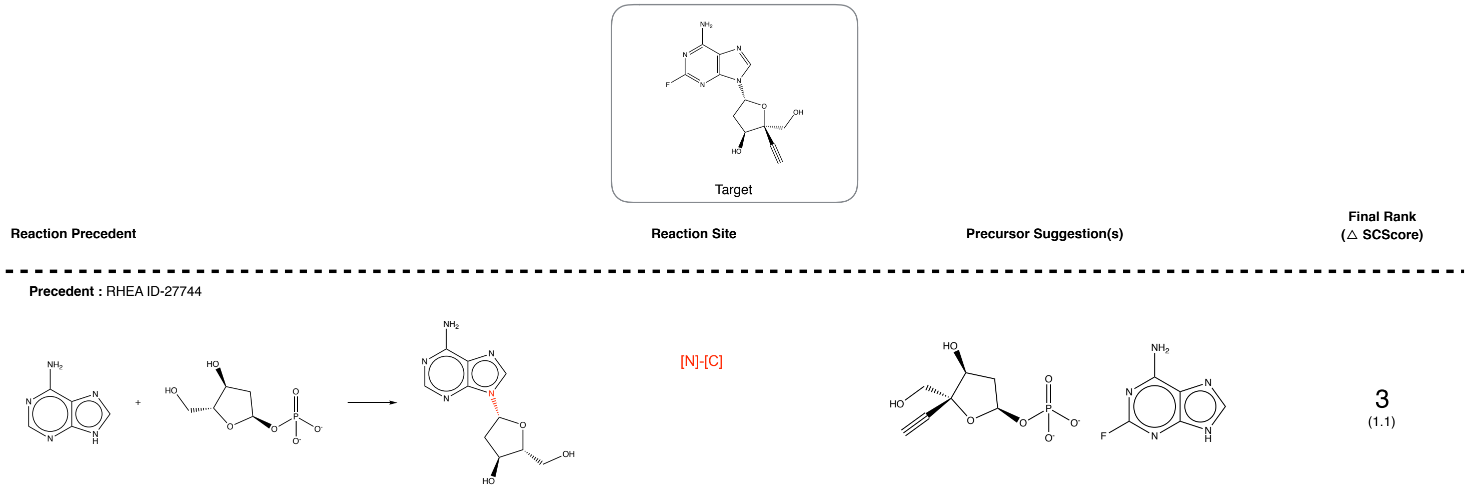

Figure S37: Single step retrosynthetic analysis for Islatravir (Compound 1). A purine nucleoside phosphorylase displaces the phosphate with a nucleobase to yield the target. The evolution score for the proposed transformation is (0.98/1).

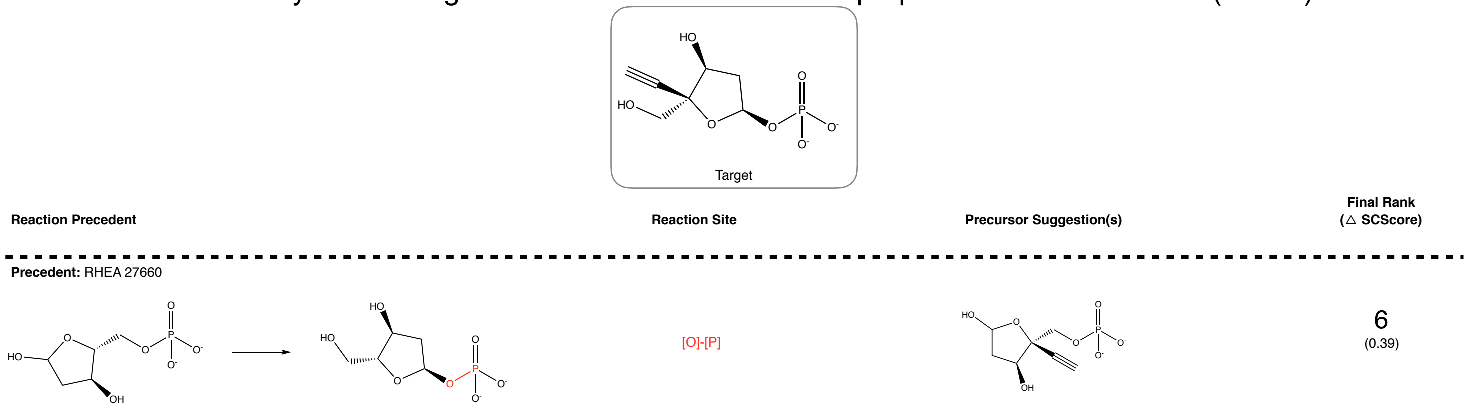

Figure S38: Single step retrosynthetic analysis for intermediate compound 2 in Islatravir enzymatic synthesis. A phosphopentomutase transfers the phosphate group from 5- to 1-position. The evolution score for the proposed transformation is (0.92/1).

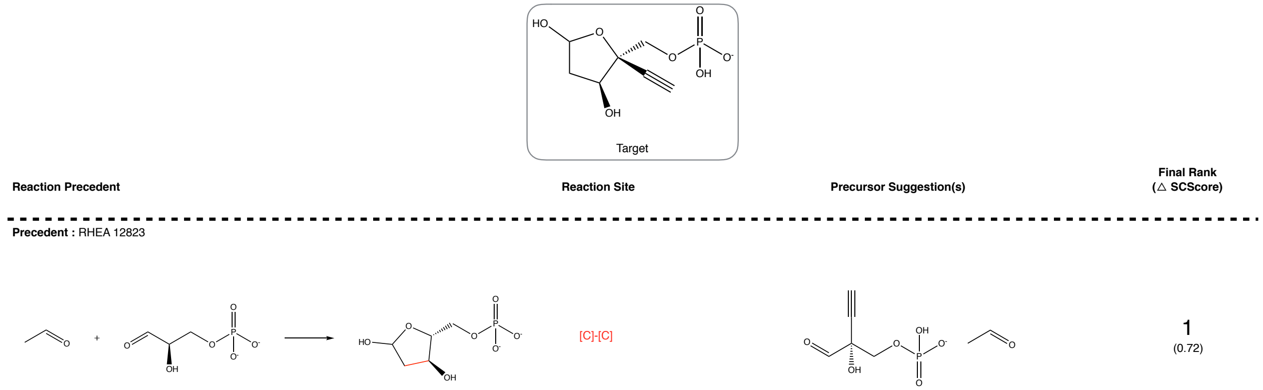

Figure S39: Single step retrosynthetic analysis for intermediate compound 3 in Islatravir enzymatic synthesis. A deoxyribose 5-phosphate aldolase (DERA) catalyzes the forward aldol reaction converting a glyceraldehyde 3-phosphate analogue and acetaldehyde to the sugar 5-phosphate. The evolution score for the proposed transformation is (0.91/1).

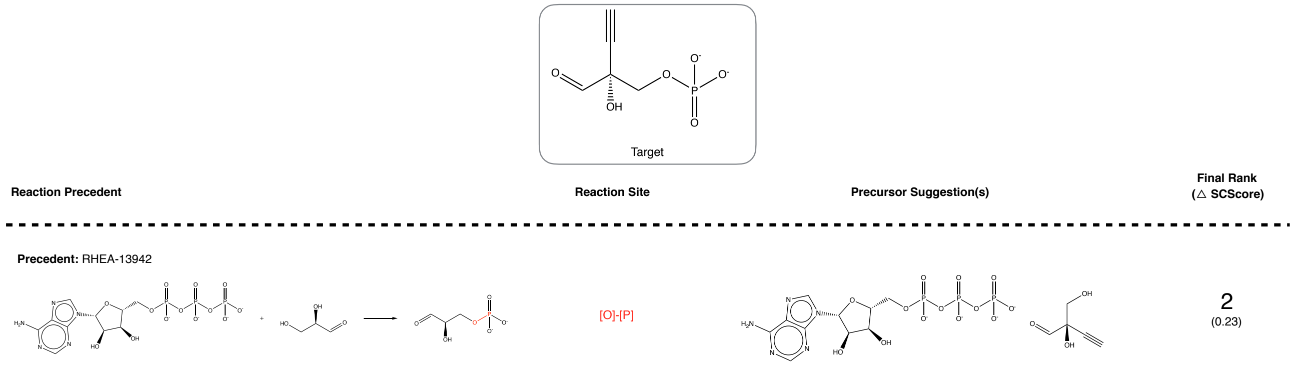

Figure S40: Single step retrosynthetic analysis for intermediate compound 4 in Islatravir enzymatic synthesis. A kinase is used to transfer a phosphate group from ATP (donor) to 2-ethynylglyceraldehyde to yield the target. The evolution score for the proposed transformation is (0.99/1).

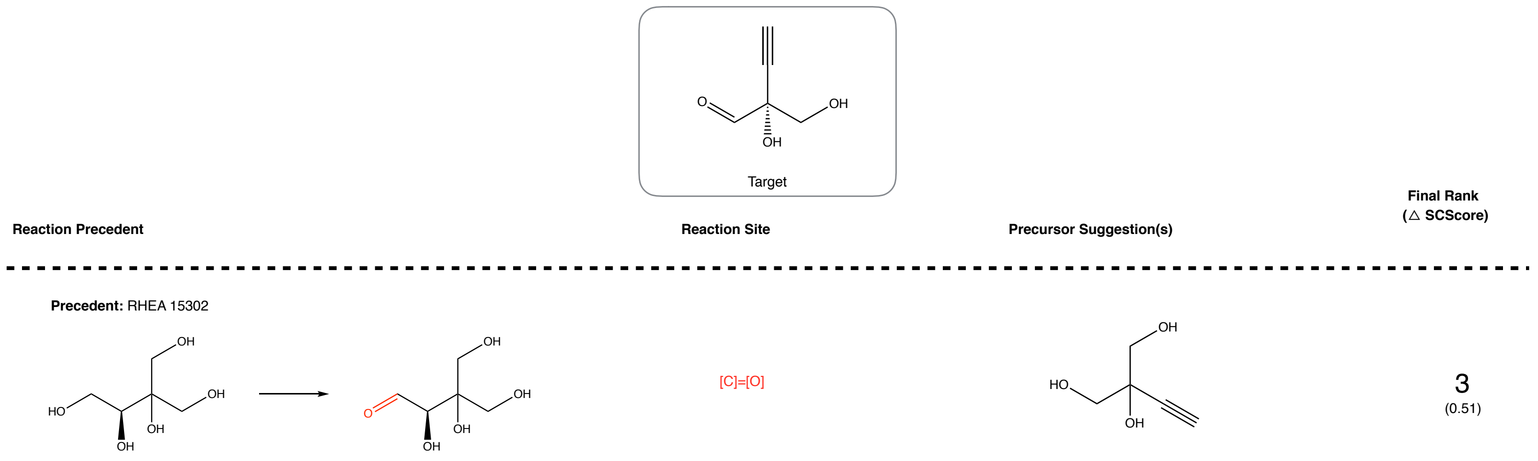

Figure S41: Single step retrosynthetic analysis for intermediate compound 5 in Islatravir enzymatic synthesis. An oxidoreductase is used to convert 2-ethynylglycerol to 2-ethynylglyceraldehyde. The evolution score for the proposed transformation is (0.996/1).

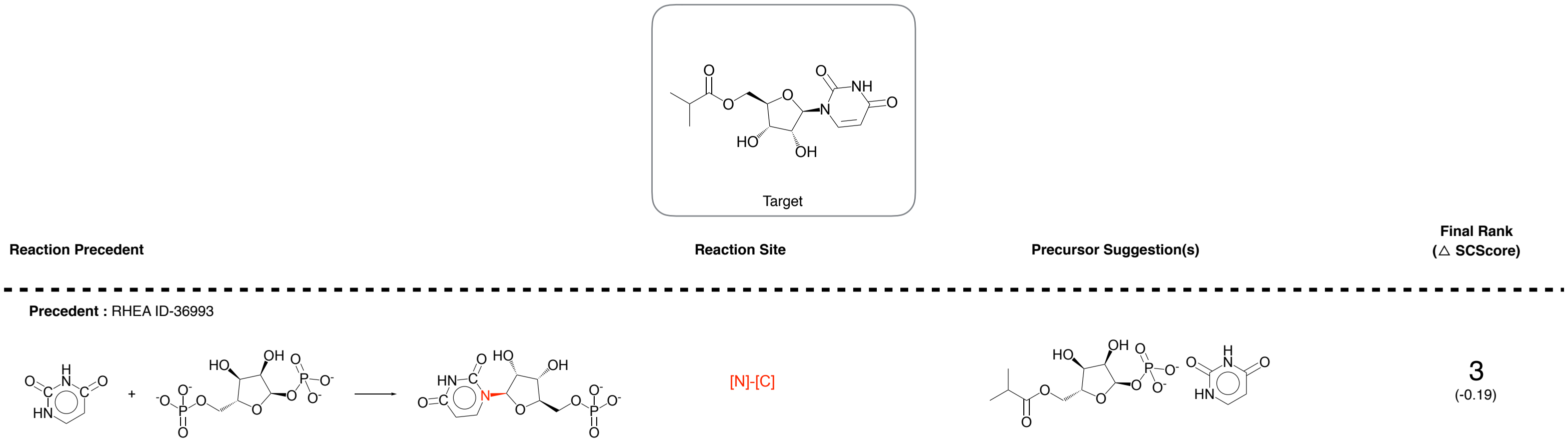

Figure S42: Single step retrosynthetic analysis for intermediate compound 7 in Molnupiravir enzymatic synthesis. A nucleoside phosphorylase displaces the phosphate with a nucleobase to yield the target. The evolution score for the proposed transformation is (0.98/1).

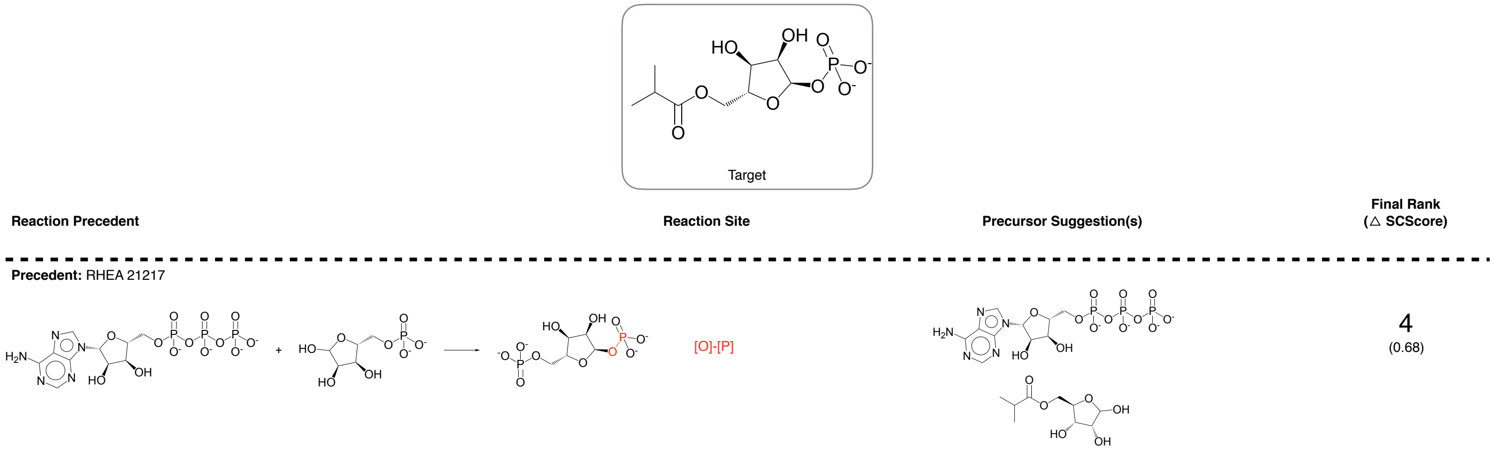

Figure S43: Single step retrosynthetic analysis for intermediate compound 8 in Molnupiravir enzymatic synthesis. A phosphate transferase/ kinase transfers the phosphate group from a donor (e.g. ATP) to yield the target. The evolution score for the proposed transformation is (0.98/1).

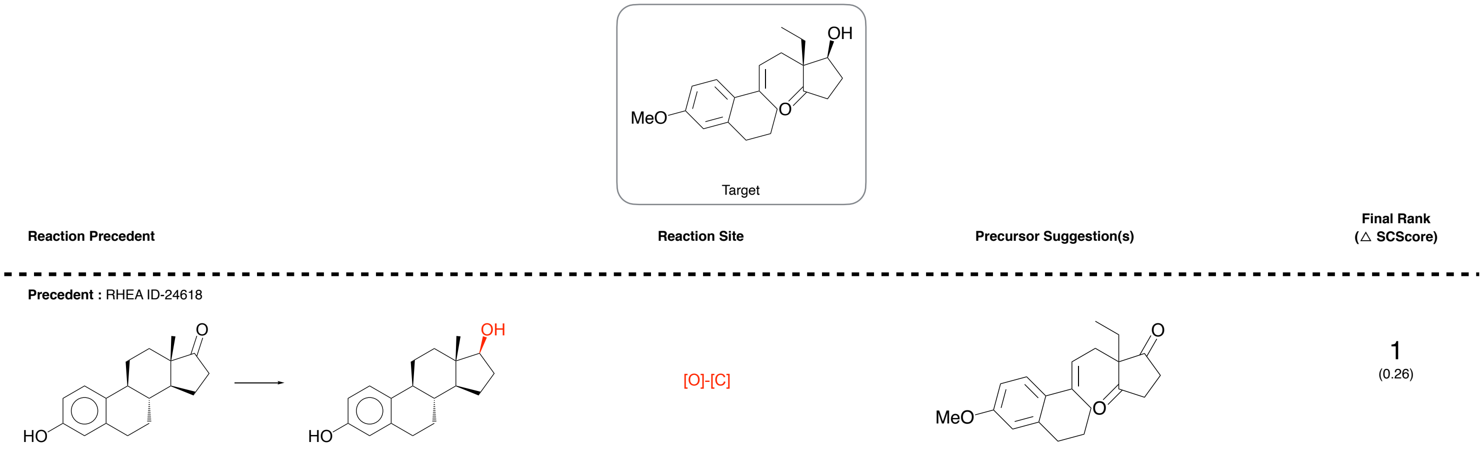

Figure S44: Single step retrosynthetic analysis for (13R,17S) -ethyl secol (Compound 10). An alcohol dehydrogenase catalyzes the regio- and stereo-selective reductive desymmetrization of ethyl secodione to yield the target. The evolution score for the proposed transformation is (0.99/1).

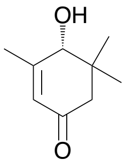

Target

Reaction Precedent **Reaction Site** **Precursor Suggestion(s)** **Final Rank (△ SCScore)**

Precedent : RHEA ID-13526

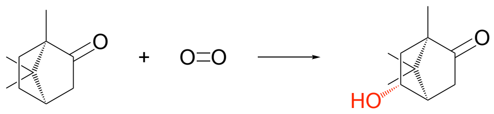

[O]-[C]

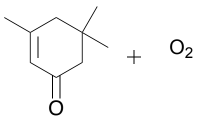

2
(1.33)

Figure S45: Single step retrosynthetic analysis for (R)-4-hydroxy isophorone (Compound 11). A P450 monooxygenase catalyzes the regio- and stereo- selective oxidation of α-isophorone to yield the target. The evolution score for the proposed transformation is (0.91/1).

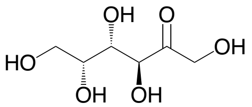

Target

Reaction Precedent **Reaction Site** **Precursor Suggestion(s)** **Final Rank (△ SCScore)**

Precedent : RHEA ID-20434

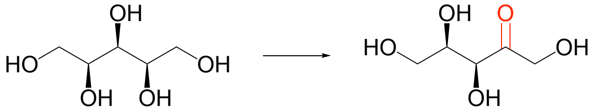

[O]=[C]

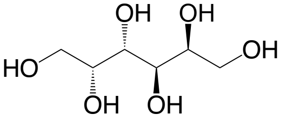

1
(0.59)

Figure S46: Single step retrosynthetic analysis for D-tagatose (Compound 12). A polyol dehydrogenase catalyzes the oxidation of the polyol galactitol to the desired ketose. The evolution score for the proposed transformation is (0.996/1).

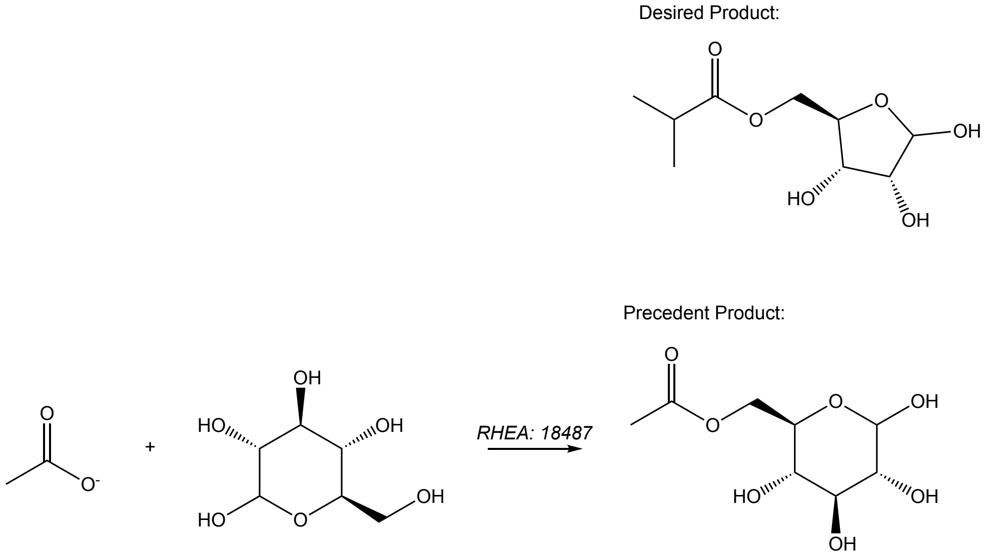

Figure S47: Alternative suggestion to catalyze the selective esterification of the ribose sugar using an isobutyryl donor. We hypothesize that 6-acetylglucose-deacetylase (RHEA:18487) is a potential candidate to facilitate the esterification reaction because it yields a chemically similar product (product similarity score = 0.85)

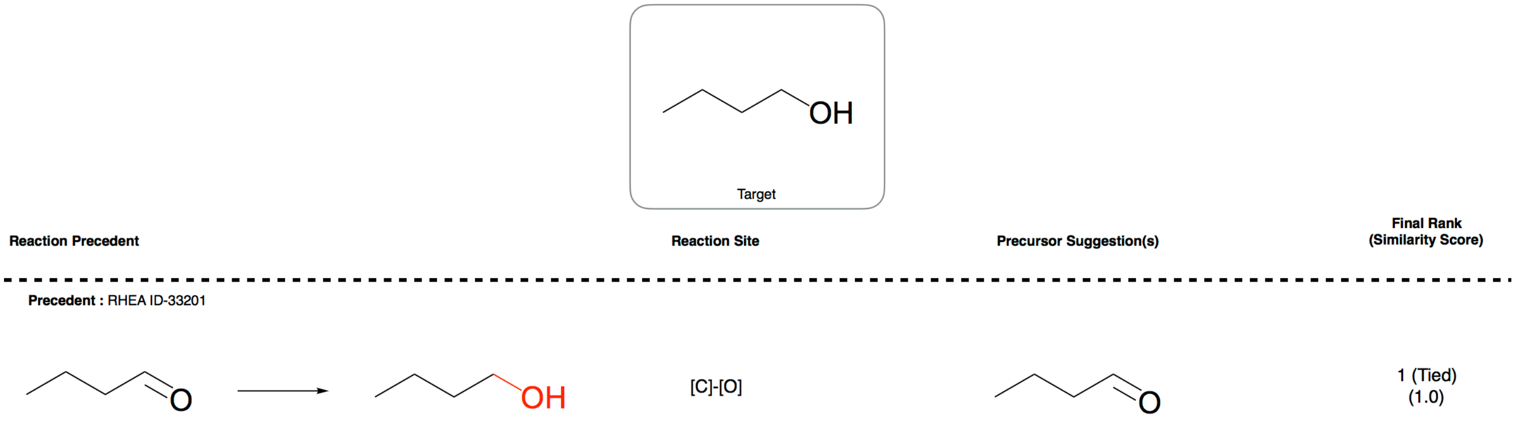

Figure S48: Single step retrosynthetic analysis for 1-butanol synthesis (Compound 13). An alcohol dehydrogenase converts the aldehyde to alcohol. An exact literature precedent is available to support this recommendation.

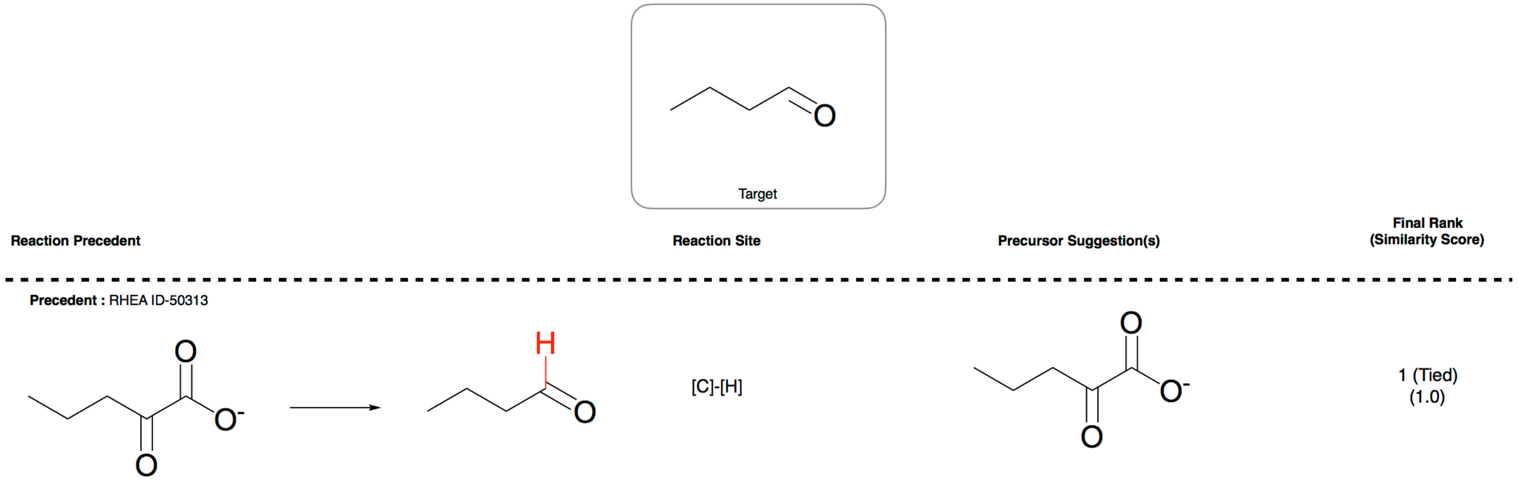

Figure S49: Single step retrosynthetic analysis for intermediate compound 14 in 1-butanol synthesis. A 2-keto-acid decarboxylase converts the 2-keto acid to an aldehyde. An exact literature precedent is available to support this recommendation.

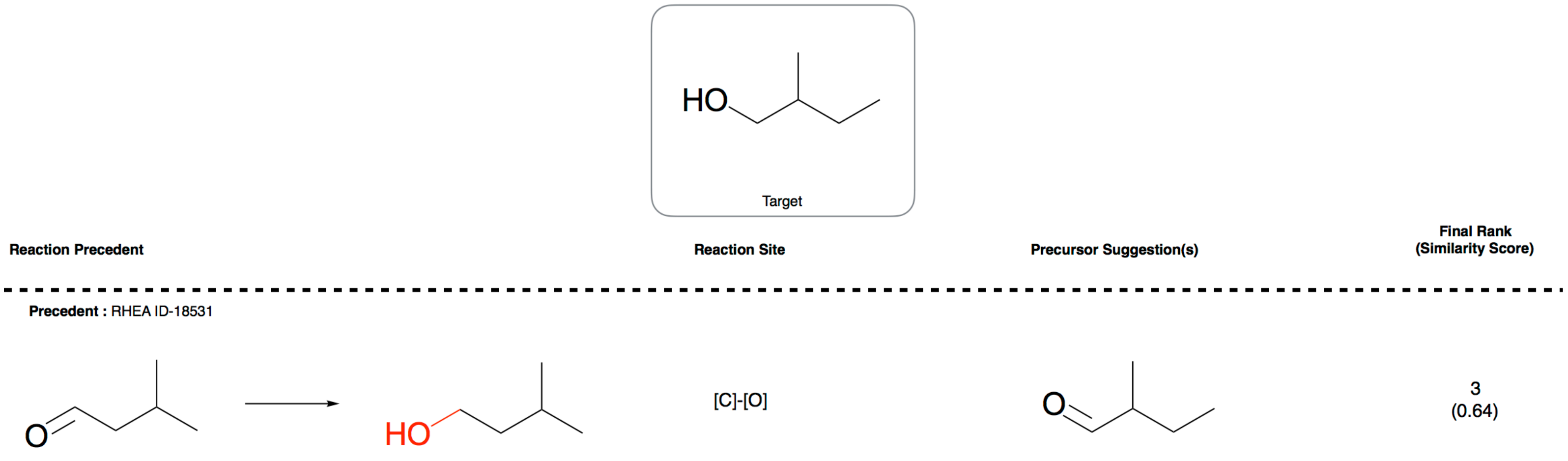

Figure S50: Single step retrosynthetic analysis for 2-methyl-1-butanol (Compound 15) synthesis. An alcohol dehydrogenase converts the aldehyde to alcohol. The evolution score for the proposed transformation is (0.996/1).

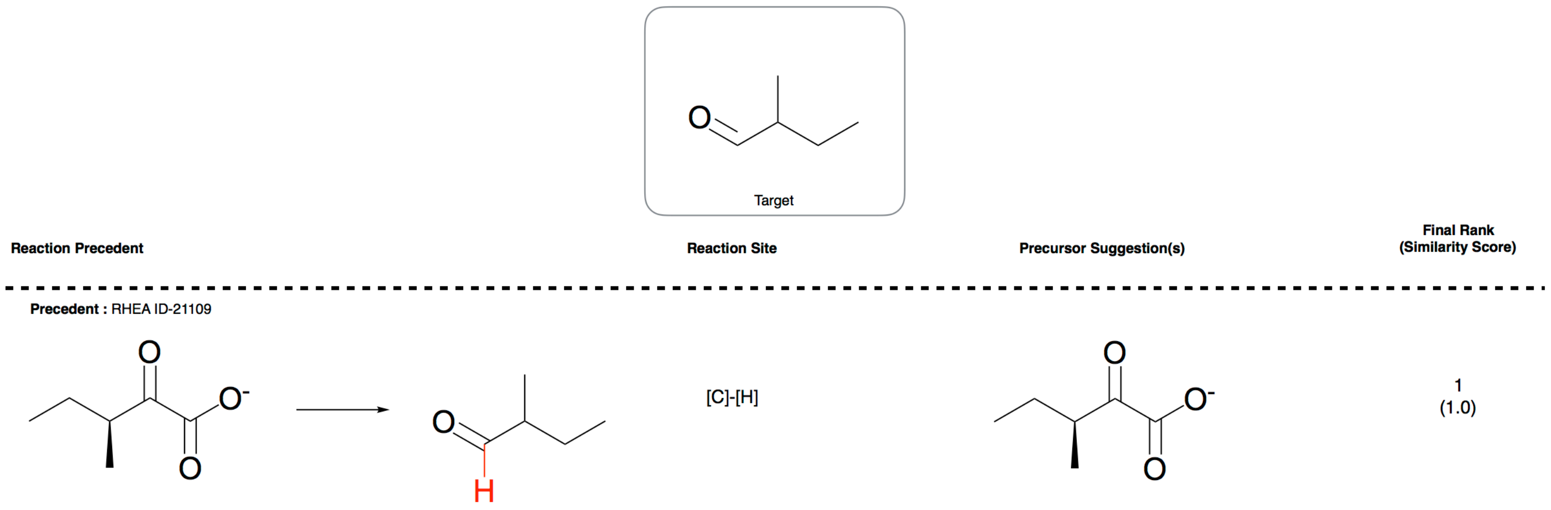

Figure S51: Single step retrosynthetic analysis for the intermediate compound 16 in 2-methyl-1-butanol synthesis. A 2-keto-acid decarboxylase converts the 2-keto acid to an aldehyde. An exact literature precedent is available to support this recommendation.

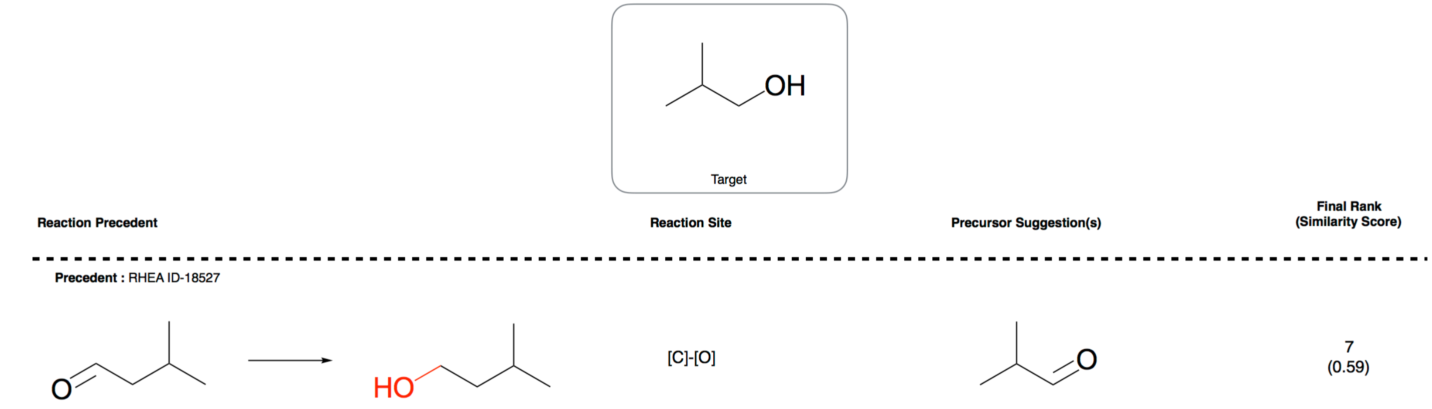

Figure S52: Single step retrosynthetic analysis for isobutanol synthesis (Compound 17). An alcohol dehydrogenase converts the aldehyde to alcohol. The evolution score for the proposed transformation is (0.996/1).

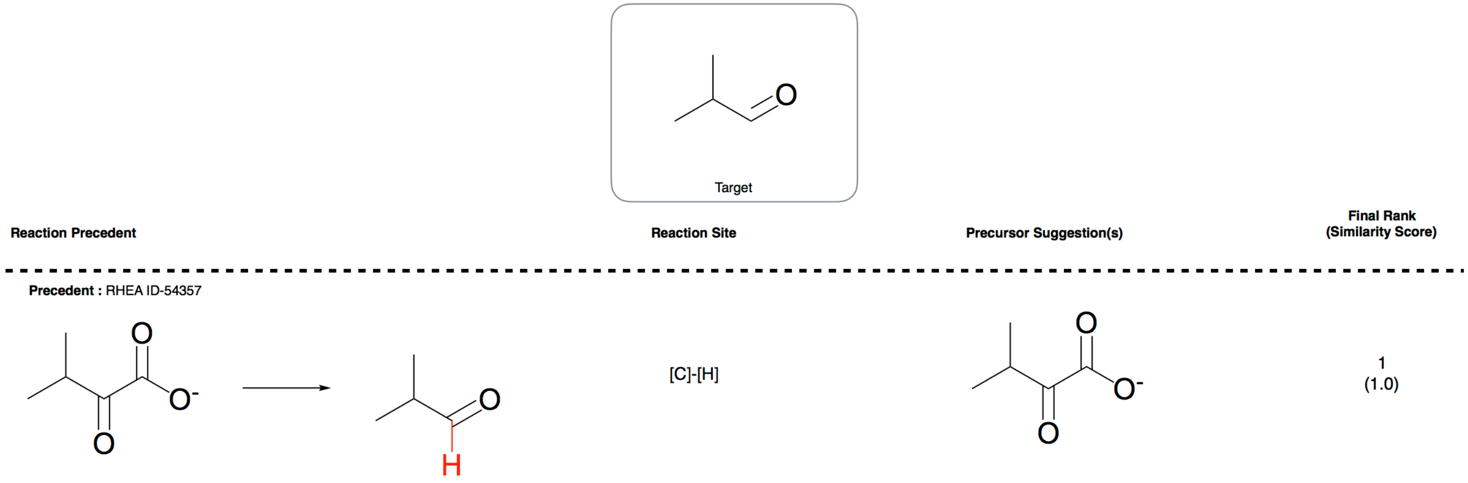

Figure S53: Single step retrosynthetic analysis for the intermediate compound 18 in isobutanol synthesis. A 2-keto-acid decarboxylase converts the 2-keto acid to an aldehyde. An exact literature precedent is available to support this recommendation.

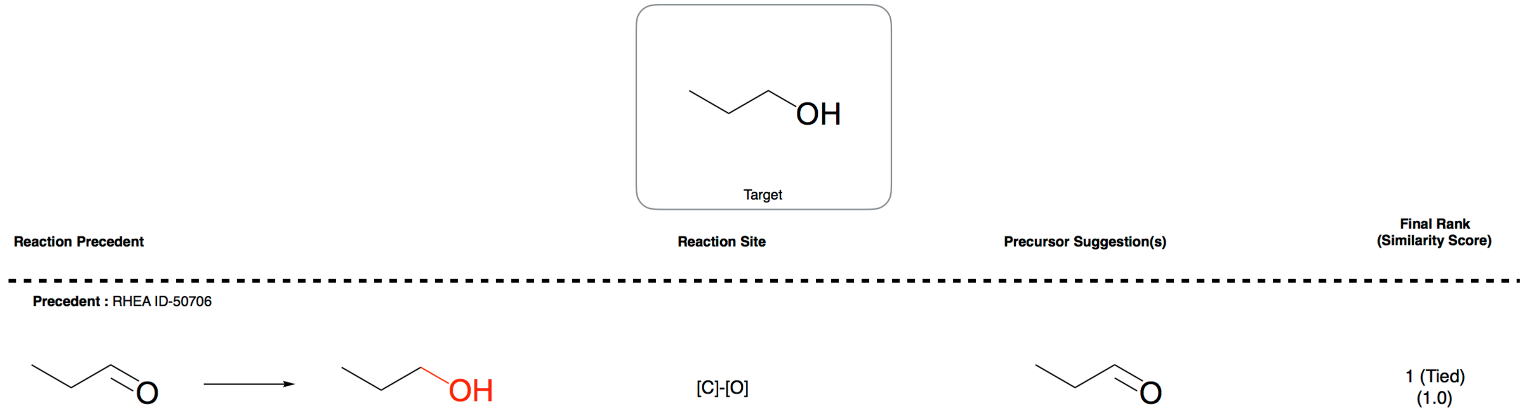

Figure S54: Single step retrosynthetic analysis for 1-propanol synthesis (Compound 19). An alcohol dehydrogenase converts the aldehyde to alcohol. An exact literature precedent is available to support this recommendation.

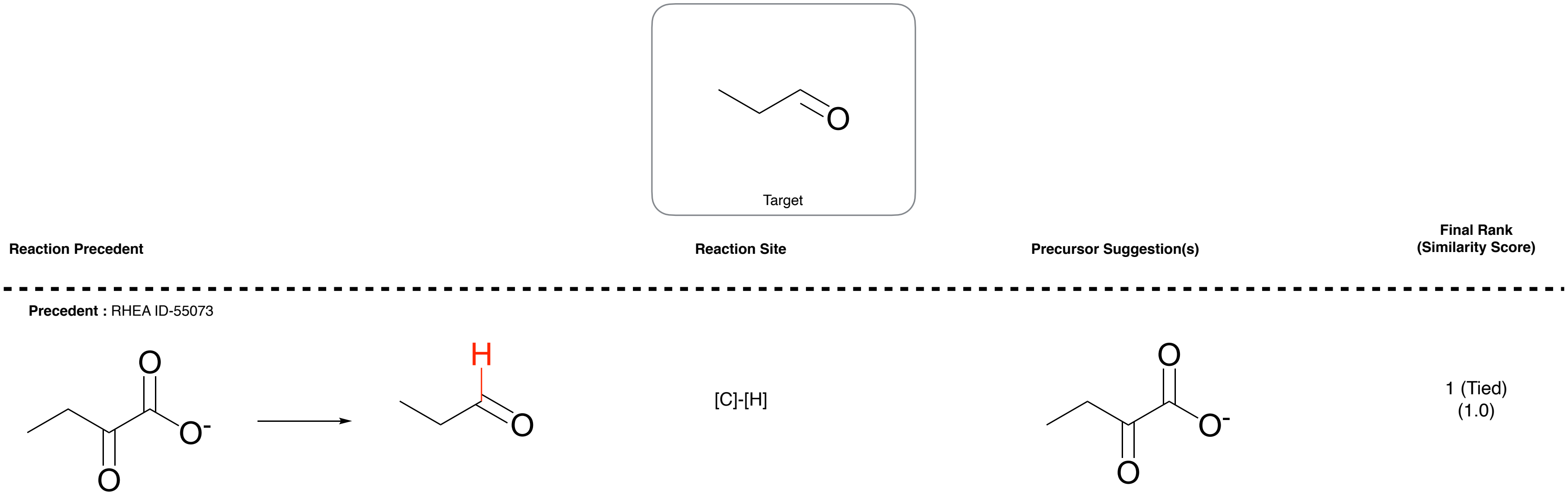

Figure S55: Single step retrosynthetic analysis for the intermediate compound 20 in 1-propanol synthesis. A 2-keto-acid decarboxylase converts the 2-keto acid to an aldehyde. An exact literature precedent is available to support this recommendation.

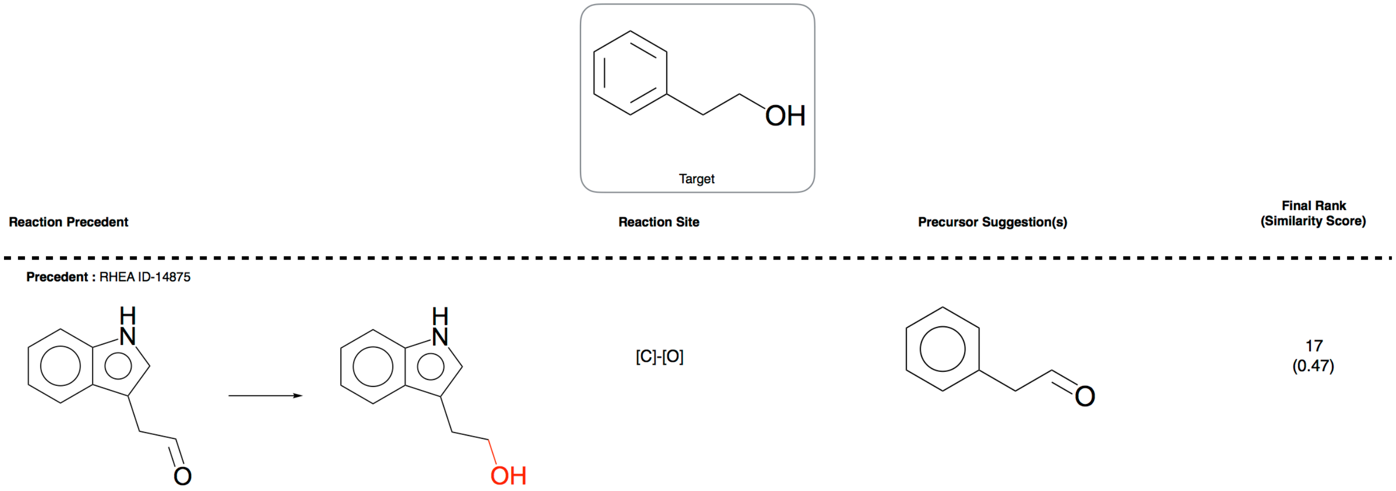

Figure S56: Single step retrosynthetic analysis for 2-phenylethanol synthesis (Compound 21). An alcohol dehydrogenase converts the aldehyde to alcohol. The evolution score for the proposed transformation is (0.996/1).

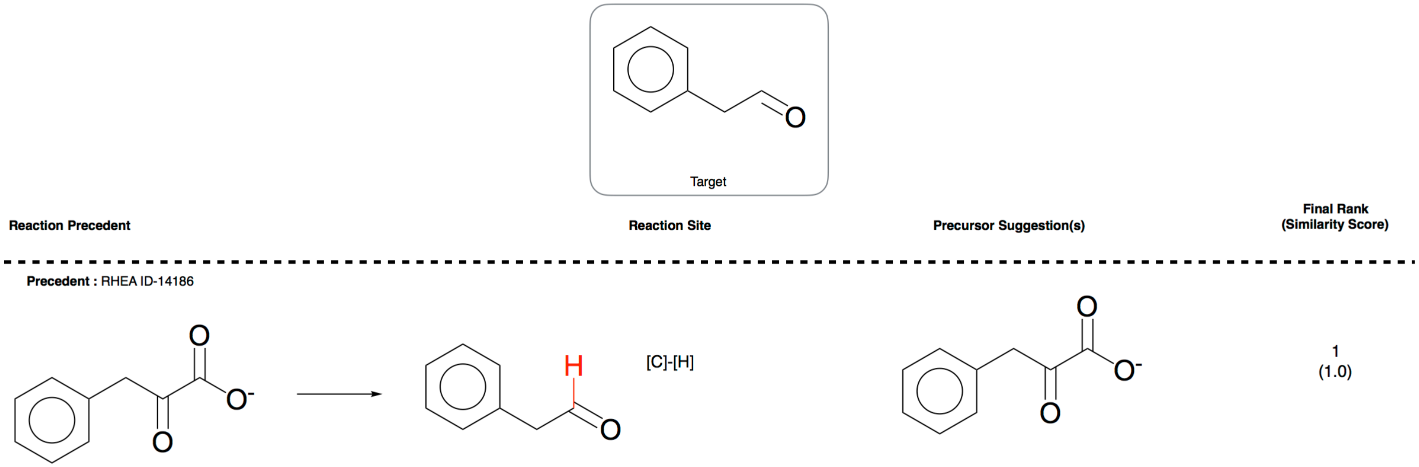

Figure S57: Single step retrosynthetic analysis for the intermediate compound 22 in 2-phenylethanol synthesis. A 2-keto-acid decarboxylase converts the 2-keto acid to an aldehyde. An exact literature precedent is available to support this recommendation.

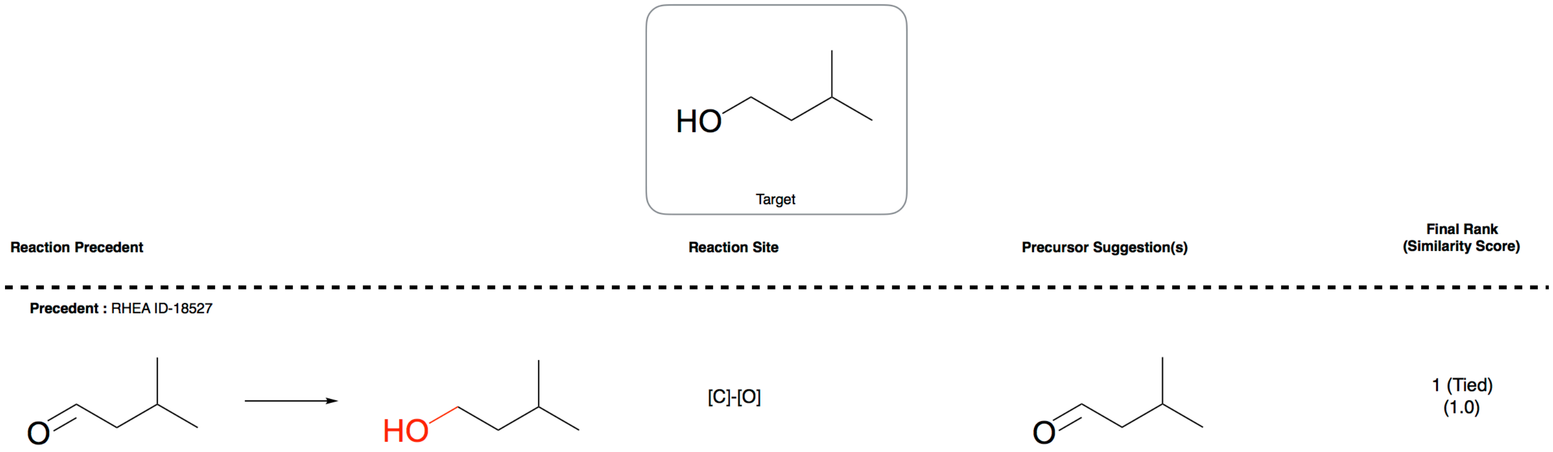

Figure S58: Single step retrosynthetic analysis for isobutanol synthesis (Compound 23). An alcohol dehydrogenase converts the aldehyde to alcohol. An exact literature precedent is available to support this recommendation.

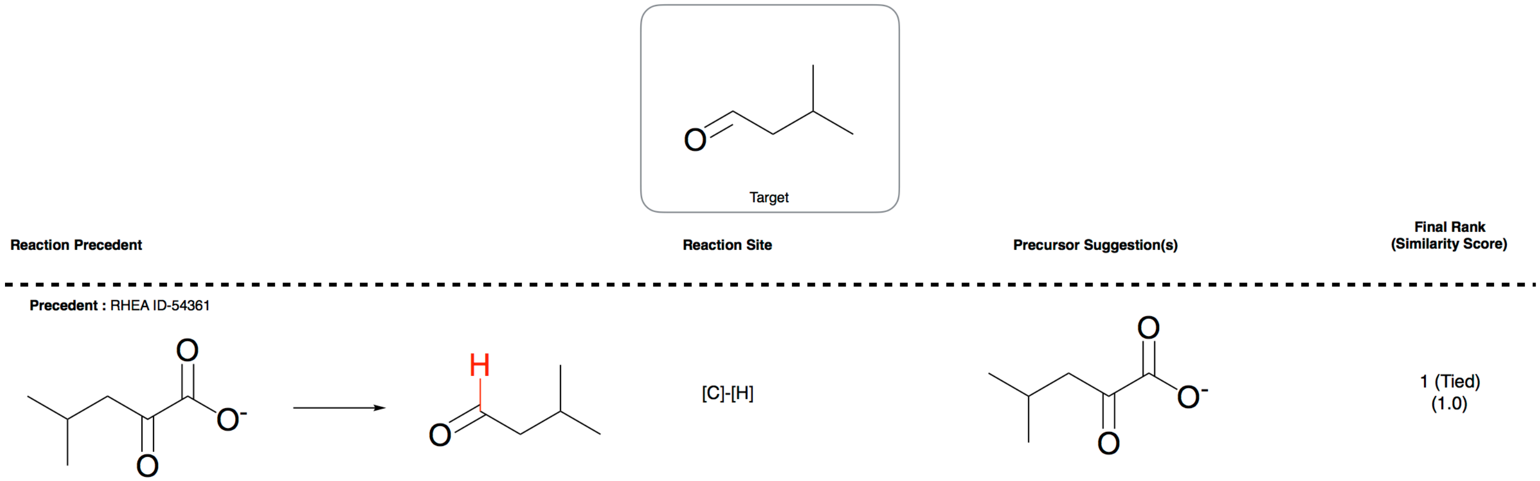

Figure S59: Single step retrosynthetic analysis for the intermediate compound 24 in isobutanol synthesis. A 2-keto-acid decarboxylase converts the 2-keto acid to an aldehyde. An exact literature precedent is available to support this recommendation.

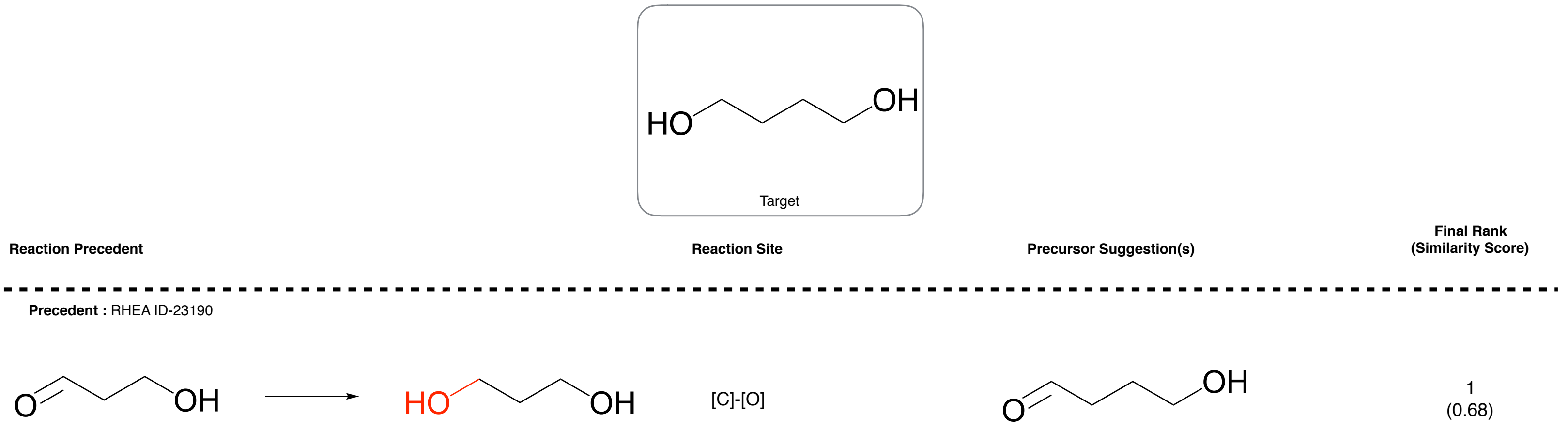

Figure S60: Single step retrosynthetic analysis for 1,4-butanediol (Compound 25). An alcohol dehydrogenase converts the aldehyde 4-hydroxybutyraldehyde into the desired alcohol. The evolution score for the proposed transformation is (0.996/1).

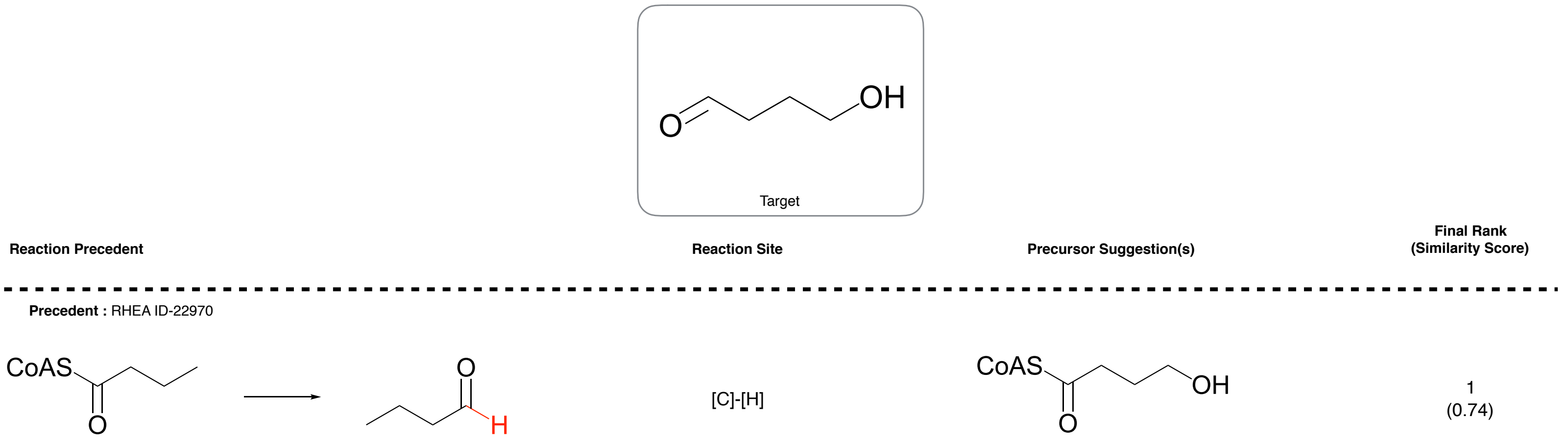

Figure S61: Single step retrosynthetic analysis for the intermediate compound 26 in 1,4-butanediol synthesis. A 4-hydroxybutyryl-CoA reductase catalyzes the reduction of 4-hydroxybutyryl CoA to 4-hydroxybutyraldehyde. The evolution score for the proposed transformation is (0.996/1).

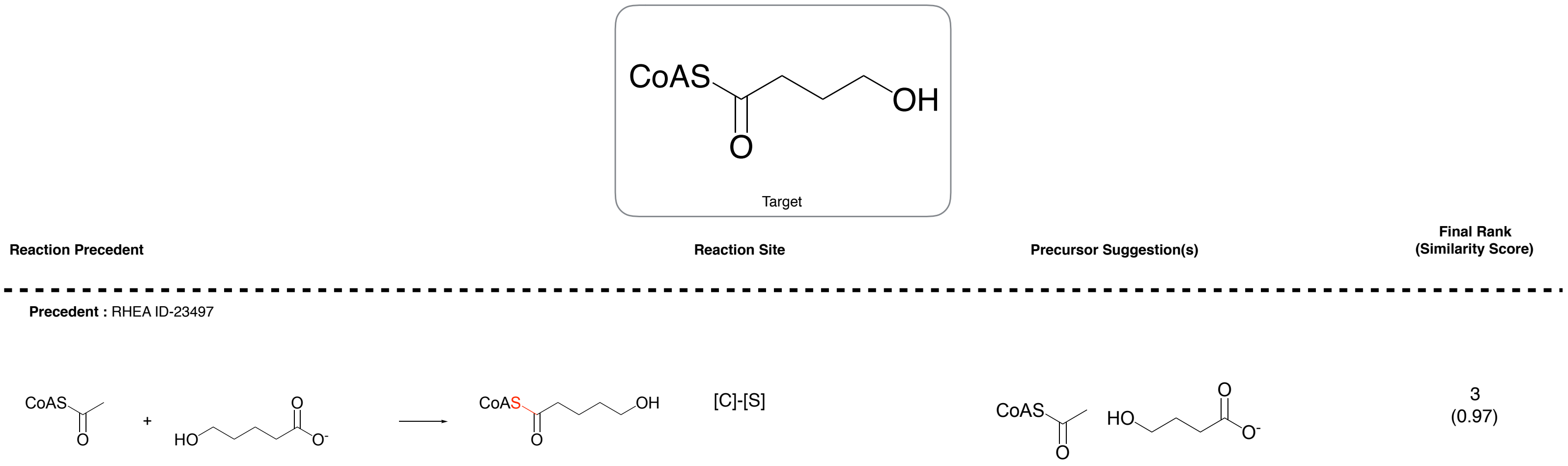

Figure S62: Single step retrosynthetic analysis for the intermediate compound 27 in 1,4-butanediol synthesis. A 4-hydroxybutyrl-CoA transferase loads 4-hydroxybutyrate onto Coenzyme A. The evolution score for the proposed transformation is (0.996/1).

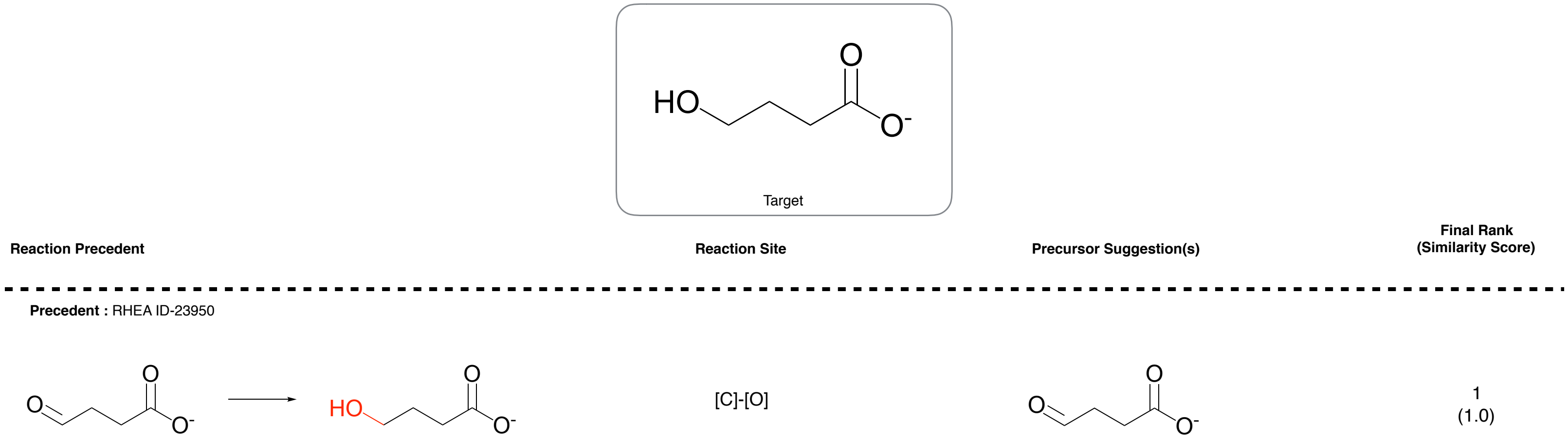

Figure S63: Single step retrosynthetic analysis for the intermediate compound 28 in 1,4-butanediol synthesis. A 4-hydroxybutyrate dehydrogenase converts the aldehyde succinyl semialdehyde into the alcohol 4-hydroxybutyrate. An exact literature precedent is available to support this recommendation.

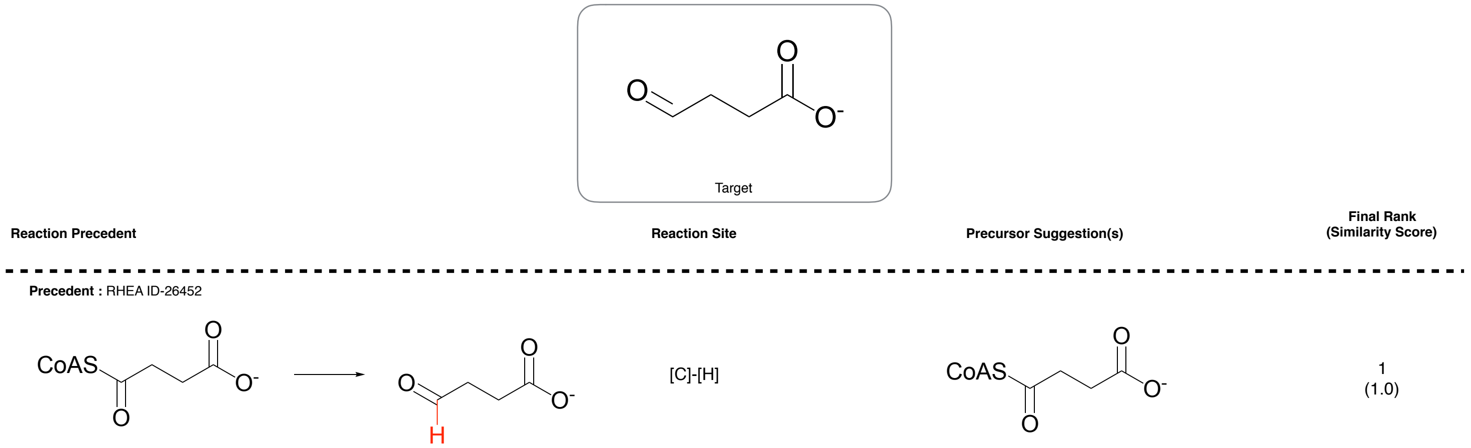

Figure S64: Single step retrosynthetic analysis for the intermediate compound 29 in 1,4-butanediol synthesis. Succinyl semialdehyde can be synthesized using succinyl CoA using CoA-dependent succinate semialdehyde dehydrogenase. An exact literature precedent is available to support this recommendation.

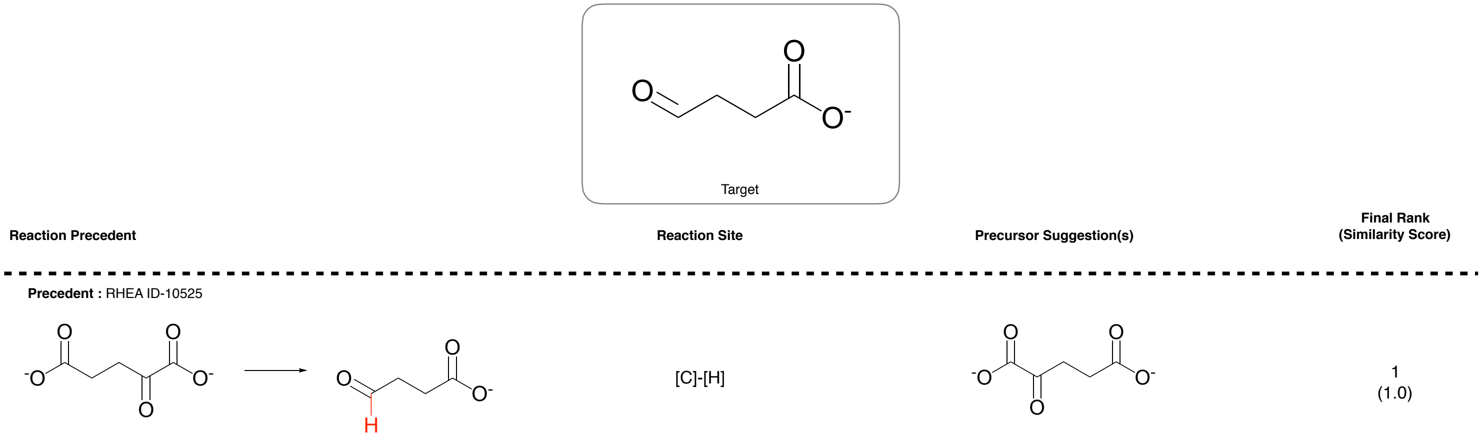

Figure S65: Single step retrosynthetic analysis for the intermediate compound 29 in 1,4-butanediol synthesis. Succinyl semialdehyde can be synthesized using α-ketoglutarate using 2-oxoglutarate decarboxylase. An exact literature precedent is available to support this recommendation.

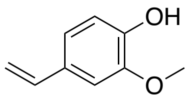

Target

Reaction Precedent	Reaction Site	Precursor Suggestion(s)	Final Rank (Similarity score)
**Precedent :** RHEA ID-33808 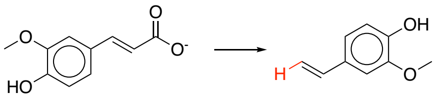	[C]-[H]	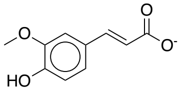	1 (1.0)

Figure S66: Single step retrosynthetic analysis for 4-hydroxy-3-methoxystyrene (Compound 32). A decarboxylase enzyme converts ferulic acid into the desired hydroxystyrene derivative. An exact literature precedent is available to support this recommendation.

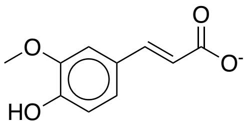

Target

Reaction Precedent	Reaction Site	Precursor Suggestion(s)	Final Rank (Similarity score)
**Precedent :** RHEA ID-20226 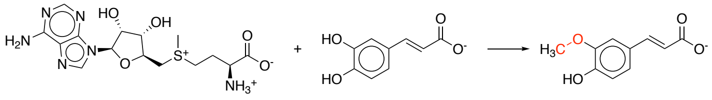	[C]-[O]	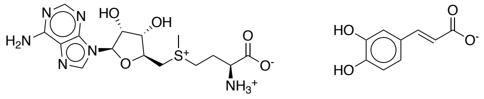	1 (1.0)

Figure S67: Single step retrosynthetic analysis for the intermediate compound 33 in hydroxystyrene derivative synthesis. A methyltransferase converts caffeic acid into ferulic acid. An exact literature precedent is available to support this recommendation.

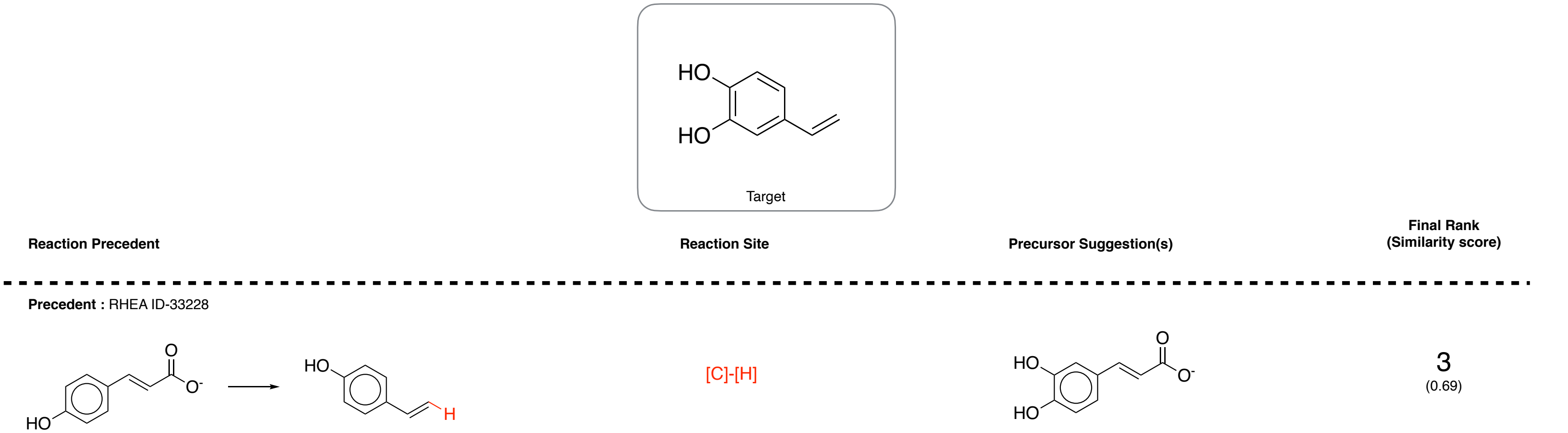

Figure S68: Single step retrosynthetic analysis for 3,4-dihydroxy styrene (Compound 34). A decarboxylase enzyme converts caffeic acid into the desired hydroxystyrene derivative. The evolution score for the proposed transformation is (0.99/1).

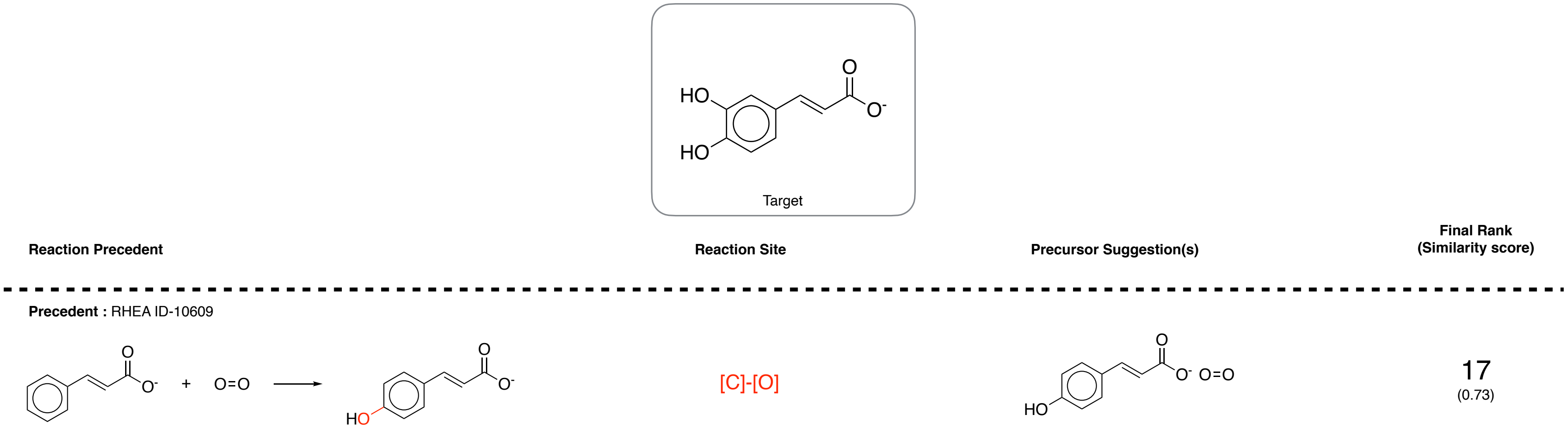

Figure S69: Single step retrosynthetic analysis for the intermediate compound 35 in hydroxystyrene derivative synthesis. A hydroxylase enzyme converts 4-coumaric acid into caffeic acid. The evolution score for the proposed transformation is (0.996/1).

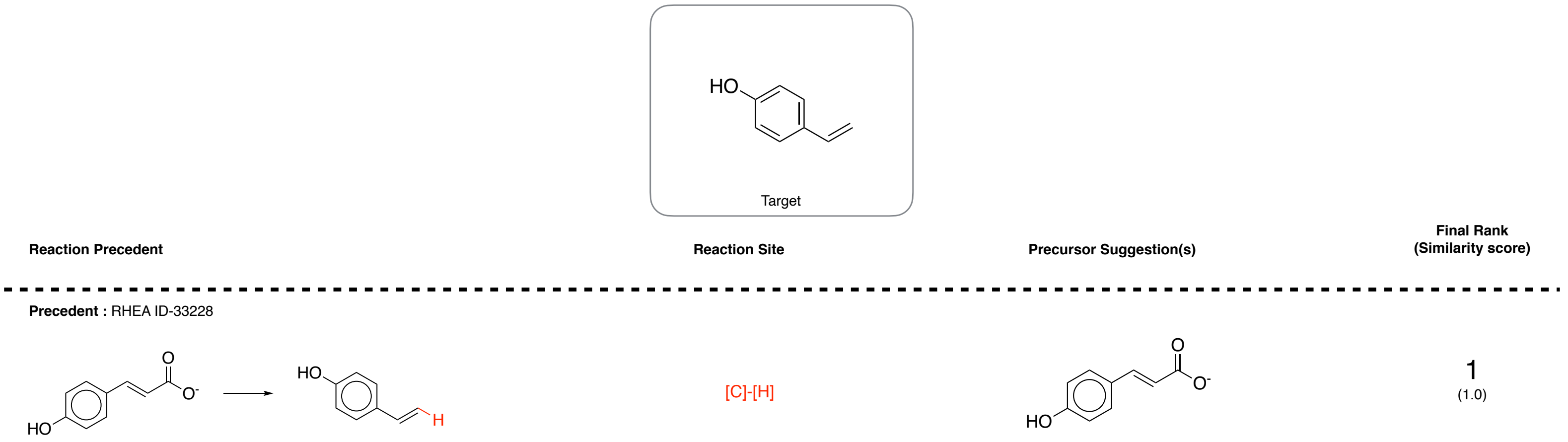

Figure S70: Single step retrosynthetic analysis for 4-hydroxystyrene (Compound 36). A decarboxylase enzyme converts 4-coumaric acid into the desired hydroxystyrene derivative. An exact literature precedent is available to support this recommendation.

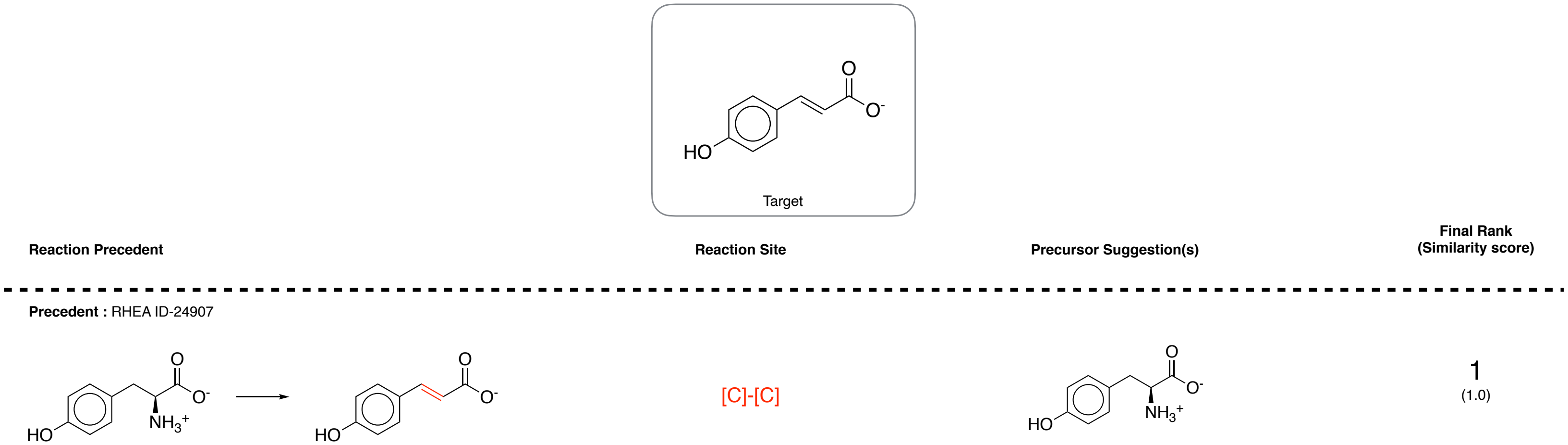

Figure S71: Single step retrosynthetic analysis for the intermediate compound 37 in hydroxystyrene derivative synthesis. Tyrosine ammonia lyase converts L-tyrosine to 4-coumaric acid. An exact literature precedent is available to support this recommendation.

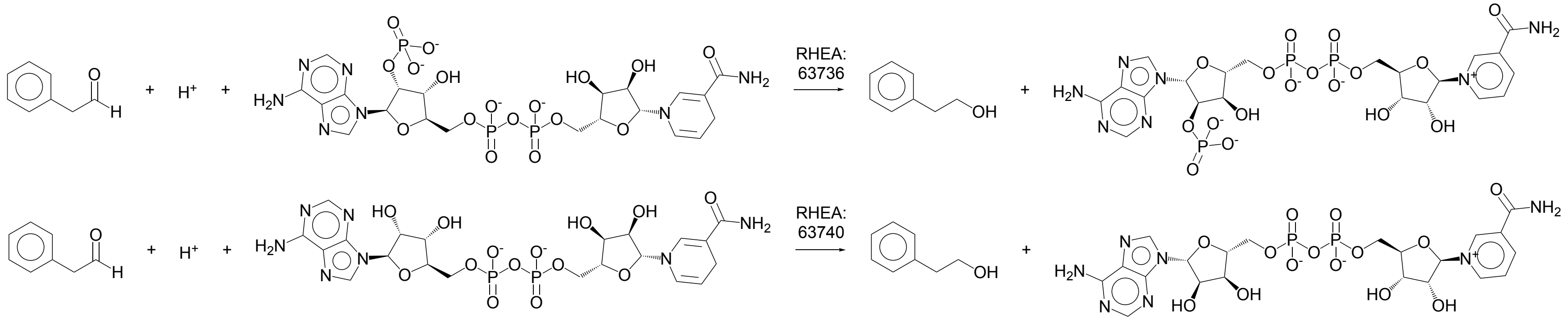

Figure S72: RHEA reactions 63736 and 63740 are missing from our knowledgebase. However, they are present in the complete version of RHEA

Table S7: The single-step retrosynthetic search for hydroxstyrene derivatives takes *O* (1 second) per step.

Product	Reactant	Enzyme Class	Time (s)
**32**	**33**	Decarboxylase	1.7
**33**	**35**	Methyltransferase	0.5
**34**	**35**	Decarboxylase	2.8
**35**	**37**	Hydroxylase	0.8
**36**	**37**	Decarboxylase	1.4
**37**	**38**	Tyrosine ammonia lyase	2.0

A)

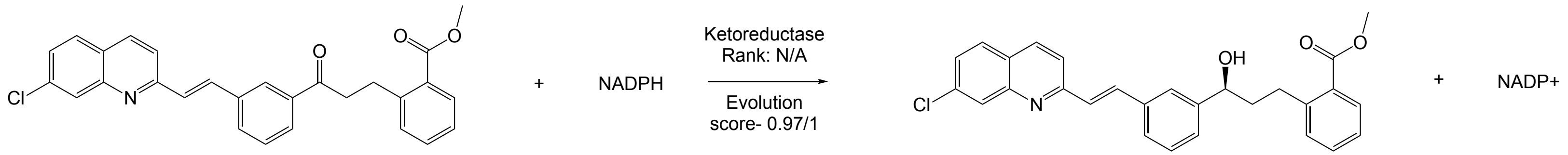

B)

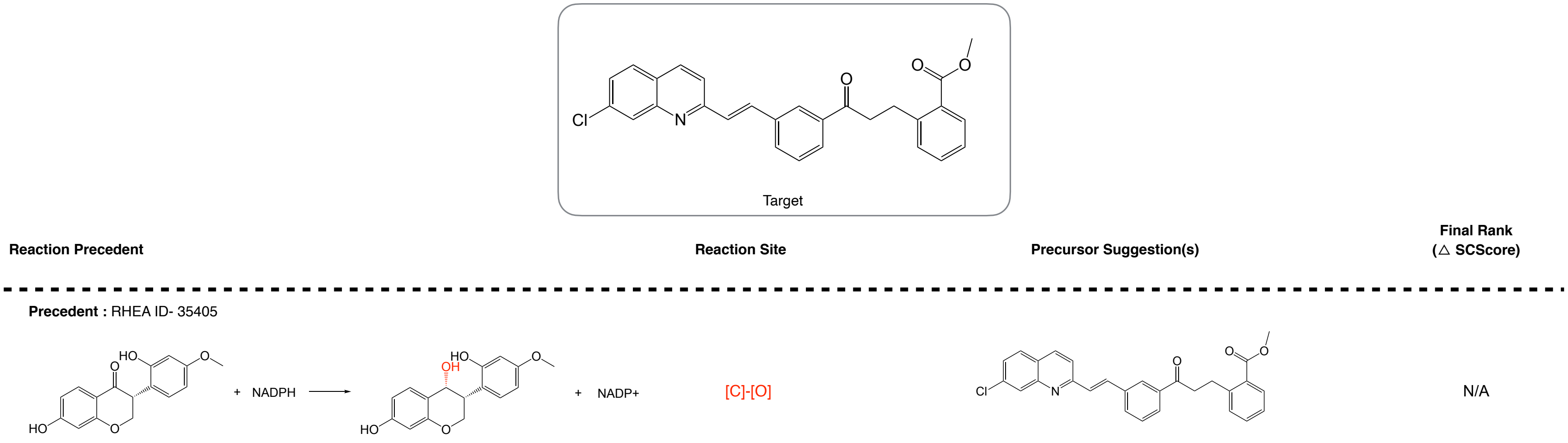

Figure S73: Poor applicability of the tool to enzymes outside the scope of current data set. Montelukast sodium manufacturing process uses a ketoreductase to catalyze the transformation from a ketone intermediate to a chiral alcohol. This suggestion is outside the scope of the top-50 similar molecules, and the search is expanded to top-500 similar molecules. The most chemically similar ketoreductase (RHEA: 35405) is identified using the algorithm. Its similarity rank is 173, and its evolution score is 0.97/1.

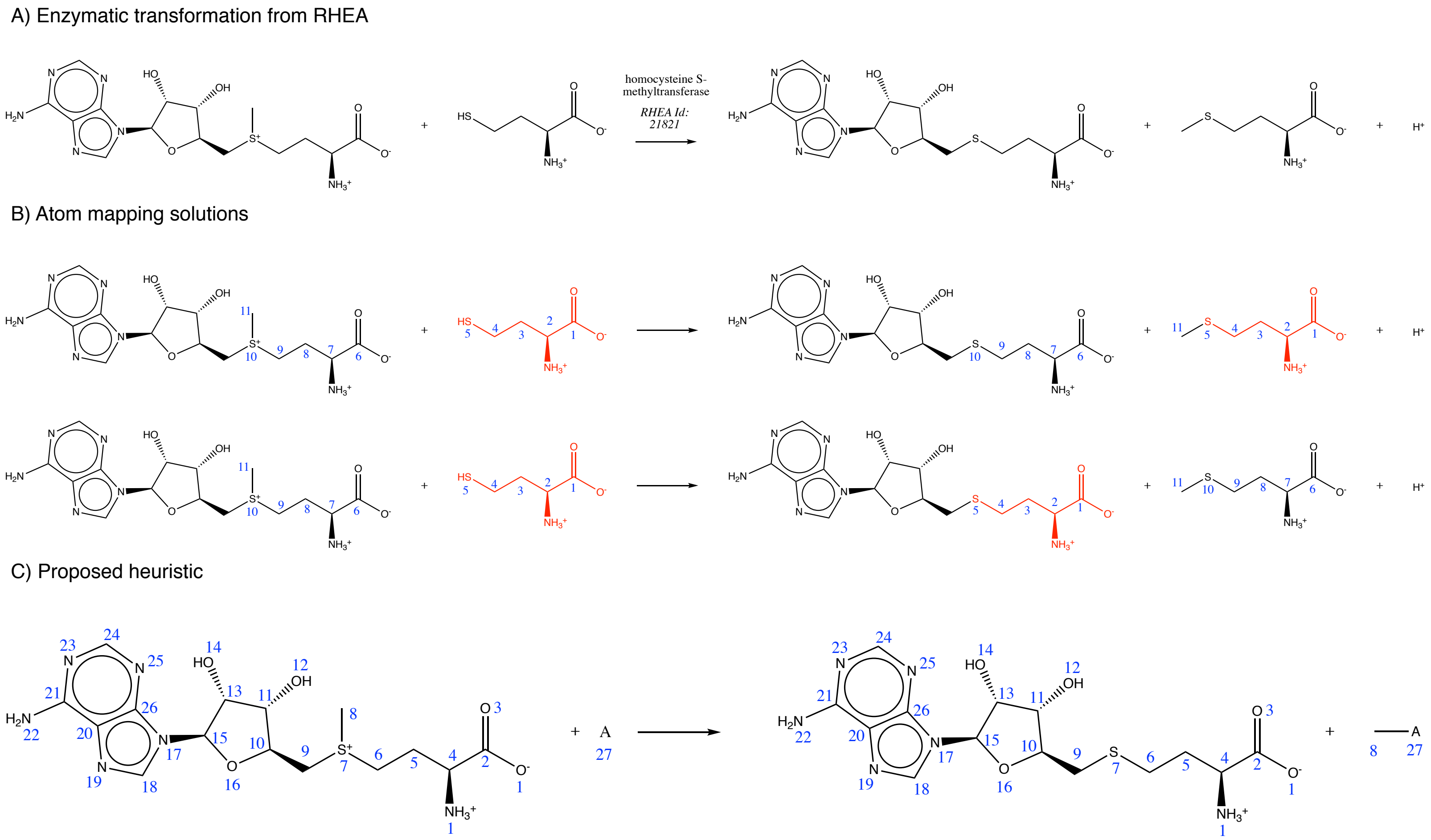

Figure S74: Ambiguous atom mapper output for an exemplary enzymatic transformation. (A) Enzymatic transformation (RHEA ID: 21821) corresponds to homocysteine S-methyltransferase. (B) Multiple solutions provided by atom mapper[6] (only selected atoms are labeled for clarity). Roughly 7% of the dataset have multiple, ambiguous atom mapping solutions. (C) Proposed heuristic corresponding to transformations associated with S-adenosyl-L-methionine (SAM) dependent methyltransferase enzymes can alleviate this ambiguity.

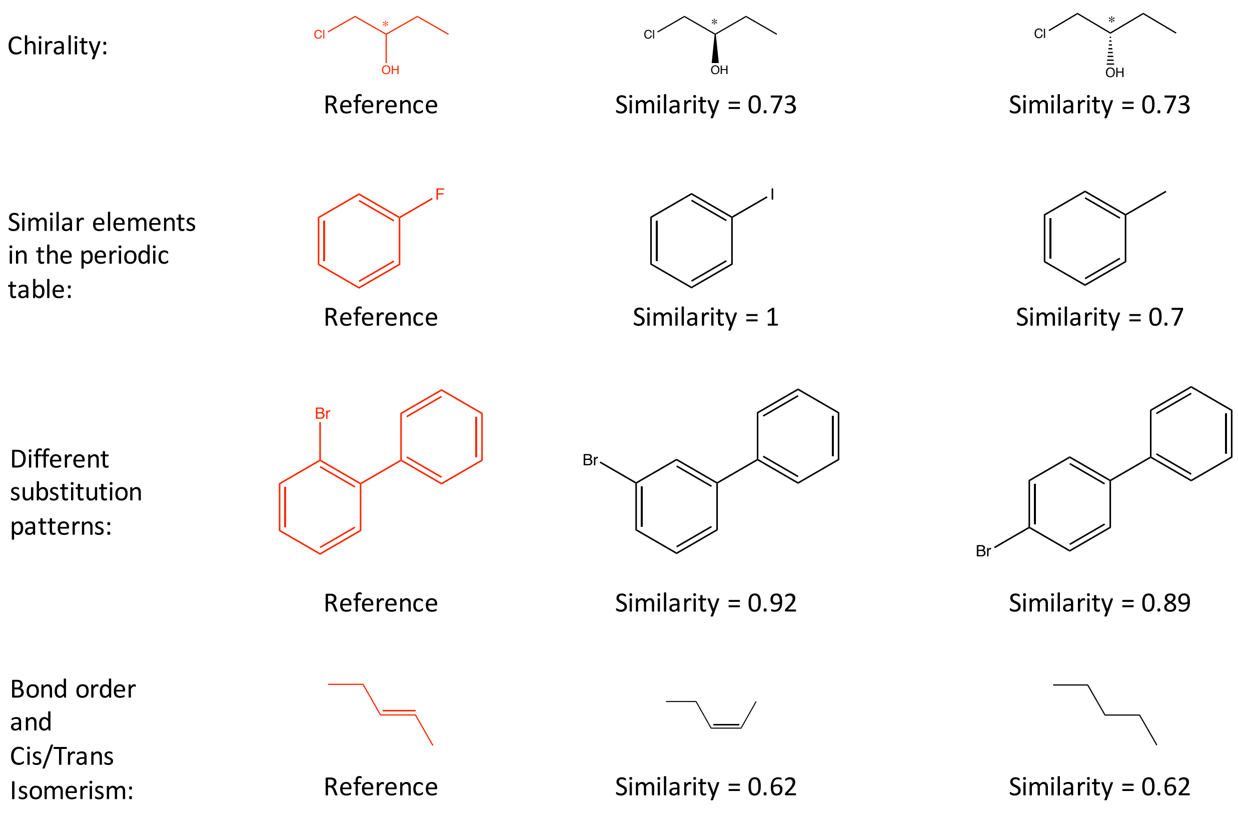

Figure S75: Chemical similarity is evaluated using RDKit's implementation of Morgan circular fingerprint (radius =2, using chirality and features) and Dice similarity. Results shown correspond to the final settings after hyperparameter optimization. Four important chemical features captured by the technique are shown (not exhaustive), and in each case, the reference molecule is highlighted in red and pairwise similarity is displayed below the molecule.

Table S8: Reaction processing to ensure compatibility with RDChiral.

Processing Step	Reactions Lost	Reactions Available
Atom mapped RHEA reaction SMILES		18064
Product enumeration + by-product and transport reactions removal	755	17309

Table S9: RDChiral shows promise for applications in enzymatic retrosynthesis. First, a retrosynthetic template is extracted from the given reaction. Second, the template is applied to the reaction product to determine whether its reactants are successfully recovered. As control, we show RDChiral's performance on selected 50,000 reactions from the US patent reaction database comprising organic transformations[1].

	**USPTO 50k Dataset**	**RHEA Enzymatic Reaction Database**
**Total # of reactions**	50,016 (100%)	17,309 (100%)
**Total number of successful reactions**	48,736 (97.4%)	12,349 (71.3%)

A) Consistent handling of the stereochemistry of leaving groups

Precedent reaction:

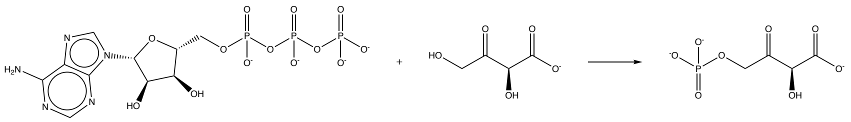

RDEnzyme:

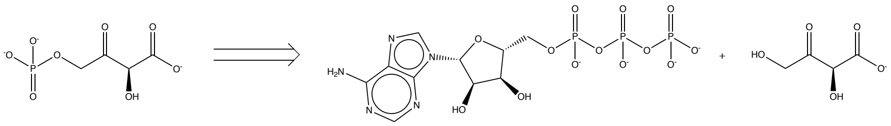

RDChiral:

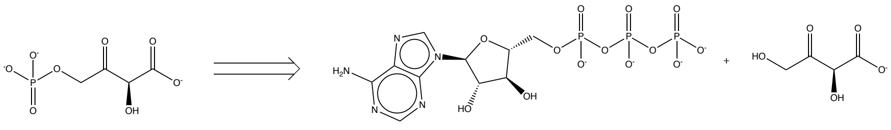

B) Stereo-isomerization reaction

Precedent reaction:

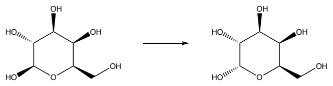

RDEnzyme:

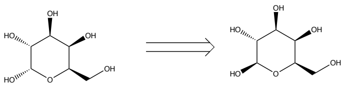

RDChiral:

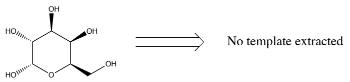

C) Destruction of chiral centers away from reaction centers (when not explicit in precedent reaction)

Precedent reaction:

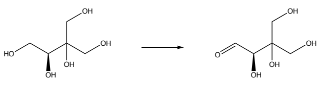

RDEnzyme:

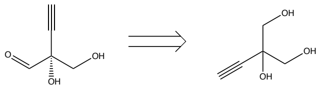

RDChiral:

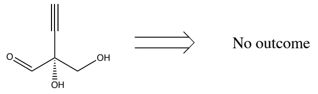

Figure S76: Additional features in RDEnzyme relevant to enzymatic retrosynthesis.

Table S10: RDEnzyme is evaluated to determine compatibility with RHEA enzymatic dataset.

	**RDChiral**	**RDEnzyme**
**Total # of reactions**	17,309 (100%)	17,309 (100%)
**Total number of successful reactions**	12,349 (71.3%)	15,690 (90.6%)

References:

(1) Coley, C. W.; Rogers, L.; Green, W. H.; Jensen, K. F. Computer-Assisted Retrosynthesis Based on Molecular Similarity. *ACS Cent. Sci.* **2017**, *3* (12), 1237–1245. https://doi.org/10.1021/acscentsci.7b00355.

(2) Coley, C. W.; Green, W. H.; Jensen, K. F. RDChiral: An RDKit Wrapper for Handling Stereochemistry in Retrosynthetic Template Extraction and Application. *J. Chem. Inf. Model.* **2019**, *59* (6), 2529–2537. https://doi.org/10.1021/acs.jcim.9b00286.

(3) Rhea- Annotated reactions database https://www.rhea-db.org/ (accessed 2019 -10 -01).

(4) Reetz, M. T. Controlling the Enantioselectivity of Enzymes by Directed Evolution: Practical and Theoretical Ramifications. *PNAS* **2004**, *101* (16), 5716–5722. https://doi.org/10.1073/pnas.0306866101.

(5) Buyable Compounds https://askcos.mit.edu/buyables/ (accessed 2021 -11 -23).

(6) Jaworski, W.; Szymkuć, S.; Mikulak-Klucznik, B.; Piecuch, K.; Klucznik, T.; Kaźmierowski, M.; Rydzewski, J.; Gambin, A.; Grzybowski, B. A. Automatic Mapping of Atoms across Both Simple and Complex Chemical Reactions. *Nat Commun* **2019**, *10* (1), 1–11. https://doi.org/10.1038/s41467-019-09440-2.
